# Genomic analyses provide insights into the evolution and salinity adaptation of halophyte *Tamarix chinensis*

**DOI:** 10.1093/gigascience/giad053

**Published:** 2023-07-26

**Authors:** Jian Ning Liu, Hongcheng Fang, Qiang Liang, Yuhui Dong, Changxi Wang, Liping Yan, Xinmei Ma, Rui Zhou, Xinya Lang, Shasha Gai, Lichang Wang, Shengyi Xu, Ke Qiang Yang, Dejun Wu

**Affiliations:** College of Forestry, Shandong Agricultural University, Taian 271018, China; College of Forestry, Shandong Agricultural University, Taian 271018, China; State Forestry and Grassland Administration Key Laboratory of Silviculture in the Downstream Areas of the Yellow River, Shandong Agricultural University, Taian 271018, China; Shandong Taishan Forest Ecosystem Research Station, Shandong Agricultural University, Taian 271018, China; College of Forestry, Shandong Agricultural University, Taian 271018, China; State Forestry and Grassland Administration Key Laboratory of Silviculture in the Downstream Areas of the Yellow River, Shandong Agricultural University, Taian 271018, China; Shandong Taishan Forest Ecosystem Research Station, Shandong Agricultural University, Taian 271018, China; College of Forestry, Shandong Agricultural University, Taian 271018, China; State Forestry and Grassland Administration Key Laboratory of Silviculture in the Downstream Areas of the Yellow River, Shandong Agricultural University, Taian 271018, China; Shandong Taishan Forest Ecosystem Research Station, Shandong Agricultural University, Taian 271018, China; College of Forestry, Shandong Agricultural University, Taian 271018, China; Shandong Provincial Academy of Forestry, Jinan 250014, China; College of Forestry, Shandong Agricultural University, Taian 271018, China; College of Forestry, Shandong Agricultural University, Taian 271018, China; College of Forestry, Shandong Agricultural University, Taian 271018, China; College of Forestry, Shandong Agricultural University, Taian 271018, China; College of Forestry, Shandong Agricultural University, Taian 271018, China; College of Forestry, Shandong Agricultural University, Taian 271018, China; College of Forestry, Shandong Agricultural University, Taian 271018, China; State Forestry and Grassland Administration Key Laboratory of Silviculture in the Downstream Areas of the Yellow River, Shandong Agricultural University, Taian 271018, China; Shandong Taishan Forest Ecosystem Research Station, Shandong Agricultural University, Taian 271018, China; Shandong Provincial Academy of Forestry, Jinan 250014, China

**Keywords:** *Tamarix chinensis*, genome assembly, genome evolution, transcriptome, salinity adaptation

## Abstract

**Background:**

The woody halophyte *Tamarix chinensis* is a pioneer tree species in the coastal wetland ecosystem of northern China, exhibiting high resistance to salt stress. However, the genetic information underlying salt tolerance in *T. chinensis* remains to be seen. Here we present a genomic investigation of *T. chinensis* to elucidate the underlying mechanism of its high resistance to salinity.

**Results:**

Using a combination of PacBio and high-throughput chromosome conformation capture data, a chromosome-level *T. chinensis* genome was assembled with a size of 1.32 Gb and scaffold N50 of 110.03 Mb. Genome evolution analyses revealed that *T. chinensis* significantly expanded families of *HAT* and *LIMYB* genes. Whole-genome and tandem duplications contributed to the expansion of genes associated with the salinity adaptation of *T. chinensis*. Transcriptome analyses were performed on root and shoot tissues during salt stress and recovery, and several hub genes responding to salt stress were identified. *WRKY33*/*40, MPK3*/*4*, and *XBAT31* were critical in responding to salt stress during early exposure, while *WRKY40, ZAT10, AHK4, IRX9*, and *CESA4*/*8* were involved in responding to salt stress during late stress and recovery. In addition, *PER7/27/57/73* encoding class III peroxidase and *MCM3/4/5/7* encoding DNA replication licensing factor maintained up/downregulation during salt stress and recovery stages.

**Conclusions:**

The results presented here reveal the genetic mechanisms underlying salt adaptation in *T. chinensis*, thus providing important genomic resources for evolutionary studies on tamarisk and plant salt tolerance genetic improvement.

## Introduction

It is reported that around 7% of the global land and approximately one-third of global irrigated lands have become salt affected, and the salinity of soils seriously limits plant growth and crop production [1]. Salt stress, one of the most detrimental environmental stressors, mainly causes osmotic stress and ionic toxicity in plants [2, 3]. To cope with adverse effects, plants adapt to various mechanisms, including activating the osmotic stress pathway, regulating ion homeostasis, and mediating hormone signaling, resulting in metabolic and physiological responses [4–6]. After exposure to salt stress, plants first sense signals through multiple receptors or sensors, such as Ca^2+^-permeable channel glutamate receptor (GLR) [7] and cyclic nucleotide-gated ion channel (CNGC) for Na^+^ permeation [8], and then elevated cellular Ca^2+^ induced reactive oxygen species (ROS) and activation of several signal molecules, including 14-3-3–like proteins, calcineurin B-like proteins (CBLs), calcium-dependent protein kinases (CDPKs), and calcineurin B-like interacting protein kinases [4]. Subsequently, ROS-activated mitogen-activated protein kinases (MAPKs), in combination with the activated signal molecules, initiated several transcription factors like WRKYs, resulting in the transcription of multiple stress-responsive genes [9–11]. Furthermore, several ion carriers or channels played essential roles in ion homeostasis, such as potassium channel AKT1 [12], stelar K^+^ outward rectifying channel (SKOR) [13], calcium-activated outward-rectifying potassium channel 1 (TPK1) [14], sodium transporter HKT1 [15], sodium/hydrogen exchangers (NHXs) [16], cation/H^+^ antiporters (CHXs) [17], and chloride channel proteins (CLCs) [18]. Recent evidence demonstrated that salt stress could inhibit the cell cycle by controlling cell cycle regulators [19]. However, our understanding of the mechanisms underlying plant salt resistance is still developing.

Halophytes, distinct from glycophytes, which represent most salt-sensitive plants, exhibit high salt tolerance and can survive in soils with high salt concentrations (>200 mM NaCl) [20, 21]. Therefore, it is vital to understand the genomic information and mechanisms underlying their tolerance to salt stress, which may help to exploit and utilize these resources to cope with increasing saline soils. *Tamarix* (Tamaricaceae, Caryophyllales) is an Old World genus containing approximately 90 species, grown widely in arid and semiarid areas of Eurasia, Africa, the ancient Mediterranean Sea, and northwestern China [22, 23]. Among *Tamarix* species, the woody halophyte *Tamarix chinensis* Lour (saltcedar or tamarisk; NCBI:txid189791), a deciduous shrub or tree, is a pioneer species of the coastal saline wetland ecosystem in northern China and is a major component of the circumlittoral shelter forest known as the coastguard [24–26]. In addition, because of its high tolerance to salt stress and rapid growth, *T. chinensis* has been considered an ideal model for investigating plant salt tolerance mechanisms [27–29]. However, the scarcity of reference genome sequences in *T. chinensis* largely hampers a better understanding of the underlying mechanisms of its high salinity adaptation.

Dissecting the whole genome of plants for different salt tolerance genomic resources is a pivotal approach to investigating plant adaptation mechanisms to salt stress. With rapid advances in high-throughput genome sequencing, increasing numbers of salt-tolerant plant genomes have been dissected, and their molecular adaptation to salinity environments has been clarified [30–36]. Here, we present a genomic investigation of *T. chinensis* to elucidate the underlying mechanism of its high resistance to salinity. This study will provide important genomics resources for evolutionary studies on tamarisk and the genetic improvement of plant salt tolerance.

## Materials and Methods

### Plant material

Diploid *T. chinensis* Lour. ‘Lucheng No. 1’ (2n = 24) (Supplementary Fig. S1) was conserved in the Forestry Experimental Station of Shandong Agricultural University, Taian, China (117.15 E, 36.17 N). Total DNA and RNA were isolated from the healthy, tender shoots according to previously described methods [37]. Approximately 5 g of fresh and tender shoots were fixed with 1% formaldehyde and then used to extract intact nuclei to construct the high-throughput chromosome conformation capture (Hi-C) library as previously described [37, 38].

### Genome survey

Flow cytometry analysis evaluated the size of *T. chinensis* ‘Lucheng No. 1’ genome by comparing it to the genome size of *Zea mays* ‘B73’ (an internal reference, approximately 2.32 Gb) [39]. Fresh and tender shoots collected immediately were subjected to nuclei extraction and DNA staining by a Sysmex CyStain PI Absolute P kit according to the manufacturer's recommended protocols. The nuclear size was determined by a Sysmex CyFlow Cube6 flow cytometer with at least 10,000 nuclei counts analyzed per plant. Flow cytometer output was analyzed using FlowJo v. 10.5.3 (BD Biosciences).

Genome size of *T. chinensis* ‘Lucheng No. 1’ was assessed using a *k*-mer method [40] based on Illumina short reads. The DNA library with a 300-bp insert size was constructed and sequenced on an Illumina NovaSeq6000 platform (KeGene) with a PE-150 module, yielding around 44.63 Gb of raw-data bases. After trimming by Trimmomatic (RRID:SCR_011848) v. 0.38 [41], around 44.23 Gb of validated bases were generated. A 30-mer frequency analysis was performed using Jellyfish (RRID:SCR_005491) v. 2.3.0 [40], resulting in a depth of 22 for the highest peak. *T. chinensis* genome size was determined by genome size = number of *k*-mer/average *k*-mer depth.

### Genome sequencing

A 40-kb insert size SMRTbell library was constructed by SMRTbell Express Template Prep Kit 2.0 and sequenced on PacBio Sequel II (Pacific Biosciences) using Chemistry 2.0 for 15 hours per SMRT Cell 8 M. The process produced more than 213 Gb of subread bases, including more than 7.41 million subreads with an average length of 28.81 kb.

Two Hi-C libraries were prepared according to the *in situ* Hi-C library preparation protocol for plants [42]. Briefly, cross-linked nuclear chromatin was first treated with DpnII restriction enzyme (New England Biolabs). Next, nuclear chromatin was incorporated with biotin-14-dATP for end repair, ligation, and DNA purification. The recovered ligations were sequenced on the Illumina NovaSeq6000 platform (KeGene), yielding approximately 132.9 Gb high-quality bases.

The RNA sequencing (RNA-seq) library was prepared by the TruSeq RNA Sample Preparation Kit (Illumina) and sequenced on the Illumina NovaSeq6000 instrument, generating around 9.85 Gb bases for subsequent gene prediction.

### Genome assembly

The PacBio subreads were corrected, trimmed, and assembled using Canu (RRID:SCR_015880) v. 2.1 [43] under the following parameters: correctedErrorRate = 0.045 and minReadLength = 2000, resulting in a primary assembly of 2.16 Gb size with 5,299 contigs exhibiting an N50 size of 4.43 Mb. The preliminary assembly was subjected to Purge_Dups (RRID:SCR_021173) v. 1.0.1 pipeline [44] to remove the duplications and obtain the purged primary sequence. The process yielded a genome assembly comprising 342 contigs covering 1.32 Gb, represented by a contig N50 length of approximately 11.93 Mbp. The purged primary genome assembly was first polished by GCpp v. 2.0.2 (Pacific Biosciences) using PacBio subreads and subsequently polished by Pilon (RRID:SCR_014731) v. 1.23 [45] using Illumina short reads. The polished genome assembly was subjected to genome assembly quality assessment, BUSCO (RRID:SCR_015008) v. 5.0.0 [46], with the embryophyta_odb10 dataset to assess assembly completeness, showing 97.1% BUSCOs being captured entirely in the genome sequence.

As previously described tools [37], Hi-C data were used for chromosomal-level assembly. In brief, using Juicer pipeline v. 1.6 [47], Hi-C data were aligned to the polished assembly to produce duplicate free contact maps. Subsequently, Hi-C maps were subjected to 3D-DNA pipeline v. 201,013 [48] to construct the chromosomal-level genome assembly. The resulting assembly was imported into Juicebox v. 2.13.07 [49] for final assembly manual review and refinement. The Hi-C contact maps for final assembly were visualized using HiCPlotter v. 0.6.6 [50]. All software was executed with default parameters.

### Genome assembly quality assessment

Four approaches evaluated genome assembly quality: BUSCO, DNA, RNA sequencing data analysis, and Merqury quality statistics. First, BUSCO evaluated the completeness of the final assembly with the embryophyta_odb10 dataset. Next, Illumina and PacBio data were aligned to the assembly using Bowtie2 v. 2.4.2 [51] and pbmm2 v. 1.4.0 (Pacific Biosciences), and mapping rates were calculated. Then, RNA-seq data were subjected to Trinity v. 2.11.0 [52] to obtain full-length transcripts that were subsequently mapped to the assembly by BLAT v. 35 [53] to calculate genome mapping rates. Finally, Merqury (RRID:SCR_022964) v. 1.3 [54] with the parameter k = 25 estimated the assembly quality value (QV).

### Genome annotation

EDTA (RRID:SCR_022063) v. 2.0.0 [55] and RepeatMasker (RRID:SCR_012954) v. 4.07 [56] were integrated to identify repeat elements. MAKER (RRID:SCR_005309) pipeline v. 3.01.03 [57] was used to predict protein-coding genes. First, the Trinity assembled transcripts were subjected to PASA v. 2.4.1 [58] to generate high-quality transcripts, which were then used to train *ab initio* gene predictors, including SNAP (RRID:SCR_007936) [59], GENEMARK (RRID:SCR_011930) v. 4.68 [60], and AUGUSTUS (RRID:SCR_008417) v. 3.3.3 [61]. Subsequently, coding evidence from *ab initio* gene predictors was integrated using the MAKER pipeline, resulting in a comprehensive set of protein-coding genes. To improve gene annotation, the resulting gene models with an annotation edit distance (AED) score <0.2 were selected and imported into SNAP, GENEMARK, and AUGUSTUS programs for the second round of data retraining. The homology gene models were predicted using Exonerate v. 2.2.0 [62] by mapping the protein sequences of *Beta vulgaris* [63], *Spinacia oleracea* [64], *Vitis vinifera* [65], *Arabidopsis thaliana* [66], *Solanum lycopersicum* [67], *Populus trichocarpa* [68], and *Oryza sativa* [69] to the assembly. Finally, PASA transcripts, homology gene models, and retrained gene models were imported into the MAKER program to obtain the final protein-coding genes.

The function of the predicted genes was annotated using InterProScan (RRID:SCR_005829) v. 5.48–83.0 [70] by searching against InterPro database v. 83.0 [71]. In addition, predicted genes were functionally annotated by scanning the nonredundant protein (nr) of NCBI and SwissProt databases using blastp v. 2.10.1 with the following parameters: E-value <1e–5, coverage ≥50%, and identity ≥30%. KEGG annotation was performed using KofamScan [72] with default parameters.

### Phylogenetic analyses

According to the previous phylogenetic study, Caryophyllales can be divided into 5 subclades: PHYT, PORT, AMAR, CARY, and NCORE, with *T. chinensis* belonging to the NCORE subclade [73]. Therefore, we selected 26 species in the NCORE subclade and 5 other species, including *B. vulgaris, S. oleracea, V. vinifera, S. lycopersicum*, and *O. sativa*, as the outgroup to perform all versus all homology searches and orthology inference from coding sequences. The phylogenetic orthology inference was made using a modified phylome approach [74].

Comparative genomics analysis was performed on 12 plant species with genomic data, including 8 Caryophyllales species and 3 outgroups (*V. vinifera, A. thaliana*, and *O. sativa*). OrthoFinder (RRID:SCR_017118) v. 2.5.4 [75] made phylogenetic orthology inference. One-to-one orthologous genes were subjected to MAFFT (RRID:SCR_011811) v. 7.471 [76] for multiple sequence alignment. A phylogenetic tree was constructed by RAxML (RRID:SCR_006086) v. 8.2.12 [77] using the GTRCAT module with 200 bootstrap replicates. Species divergence time was inferred using PAML (RRID:SCR_014932) v. 4.9j [78]. For the first phylogenetic tree, 4 secondary calibration time points were set, including 14–56 mega-annum (Ma) ago between *Rumex palustris* and *Rheum nobile*, 24–53 Ma ago between *B. vulgaris* and *S. oleracea*, 25–94 Ma ago within *Persicaria virginiana* branch, and 148–173 Ma ago within root node. Three secondary calibration time points were set for the second phylogenetic tree, including 53.4–78.9 within the *Hylocereus undatus* branch, 109–123.5 Ma ago between *A. thaliana* and *V. vinifera*, and 148–173 Ma ago in the root node. The time of divergence between species was retrieved from the TimeTree database [79]. Analysis of gene family expansion and contraction was performed by CAFÉ v. 5.0 [80] with the following parameters: lambda -s -p 0.05.

### Whole-genome duplication events inference

We used synonymous substitution rates (*K_s_*), distribution of paralog gene pairs, and interspecies syntenic relationships to identify putative whole-genome duplication (WGD) events in the evolutionary history of *T. chinensis*. The *K_s_* distribution of paralog gene pairs was analyzed using WGD v. 1.1 [81]. The *K_s_* distribution was subjected to the BGMM module in WGD for mixed-model fitting, resulting in putative WGD peaks. In addition, the DupGen_finder pipeline was used to classify gene duplications [82] with default parameters. The number of substitutions per nonsynonymous site (*K_a_*), *K_s_*, and *K_a_*/*K_s_* scores between each paralog gene pair was calculated by KaKs_Calculator v. 2.0 [83] with the YN model after constructing a codon alignment by PAL2NAL v. 14.0 [84].

For interspecies collinearity analysis between *T. chinensis* and *V. vinifera*, the top 10 hits of each protein from all versus all sequence alignments between 2 species were imported into MCScanX [85] for collinearity analysis. Collinearity regions were visualized by JCVI v. 1.0.5 (https://zenodo.org/record/31631).

### Transcriptome profiling of root and shoot during salt stress and recovery

The annual shoots were collected and cut into cuttings with a length of 20 cm from *T. chinensis* ‘Lucheng No. 1’ on 10 February 2021. After washing with running tap water overnight, the cuttings were placed in three 58-cm × 33-cm × 15-cm containers, with 20 cuttings per container, and maintained in a hydroponic medium (half-strength Hoagland solution). As previously described, the cuttings were incubated in a growth chamber [86]. The medium was refreshed every 7 days. After 2 months of culture, healthy 18- to 20-cm cutting clones with root lengths of 20–35 cm were selected and subjected to NaCl stress. Before NaCl stress was applied, the cutting clones were pretreated in a medium containing 200 mM NaCl for 2 hours to avoid salt shock [87]. The cutting clones were grown for 7 days on a hydroponic medium with 300 mM NaCl before transferring to the hydroponic medium without NaCl for 35 days to recover. Seven time points for sample collection were selected to cover early salt stress (including 300 mM NaCl stressed 0.5, 3, 5, and 8 hours) and late salt stress and recovery (including 300 mM NaCl stressed 7 days and 35 days of recovery). Subsequently, 54 samples covering the 7 time points with 3 biological replicates per condition were harvested.

Total RNA from the roots and shoots was isolated according to previously described methods [37]. The RNA-seq library was prepared by the Illumina TruSeq RNA Sample Preparation Kit and sequenced on the Illumina NovaSeq6000 instrument using a PE-150 module. HiSAT2 (RRID:SCR_015530) v. 2.2.1 was used to map RNA-seq data to the *T. chinensis* genome, and featureCounts v. 2.0.3 [88] was used to quantify gene abundance. Principal coordinates analysis of gene expressions was performed using vegan v. 2.6–4 package in R v. 4.2. Integration of 4 differential analysis methods, including DEseq2 (RRID:SCR_015687) v. 1.34.0 [89], Limma (RRID:SCR_010943) v. 3.52.2 [90], ROTS v. 1.24.0 [91], and edgeR (RRID:SCR_012802) v. 3.38.4 [92], was performed to determine differentially expressed genes (DEGs), and all DEGs must satisfy |log_2_ (fold change)| ≥1 and false discovery rate ≤0.05. An intersection analysis of DEGs among different comparisons was performed by TBtools (RRID:SCR_023018) v. 1.098775 [93]. Dynamic gene expression analysis was performed by TCseq v. 1.22.0 package in R. Analysis of GO and KEGG categories enrichment was carried out by TBtools with whole-genome gene sets as background and a *q* value ≤0.05 as statistically significant.

Among plant species, *Arabidopsis* has the most abundant protein–protein interaction (PPI) data available, and most PPIs have been experimentally determined, so the protein sequences from the DEGs were BLAST searched against the *A. thaliana* protein database for homolog identification. The best homology hits were retrieved and subjected to construct PPI network using the STRING (https://string-db.org/) database with a confidence score cutoff of 0.4. The subnetwork and hub genes were identified by CytoHubba in Cytoscape (RRID:SCR_003032) v. 3.9.1 [94, 95].

### Quantitative real-time reverse-transcription polymerase chain reaction analysis

A randomly selected 27 DEGs were verified by quantitative real-time reverse-transcription polymerase chain reaction (qRT-PCR). PCR assays were performed on a Bio-Rad CFX Connect Real-Time instrument according to the procedure previously described [96]. Each sample was performed in 3 independent biological replicates. Relative abundance was quantified by normalizing it to the reference gene *TIF* [27].

## Results

### A chromosome-level *T. chinensis* genome assembly

The *k*-mer frequency and flow cytometry analyses were performed to assess the genome size of *T. chinensis*. The *k*-mer frequency analysis showed that the estimated genome size was about 1.45 Gb (Supplementary Fig. S2A), close to the flow cytometry results (Supplementary Fig. S2B). Thus, we evaluated the genome size of *T. chinensis* as 1.45 Gb.

**Figure 1:**
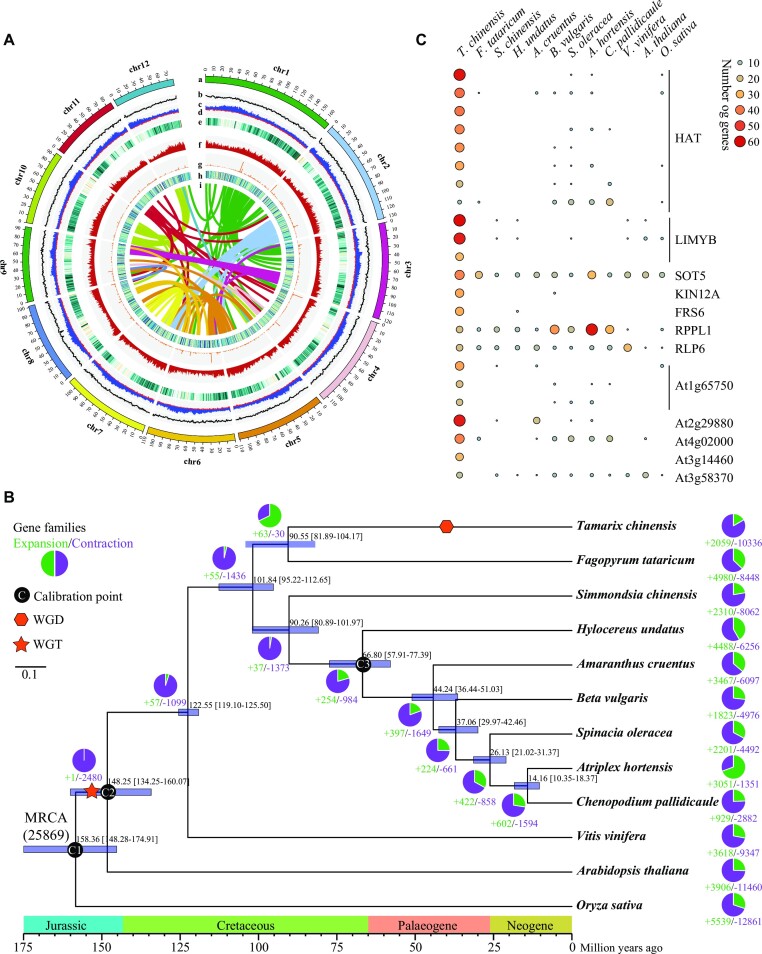
Genome evolution of *T. chinensis*. (A) Genomic features of *T. chinensis*. a, Circular representation of the pseudo-chromosomes. b, GC content. c, LTR/Gypsy distribution. d, LTR/Copia distribution. e, Repeat elements distribution. f, Protein-coding gene frequency. g, Distribution of noncoding RNAs. h, Distribution of log_2_ of gene expression levels. i, Intragenome collinear blocks. All distributions are displayed in a window size of 1 Mb. (B) Phylogenetic analysis of *T. chinensis* based on 959 one-to-one orthologous genes shared across 12 plant species, including 8 Caryophyllales species and 3 outgroups (*V. vinifera, A. thaliana*, and *O. sativa*) by RAxML using GTRCAT module with 200 bootstrap replicates. The pie chart represents the number of gene family expansions and contractions. The black dot indicates the calibration point. The star and hexagon indicate whole-genome triplication (WGT) and whole-genome duplication (WGD) events. The node label displays 95% highest probability density (HPD) of divergence ages. MRCA, most recent common ancestor. All the branches represent bootstrap values equal to 100, which are not shown in the figure. (C) Comparison of the number of genes among each significantly expanded gene family between *T. chinensis* and other examined plants.

PacBio and Hi-C data were integrated to construct a chromosome-level *T. chinensis* genome. First, by high-throughput sequencing, a total of 213.61 Gb (∼147× genome coverage) PacBio long reads and 138.67 Gb (∼95× genome coverage) Hi-C data were produced (Supplementary Table S1). Next, PacBio data were used for genome assembly, resulting in a preliminary body of around 1.32 Gb genome sequence containing 342 contigs with an N50 size of 11.93 Mb (Table 1). Finally, Hi-C data were used to assign the contigs to correct chromosomal positions, showing that more than 99.5% of the preliminary assembly was anchored to 12 pseudochromosomes (Fig. 1A, Supplementary Fig. S3, and Table 1). Collectively, these results showed that the final genome assembly is 1.32 Gb containing 63 super-scaffolds with an N50 value of 110.03 Mb.

**Table 1:** Features of *T. chinensis* genome assembly

Type	Parameter	Value
Assembly	Genome size (Gb)	1.324
	Chromosome-scale scaffolds (Gb)	1.317
	Total number of scaffolds	63
	Total number of chromosomes	12
	Scaffold N50 (Mb)	110.03
	Scaffold L50	6
	Total number of contigs	342
	Contig N50 (Mb)	11.93
	Contig L50	45
	GC content of the genome (%)	36.7
	Complete BUSCOs	97.4%
	Quality value (QV)	39.03
Annotation	Repeat sequences (Gb)	0.979 (73.94%)
	Total number of protein-coding genes	26,426
	Complete BUSCOs	93.9%
	Average length of genes (bp)	1,233.60
	Average exons per gene	5.30
	Annotated in Swiss-Port	15,422
	Annotated in NCBI NR	22,348
	Annotated in COG	21,796
	Annotated in InterPro	23,468
	Annotated in GO	14,064
	Annotated in KEGG	15,393

Four approaches were used to evaluate genome assembly quality. First, assembly quality was assessed using BUSCO, revealing that 1,571 of 1,614 (97.4%) BUSCOs were captured entirely in the genome assembly (Supplementary Fig. S4 and Supplementary Table S2). Next, Illumina and PacBio data were mapped to the genome assembly, revealing a high mapping rate of 99.84% (Illumina) and 94.31% (PacBio) and high coverage of 98.01% (Illumina) and 98.15% (PacBio), respectively. Then, transcripts assembled based on RNA-seq data were aligned to the assembly, showing that 85,077 of 90,812 (93.68%) transcripts were assigned to the genome assembly. Last, Merqury estimated the assembly base accuracy and completeness, resulting in a high QV of 39.03 (Table 1). The above results suggested that the assembled *T. chinensis* genome was high quality in genome completeness, baseline accuracy, and contiguity.

### Genome annotation

Genome annotation includes identifying repetitive elements and protein-coding genes. First, *de novo* and homology-based methods were used for identifying repetitive elements, resulting in around 0.98 Gb (74.24%) of repetitive elements in the genome sequence. Long terminal repeat–retrotransposons (LTR-RTs), which accounted for 45.52% of the whole genome sequence, were the most abundant elements (Supplementary Table S3). Of these LTR-RTs, *Gypsy/DIRS1* and *Ty1/Copia* were the most common families, accounting for 22.13% and 12.06% of the total genome sequence.

By integrating *ab initio*, transcript-based, and homology-based methods, 26,426 high-confident protein-coding genes were identified, which exhibited an average of 5.30 exons and a mean length of 1,233.60 bp (Table 1). The quality of gene predictions was assessed using BUSCO, revealing that 1,515 of 1,614 (93.9%) BUSCOs were captured entirely in predicted gene sets (Supplementary Table S4). Subsequently, predicted genes were functionally annotated by scanning multiple databases, resulting in 24,211 (91.62%) protein-coding genes exhibiting known functional annotations (Supplementary Fig. S5).

### Gene family evolution

To explore the evolutionary history of *T. chinensis*, a polygenetic tree based on 33 one-to-one orthologous genes shared across 32 angiosperm species, including 29 Caryophyllales species (5 with genome data and 27 with transcriptome data) and 3 outgroup species (Supplementary Table S5), showed that *T. chinensis* and *T. hispida* diverged from the most recent common ancestor (MRCA) of *T. ramosissima* c. 6.08 Ma ago, *Tamarix* split from the MRCA of *Reaumuria* c. 50.24 Ma ago, and Tamaricaceae diverged from the MRCA of Frankeniaceae c. 78.5 Ma ago (Supplementary Fig. S6).

Comparative genomics analysis was performed on 12 plant species with genome data, including 8 Caryophyllales species and 3 outgroups (*V. vinifera, A. thaliana*, and *O. sativa*) (Supplementary Table S6). A total of 25,869 orthogroups were identified among *T. chinensis* and other species (Supplementary Table S7). Of these orthogroups, 7,097 orthogroups were shared among all species examined, of which 959 orthogroups contained single-copy genes (Supplementary Fig. S7). A polygenetic tree was constructed to perform gene family expansion and contraction analysis, and the results showed that 2,059 expansions and 10,336 contractions were identified in *T. chinensis* (Fig. 1B). *P* values for each gene family were calculated, and 60 significant families (*P* < 0.05) were identified in *T. chinensis* (Supplementary Table S8), including 56 expansions and 4 contractions, which had larger expansions than in *F. tataricum* (47), *Simmondsia chinensis* (32), and *H. undatus* (26). Functional annotation of the expansions showed that 23 families had known functional annotations (Fig. 1C and Supplementary Table S9). Of these, 8 expansion gene families with 271 genes were annotated as *DAYSLEEPER* (*HAT*), which encodes a transposase-like protein playing essential roles in regulating plant growth and development [97, 98]. The second largest expansion gene families (3 of 23; 141 genes) were annotated as *L10-interacting MYB domain-containing protein* (*LIMYB*), a transcriptional repressor involved in plant antiviral immunity [99]. In addition, 2 significant expansions (46 genes) were annotated as putative disease resistance, such as the *putative disease resistance RPP13-like protein 1* (*RPPL1*). These results suggested that significantly expanded gene families likely contributed to the high environmental adaptation of *T. chinensis*.

### Whole-genome and tandem duplications associated with salinity adaptation in *T. chinensis*

**Figure 2:**
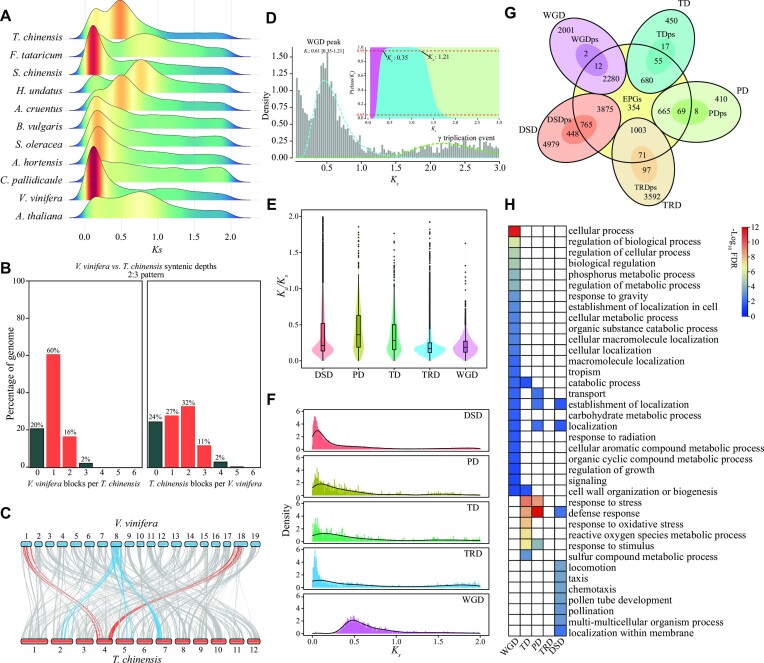
Whole-genome duplication event and gene duplications. (A) The synonymous substitution rates (*K_s_*) distribution for paralog gene pairs of *T. chinensis* and other plant species, including 8 Caryophyllales species and 2 outgroups (*V. vinifera* and *A. thaliana*). (B) The interspecies synthetic depths between *V. vinifera* and *T. chinensis*. (C) Macrosynteny between *V. vinifera* and *T. chinensis* karyotypes. The sky-blue line represents the 3 copies of *V. vinifera* syntenic blocks per *T. chinensis*. The red line indicates the 2 copies of *T. chinensis* syntenic blocks per *V. vinifera*. (D) *K_s_* distribution for paralog gene pairs from *T. chinensis* using WGD software. The *K_s_* distribution was subjected to the BGMM module in WGD for mixed-model fitting, resulting in the hypothesized WGD peaks. Afterward, the average and variance of each WGD peak were estimated, and the paralog gene pairs of each WGD peak with 95% probability were extracted. The blue dashed curve represents the WGD peak with *K_s_* ranging from 0.35 to 1.21 (mean 0.61). (E) The *K_a_*/*K_s_* ratios of the 5 types of duplications. DSD, dispersed duplications; PD, proximal duplications; TD, tandem duplications; TRD, transposed duplications. (F) The *K_s_* distribution of the 5 types of duplications. (G) Venn diagram shows the number of shared and specific gene duplications between the expanded genes (EPGs) and 5 types of duplications. DSDps, the dispersed duplications underwent positive selection; PDps, proximal duplications underwent positive selection; TDps, the tandem duplications underwent positive selection; TRDps, the transposed duplications underwent positive selection; WGDps, the WGD duplications underwent positive selection. (H) GO category enrichment analyses on the shared EPGs of 5 types of gene duplications.

*K_s_* distribution of each pairwise paralog gene and interspecies syntenic analyses were performed to identify putative WGD events in the evolutionary history of *T. chinensis*. Analysis of *K_s_* distributions revealed 2 distinct peaks in the *T. chinensis* genome (Fig. 2A). Interspecies syntenic analysis showed that *V. vinifera* and *T. chinensis* exhibited a 2:3 pattern for synthetic depth (Fig. 2B,C and Supplementary Fig. S8), suggesting a more recent WGD event occurred in *T. chinensis*. A fitting curve on *K_s_* distributions was performed. It showed that the WGD peak mainly ranged from 0.35 to 1.21 with a median of 0.61 (Fig. 2D), which was found to be shared by other species within Tamaricaceae (Supplementary Figs. S9–S10), indicating a Tamaricaceae-specific WGD event. Based on the time of divergence and mean peak *K_s_* values of orthologous genes of syntenic blocks between *T. chinensis* and *V. vinifera*, Tamaricaceae synonymous nucleotide substitutions rate was estimated to be 7.62 × 10^−9^ substitutions per site per year (Supplementary Fig. S11), resulting in an estimated time of the WGD event c. 39.88 ± 12.95 Ma ago in the middle of Palaeogene (Fig. 1B).

**Figure 3:**
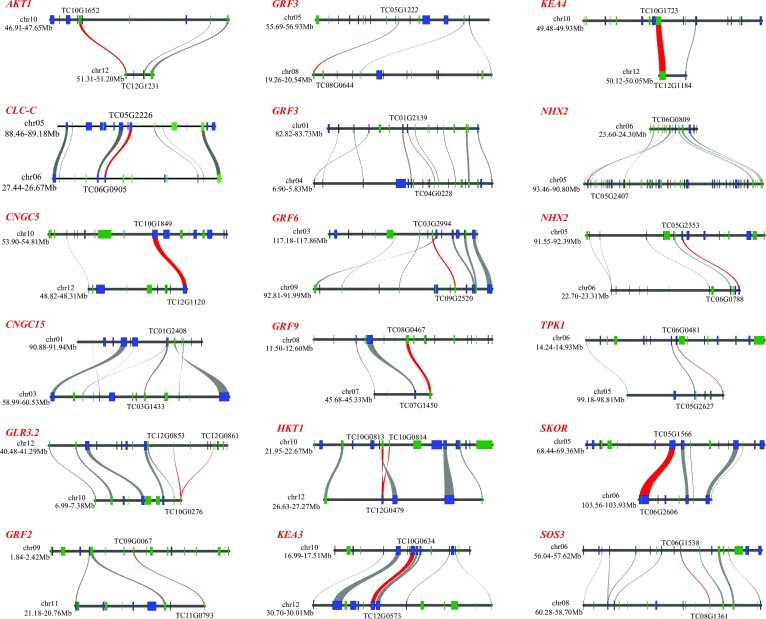
The syntenic relationships of the whole-genome duplications involved in salt stress sensing and ion homeostasis in *T. chinensis*. The red line represents the major gene pairs with the syntenic relationships. AKT1, potassium channel AKT1; CLC, chloride channel protein CLC; CNGC, cyclic nucleotide- and calmodulin-regulated ion channel; GLR, glutamate receptor (ligand-gated ion channel); GRF, 14-3-3–like protein (general regulatory factor); HKT, sodium transporter HKT; KEA, K^+^ efflux antiporter; NHX, sodium/hydrogen exchanger; SKOR, stelar K^+^ outward rectifying channel; TPK, two-pore potassium channel (calcium-activated outward-rectifying potassium channel).

To explore the differences in functions of gene duplications, a total of 19,935 duplications were identified and classified into 5 types: 4,281 WGD genes (21.47%), 1,130 tandem duplications (TDs, 5.67%), 1,075 proximal duplications (PDs, 5.39%), 4,595 transposed duplications (23.05%), and 8,854 dispersed duplications (DSDs, 44.41%) (Supplementary Table S10). The *K_a_*/*K_s_* ratios of the 5 types of duplications were calculated and revealed that PD and TD exhibited higher *K_a_*/*K_s_* scores than any other type (Fig. 2E), suggesting rapid sequence divergence and strong positive selection in PD and TD duplications. A comparison of the expanded genes (EPGs) and each duplication type showed that WGD and DSD duplications accounted for more than 69% (6,155 of 8,857) of total EPGs (Fig. 2G), suggesting a critical contributor to gene family expansions (Fig. 2A,F). Analysis of GO functional enrichment revealed different functions for the 5 duplications (Fig. 2H). For instance, WGD genes enriched GO terms implicated in the regulation of the biological and cellular process, cellular localization, and signaling, while TDs enriched GO categories involved in response to stress like oxidative stress and reactive oxygen species metabolic process. An essential process in plant response to salt stress is to sense and maintain ion homeostasis. There were 76 WGD duplications found to be involved in salt stress sensing and ion homeostasis (Fig. 3, Supplementary Fig. S12, and Supplementary Table S11). For example, genes encoding GLR3.2, CNGC5/15, AKT1, SKOR, TPK1, HKT1, NHX2, and SOS3 were present in the WGD type. Ten genes encoding 14-3-3–like proteins as molecular switches in plant tolerance to salinity stress [100] were also present in the WGD type. These results suggested that WGD and tandem duplications were primarily related to salinity adaptation in *T. chinensis*.

### Transcriptomic responses to salinity stress

**Figure 4:**
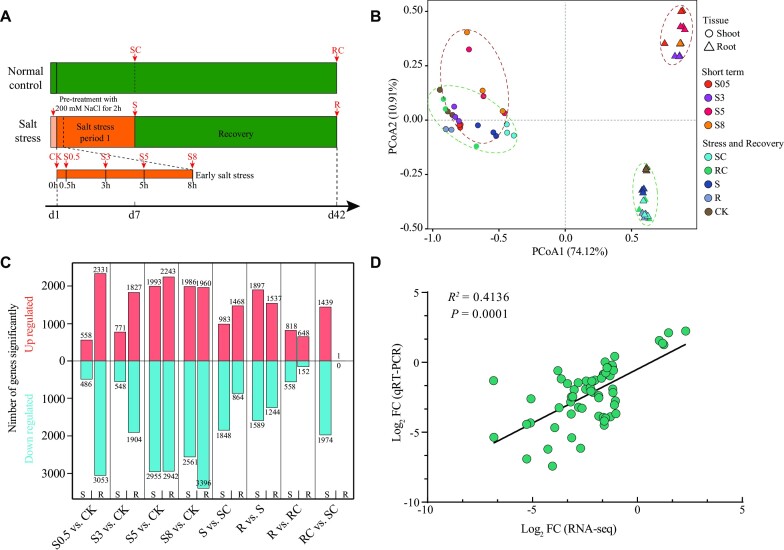
Experimental setup and transcriptome analysis. (A) Overview of RNA-seq experimental design. Before imposing NaCl stress, the cutting clones were pretreated in a medium containing 200 mM NaCl for 2 hours to avoid salt shock. The cutting clones were grown for 7 days on a hydroponic medium with 300 mM NaCl before transferring to the hydroponic medium without NaCl for 35 days to recovery. Seven time points for sample collection were selected to cover early salt stress (including 300 mM NaCl stressed 0.5, 3, 5, and 8 hours) and late salt stress and recovery (including 300 mM NaCl stressed 7 days and 35 days of recovery). Subsequently, 54 samples covering the 7 time points with 3 biological replicates per condition were harvested. (B) Principal coordinates analysis of gene expressions revealed that root and shoot showed distinct gene expression profiles; peculiarly, the early salt stress and late stress and recovery treatments on roots also exhibited distinct gene expression profiles. (C) Statistics of the differentially expressed genes were generated from 4 differential analysis methods, including DESeq2, edgeR, ROTS, and Limma. (D) The Pearson correlation coefficient between the qRT-PCR and RNA-seq results. The analysis was conducted using GraphPad Prism 9.

A transcriptomic experiment was conducted to better understand the molecular mechanisms underlying high adaptation to salt stress of *T. chinensis*. The cutting clones were hydroponically grown for 7 days on a hydroponic medium with 300 mM NaCl before being transferred to a hydroponic medium without NaCl for 35 days to recover (Fig. 4A). Seven time points spanning early salt stress, late salt stress, and recovery were selected. A total of 54 RNA-seq libraries covering the 7 time points with 3 biological replicates per time point were generated and sequenced, producing more than 1.18 billion paired-end reads (Supplementary Table S12). On average, each sample generated more than 21 million reads, of which more than 94% mapped to the *T. chinensis* genome assembly. Principal coordinates analysis of gene expressions revealed that root and shoot showed distinct gene expression profiles; peculiarly, the early salt stress, late stress, and recovery treatments on root also exhibited distinct gene expression profiles (Fig. 4B). Integration of 4 differential analysis methods, including DESeq2, edgeR, ROTS, and Limma, was performed to strengthen the identification of DEGs, generating 7,118 and 6,023 DEGs in root and shoot during early salt exposure, respectively (Fig. 4C and Supplementary Tables S13–S15). Of those DEGs, there were 86 EPGs, 1,653 WGD genes, and 631 TD genes in the root and 77 EPGs, 1,433 WGD genes, and 506 TD genes in the shoot. Meanwhile, 3,675 and 6,272 DEGs were identified in root and shoot during late salt stress and recovery, respectively (Fig. 4C and Supplementary Tables S13, S16, S17). Among the DEGs, there were 51 EPGs, 852 WGD genes, and 431 TD genes in the root and 1,489 EPGs, 1,433 WGD genes, and 595 TD genes in the shoot. A randomly selected 27 DEGs were verified by qRT-PCR (Supplementary Table S18), resulting in a significant and positive correlation between transcriptome results and qRT-PCR data, indicating the transcriptome data are reliable (Fig. 4D).

### Identifying hub genes responding to early salt stress

**Figure 5:**
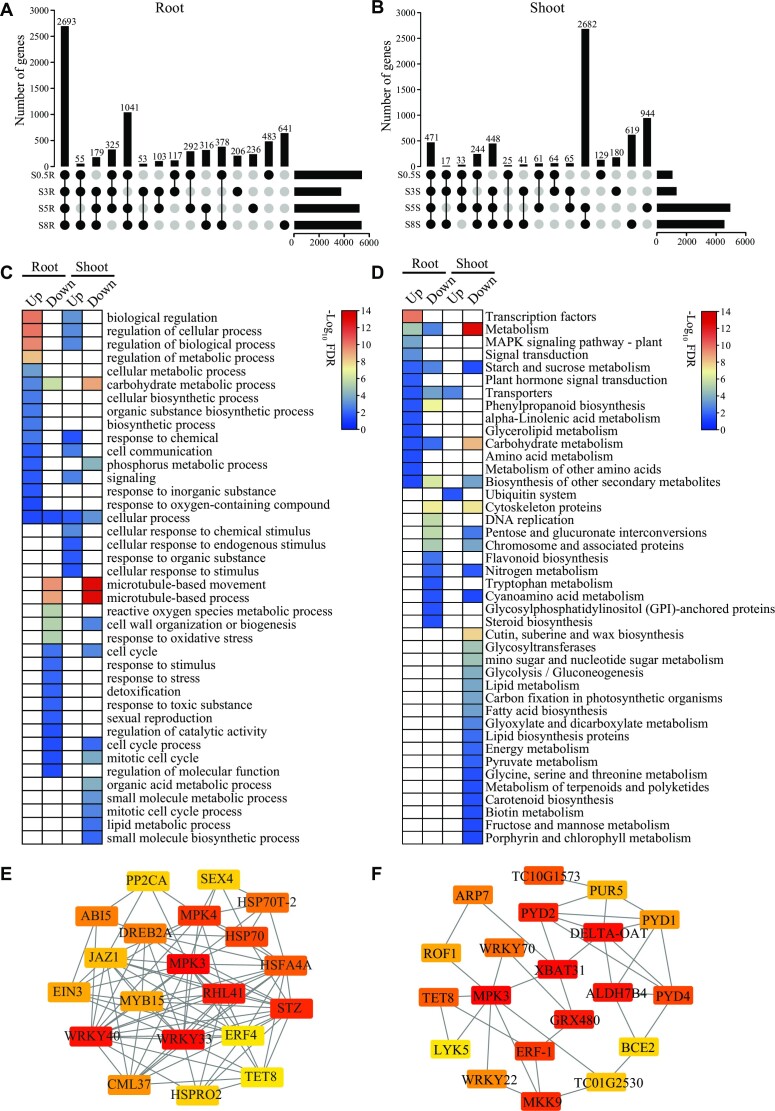
Identification of hub genes responding to early salt stress. (A, B) Upset plots of the number of DEGs identified in the root (A) and shoot (B) during early salt stress. (C) GO category enrichment analyses on the shared upregulated or downregulated DEGs among the time points of salt exposure in the root (0.5, 3, 5, and 8 hours of salt exposure) and shoot (5 and 8 hours of salt exposure). (D) KEGG enrichment analyses were performed on the shared upregulated or downregulated DEGs among the time points in the root and shoot during early salt exposure. (E, F) The hub-ranked genes were identified in the root (E) and shoot (F). Nodes colored from red to yellow represent degree ranking.

The above-identified DEGs were further dissected to identify putative hub genes that respond to salinity stress during early salt stress. First, intersection analysis of DEGs (7,118) at 0.5, 3, 5, and 8 hours of salt exposure in the root showed that more than 37% (2,693 of 7,118) of DEGs were shared among the 4 time points (Fig. 5A). By analyzing the DEGs (6,023) identified in the shoot, it revealed a distinct 2-phase pattern with more than 44% (2,682 of 6,023) of DEGs shared by the second time points from 5 to 8 hours (Fig. 5B), suggesting a delayed response to salt stress in the shoot after salt exposure. Of the common root DEGs, 1,219 and 1,473 were respectively upregulated and downregulated at 0.5, 3, 5, and 8 hours of salt exposure (Supplementary Fig. S13A and Supplementary Table S19). Among those DEGs, 11 EPGs, 329 WGD genes, and 130 TD genes exhibited upregulated expression, and 20 EPGs, 328 WGD genes, and 149 TD genes exhibited downregulated expression at 0.5, 3, 5, and 8 hours of salt exposure. For example, the *LIMYB* (*TC02G0567*) gene showed the largest difference with a 169.76- to 256.70-fold increase among the differentially expressed EPGs at 0.5, 3, 5, and 8 hours of salt exposure. Nine WRKY transcription factor coding genes (e.g., *WRKY33* and *WRKY75*) and several genes (e.g., *CBL4/SOS3, NHX2, AKT1*, and *CHX20*) involved in stress sensing and ion homeostasis showed upregulation at the 4 time points. In the shoot, 961 and 1,720 common DEGs were upregulated and downregulated at 5 and 8 hours of salt exposure, respectively (Supplementary Fig. S13B and Supplementary Table S20). Among the DEGs, 11 EPGs, 214 WGD genes, and 85 TD genes exhibited upregulated expression, and 12 EPGs, 450 WGD genes, and 111 TD genes exhibited downregulated expression at 5 and 8 hours of salt exposure. For instance, WGD genes associated with stress sensing like *CBL10* and *CSC1* (a Ca^2+^-permeable channel coding gene) and ion transport like *CLC-C* and *potassium transporter 10* showed upregulated expressions at the 2 time points.

GO category enrichment analyses showed that upregulated and downregulated DEGs exhibited different functions in root and shoot (Fig. 5C). Specifically, upregulated DEGs in both root and shoot enriched GO terms for cellular and biological process regulation, as well as response to chemical, signaling, and cellular communication. Downregulated DEGs in both root and shoot enriched GO categories for microtubule-based process, cell wall organization or biogenesis, and cell cycle. The downregulated DEGs in the root specifically enriched GO terms for response to oxidative stress and detoxification. Analysis of KEGG functional enrichment showed that upregulated DEGs in the root specifically enriched transcription factors, MAPK signaling pathway, signal transduction, and plant hormone signal transduction (Fig. 5D), whereas shoot specifically enriched the term for the ubiquitin system. In addition, DEGs downregulated in the root and shoot primarily enriched terms for metabolisms such as carbohydrate metabolism, nitrogen metabolism, and energy metabolism (specifically in the shoot).

Next, shared upregulated DEGs in both root and shoot were focused and used to identify hub genes. In the root, a PPI network with 1,711 interactions consisting of 534 nodes and 1,711 edges was constructed (Supplementary Fig. S14 and Supplementary Table S21). Based on the degree of a node, 20 hub genes were identified in the root, with *WRKY33, WRKY40, MPK3, MPK4*, and *RHL41* being the most ranked genes (Fig. 5E). In the shoot, a PPI network was constructed with 689 interactions consisting of 400 nodes and 689 edges (Supplementary Fig. S15 and Supplementary Table S22). A subnetwork containing 20 hub genes was identified, of which *MPK3, XBAT31, DELTA-OAT, GRX480*, and *ALDA7B4* were the most ranked genes (Fig. 5F). These results suggested that the identified hub genes were likely to play an essential role in responding to salt stress during early exposure.

### Identifying hub genes that respond to late salt stress and recovery

The effectiveness of the recovery mechanism following severe environmental conditions is crucial for plant survival. To understand the underlying recovery mechanisms of *T. chinensis* after salt stress, we performed a comparative transcriptome analysis on root and shoot tissues of cutting clones that were salt treated for 7 days (S/SC) and recovered with a hydroponic medium for up to 35 days (R/RC) (Fig. 4A).

**Figure 6:**
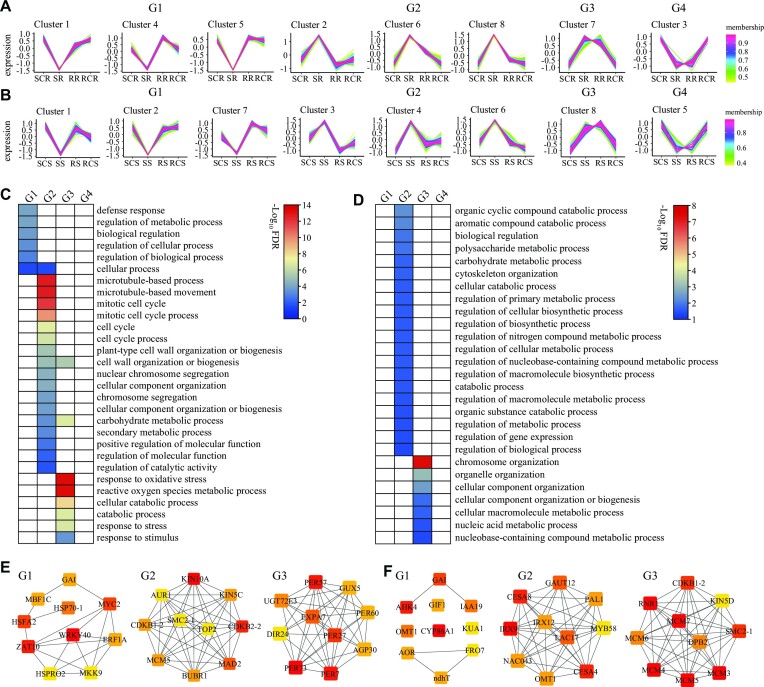
Identification of hub genes that respond to late salt stress and recovery. (A) Gene dynamics analysis of 1,705 DEGs with opposite trends between stress and recovery or maintained at the recovery stage in the root. (B) Gene dynamics analysis of 612 DEGs with opposite trends between stress and recovery or maintained at the recovery stage in the shoot. (C, D) GO category enrichment analyses on the DEGs in the 4 major groups in the root (C) and shoot (D). (E, F) The hub-ranked genes were identified in the root (E) and shoot (F) groups. Nodes colored from red to yellow represent degree ranking.

In the root, a total of 2,332 (1,468 upregulated and 864 downregulated), 2,781 (1,537 upregulated and 1,244 downregulated), and 800 (648 upregulated and 152 downregulated) DEGs were identified in S vs. SC, R vs. S, and R vs. RC, respectively (Fig. 4C and Supplementary Table S13), whereas only 1 upregulated DEG was identified in RC vs. SC. GO terms enrichment analyses on the DEGs of each comparison revealed that most of the salt-induced genes recovered at stage R (Supplementary Fig. S16A). An intersection analysis of the upregulated and downregulated DEGs in each comparison identified 1,705 DEGs with opposite trends between stress and recovery or maintained at the recovery stage, which included 20 EPGs (e.g., *LIMYB* and *RPPL1*), 386 WGD genes (e.g., *CSC1, HKT1, TPK1*, and *CHX20*), and 198 TD genes (e.g., 12 class III peroxidase coding genes, including *PER26, PER52, PER56, PER57*, and *PER60*) (Supplementary Fig. S17A,C and Supplementary Table S23). Gene dynamics analyses on the DEGs identified 8 clusters, which were further classified into 4 groups according to gene expression trends (Fig. 6A). Clusters 1, 4, and 5 were grouped into G1, consisting of 557 DEGs exhibiting downregulation at stage S and then recovered at stage R. Clusters 2, 6, and 8 were grouped into G2, consisting of 856 DEGs exhibiting upregulation at stage S and then recovered at stage R. Cluster 7 (G3) and cluster 3 (G4) comprised 255 and 37 DEGs exhibiting maintained upregulation or downregulation at both stages S and R, respectively. GO category enrichment analysis revealed a distinct difference in gene functions (Fig. 6C): G1 enriched genes involved in defense response and biological and cellular process regulation; G2 enriched genes related to microtubule-based process, cell cycle, cell wall organization or biogenesis, and secondary metabolic process; and G3 gathered genes participated in carbohydrate metabolic process, response to oxidative stress, response to stress, and cellular catabolic process. PPI network analyses of the DEGs in each group identified 3 networks (Supplementary Fig. S17A and Supplementary Tables S24–S26): G1 had a network with 448 interactions composed of 71 nodes and 170 edges, G2 had a network exhibiting 3,691 interactions consisting of 135 nodes and 2,555 edges, and G3 had a network with 169 interactions composed of 30 nodes and 98 edges. Based on the degree of a node, a subnetwork consisting of 10 hub genes was identified in each group, with *WRKY40* and *ZAT10* being the most ranked central genes in G1, *KIN10A* and *CDKB2-2* being the most ranked in G2, and *PER7, PER27, PER57*, and *PER73* the most ranked hub genes in G3 (Fig. 6E).

In the shoot, a total of 2,831 (983 upregulated and 1,843 downregulated), 3,486 (1,897 upregulated and 1,589 downregulated), 1,376 (818 upregulated and 558 downregulated), and 3,413 (1,439 upregulated and 1,974 downregulated) DEGs were identified in S vs. SC, R vs. S, R vs. RC, and RC vs. SC, respectively (Fig. 4C and Supplementary Table S13). GO category enrichment analyses of the DEGs of each comparison revealed various terms related to metabolic process, biological and cellular process regulation, and response to stimulus (Supplementary Fig. S16B). Similar to the root, a total of 612 DEGs were identified, showing opposite trends between stages S and R or maintained at stage R, which included 16 EPGs (e.g., *HAT* and *RPPL1*), 154 WGD genes (e.g., *GLR3.2* and *Ca^2+^/H^+^ antiporter CAX1/3*), and 73 TD genes (e.g., *PER72, GSTT1*, and *GSTU8*) (Supplementary Fig. S17B,D and Supplementary Table S27). Gene expression dynamics analyses showed that DEGs were grouped into 8 clusters, which were further classified into 4 major groups (Fig. 6B). Clusters 1, 2, and 7 were grouped into G1 consisting of 288 downregulated DEGs at stage S and then recovered at stage R, while clusters 3, 4, and 6 were grouped into G2 consisting of 212 upregulated DEGs at stage S and then recovered at stage R. Cluster 8 (G3) and cluster 5 (G4) consisted of 78 and 34 DEGs, representing maintained upregulated or downregulated at both stages S and R, respectively. GO category analyses on the DEGs in each group showed that G2 enriched various terms mainly related to the regulation of biological, cellular catabolic, and metabolic processes, whereas G3 enriched genes involved in the chromosome, organelle, and cellular component organization and macromolecule metabolic process (Fig. 6D). PPI network analyses for each group of DEGs identified 3 networks (Supplementary Fig. S18B and Supplementary Tables S28–S30). Among the networks, G1 had 117 interactions comprising 46 nodes and 48 edges, G2 had 160 interactions with 28 nodes and 100 edges, and G3 exhibited 168 interactions with 28 nodes and 165 edges. Subsequently, a subnetwork consisting of 10 central genes was identified for each group according to the degree of a node (Fig. 6F). Among the subnetworks, *CYP86A1* and *AHK4* were the most ranked hub genes in G1; *IRX9, CESA4*, and *CESA8* were the most ranked central genes in G2; and *MCM3, MCM4, MCM5, MCM7*, and *RNR1* were the most ranked hub genes in G3. These results suggested that the central genes identified in root and shoot probably played an essential role in responding to salt stress during late salt stress and recovery.

## Discussion

This work described a chromosome-level genome for *T. chinensis*, a pioneer tree species of the coastal wetland ecosystem in northern China. We found that the families of *HAT* and *LIMYB* genes were significantly expanded in the *T. chinensis* genome, of which *HAT* is essential for plant growth and development [97, 98], and *LIMYB* as a transcriptional repressor functioned in plant antiviral immunity [99], likely suggestive of the critical roles in high environmental adaptation. We dated a WGD event in Tamaricaceae lineage c. 39.88 ± 12.95 Ma in the middle of Palaeogene. It is suggested that the WGD event was shared between *Tamarix* and *Reaumuria*, as previously suggested by dense phylogenomic sampling across Caryophyllales [101]. WGD and TD duplications are critical drivers in plant adaptive evolution to enhance high tolerance to environmental stress [36, 102–106]. We found that WGD and TD in *T. chinensis* contributed gene duplications involved in salt stress sensing, ion homeostasis, response to stress like oxidative stress, and reactive oxygen species metabolic process, suggestive of significant contributors in high-salinity adaptation of *T. chinensis*.

During early salt stress, we found that more than 37% of DEGs were shared among the 4 time points in the root. In contrast, the shoot exhibited a distinct 2-phase pattern, with more than 44% of DEGs shared only by time points from 5 to 8 hours, suggesting a delayed response to salt stress in the shoot rather than root after salt exposure. *WRKY* transcription factors are crucial regulators of a plant responding to salinity stress [107, 108]. For example, *WRKY33* is a vital transcriptional regulator involved in multiple regulatory networks to promote plant salt tolerance [109–112]. In *Pyrus betulaefolia, WRKY40* positively regulates a V-type-H^+^-ATPase gene to promote salt tolerance and organic acid accumulation [113]. In *Fortunella crassifolia, WRKY40* positively regulates *salt overly sensitive 2* (*SOS2*) and *Δ-1-pyrroline-5-carboxylate synthetase 1* (*P5CS1*) homologs to enhance salt tolerance [114]. We found that *WRKY33* and *WRKY40* transcription factors were the most ranked hub genes in the root during early salt stress, indicating their essential roles in enhancing *T. chinensis* tolerance. MAPK cascade is an essential pathway that regulates plants’ responses to multiple environmental stresses [115]. For example, *MPK3*, a positive regulator, regulates the lipid transfer protein AZI1 to improve stress resistance in *Arabidopsis* [116, 117]. *MPK3/6* is a negative regulator degrading several *Arabidopsis* response regulators to enhance salt tolerance [118]. The OsMKK1–OsMPK4 signaling pathway regulates salt resistance in rice [119]. This study identified *MPK3* and *MPK4* as the most ranked hub genes during early salt stress, suggesting the critical roles in *T. chinensis* against salt stress. We also found several pivotal hub genes in plants responding to various environmental stresses. For instance, *XBAT31*, one of the most ranked central genes in the shoot, is an E3 ligase that responds to warm temperatures by mediating ELF3 (a thermosensor) degradation in *Arabidopsis* [120]. *DELTA-OAT*, another central gene in the shoot, encodes an ornithine-delta-aminotransferase essential for resistance to nonhost disease in *Arabidopsis* [121]. These results suggest that the identified hub genes played an essential role in responding to salt stress during early exposure and may be critical gene resources used for salt-tolerant plant genetic improvement.

This study identified several hub genes related to plant recovery after salt stress. We found that *WRKY40, ZAT10, KIN10A*, and *CDKB2-2* were the most ranked hub genes associated with stress recovery in the root, of which the first 2 genes were downregulated at the stress stage, while the last 2 were upregulated at the stress stage. *WRKY40*, as a key regulator of salt-responsive genes, is shared between early salt stress and late stress recovery but has opposite expression patterns, suggesting a dual regulatory role in plant response to salt stress [122]. *ZAT10*, a zinc-finger transcription factor, exhibited dual roles in promoting plant salt tolerance [123] and cadmium uptake and detoxification [124]. In the shoot, *CYP86A1* and *AHK4* hub genes were downregulated, while *IRX9, CESA4*, and *CESA8* were upregulated at the stress stage. Among these genes, *AHK4*, encoding a histidine kinase, is a cytokinin receptor sensing environmental signals that function as negative regulators in response to osmotic stress [125, 126]. The hub genes *IRX9, CESA4*, and *CESA8* were associated with plant secondary cell wall formation [127–129], suggesting functional adaptation of secondary wall genes under abiotic stress [130]. We found that *PER7, PER27, PER57*, and *PER73* were the most ranked hub genes in root, which encode class III peroxidases that functioned as an antioxidant for biotic or abiotic stress resistance in plants [131], suggesting their essential roles in adapting to salt stress. While in the shoot, *MCM3, MCM4, MCM5*, and *MCM7* were the most ranked central genes, which are the components of the minichromosome maintenance complex, and played crucial roles in DNA replication initiation and cell division [132], suggesting that cell division was likely associated with stress response [133].

In summary, this study first described the nearly complete reference genome of halophyte *T. chinensis*. Gene families related to plant growth and development have significantly expanded in *T. chinensis*. Whole-genome and tandem duplications contributed to the expansion of genes involved in salinity adaptation in *T. chinensis*. Several hub genes were identified as responding to salt stress in *T. chinensis*, but more validation experiments were needed. Therefore, this study will be a valuable genetic resource for investigating the evolutionary adaptation of tamarisk and the genetic improvement of plant salt tolerance.

## Supplementary Material

giad053_GIGA-D-23-00079_Original_Submission

giad053_GIGA-D-23-00079_Revision_1

giad053_Response_to_Reviewer_Comments_Original_Submission

giad053_Reviewer_1_Report_Original_Submission Showkat Ganie -- 4/10/2023 Reviewed

giad053_Reviewer_1_Report_Revision_1 Showkat Ganie -- 6/19/2023 Reviewed

giad053_Reviewer_2_Report_Original_Submission Caroline Belser -- 4/21/2023 Reviewed

giad053_Supplemental_Files

## Data Availability

The genome sequencing data, including PacBio long reads, Illumina short reads, and Hi-C data, are available via NCBI with BioProject accession PRJNA855314. The RNA sequencing data are deposited in the NCBI under accession PRJNA855335. This Whole Genome Shotgun project has been deposited at DDBJ/ENA/GenBank under the accession JANKMZ000000000. The version described in this article is JANKMZ010000000. Additional supporting data, also including BUSCO and Merqury results, are available via the *GigaScience* database GigaDB [134].

## Additional files

**Supplementary Table S1**. The statistics of PacBio long reads and Hi-C data.

**Supplementary Table S2**. The quality of genome assembly evaluated by BUSCO.

**Supplementary Table S3**. Identification of repetitive elements in the genome assembly.

**Supplementary Table S4**. The quality of gene predictions evaluated by BUSCO.

**Supplementary Table S5**. Detailed data used for phylogenetic analysis.

**Supplementary Table S6**. The species used for comparative genomic analysis.

**Supplementary Table S7**. Statistics of orthogroups per species.

**Supplementary Table S8**. Statistics of gene family expansions and contractions among 12 examined plant species.

**Supplementary Table S9**. Function annotation of the significant gene family expansions.

**Supplementary Table S10**. Statistics of gene duplications.

**Supplementary Table S11**. The WGD duplications involved in salt stress sensing and ion homeostasis balance.

**Supplementary Table S12**. Statistics of RNA sequencing data.

**Supplementary Table S13**. Statistics of differentially expressed genes identified by four differential analysis methods, including DESeq2, edgeR, ROTS, and Limma.

**Supplementary Table S14**. The differentially expressed genes identified in the root during early salt stress.

**Supplementary Table S15**. The differentially expressed genes identified in the shoot during early salt stress.

**Supplementary Table S16**. The differentially expressed genes identified in the root during late salt stress and recovery.

**Supplementary Table S17**. The differentially expressed genes identified in the shoot during late salt stress and recovery.

**Supplementary Table S18**. The PCR primers used in the present study.

**Supplementary Table S19**. The differentially expressed genes identified in the root shared among 0.5, 3, 5, and 8 h salt exposure.

**Supplementary Table S20**. The differentially expressed genes identified in the shoot shared by 5 and 8 h salt exposure.

**Supplementary Table S21**. Protein-protein interactions regarding the shared upregulated differentially expressed genes identified in the root.

**Supplementary Table S22**. Protein-protein interactions regarding the shared upregulated differentially expressed genes identified in the shoot.

**Supplementary Table S23**. The differentially expressed genes identified with opposite trends between stress and recovery or maintained at the recovery stage in the root during late salt stress and recovery.

**Supplementary Table S24**. Protein-protein interactions of the G1 genes identified in the root.

**Supplementary Table S25**. Protein-protein interactions of the G2 genes identified in the root.

**Supplementary Table S26**. Protein-protein interactions of the G3 genes identified in the root.

**Supplementary Table S27**. The differentially expressed genes identified with opposite trends between stress and recovery or maintained at the recovery stage in the shoot during late salt stress and recovery.

**Supplementary Table S28**. Protein-protein interactions of the G1 genes identified in the shoot.

**Supplementary Table S29**. Protein-protein interactions of the G2 genes identified in the shoot.

**Supplementary Table S30**. Protein-protein interactions of the G3 genes identified in the shoot.

**Supplementary Fig. S1**. The photo of the diploid *T. chinensis* Lour. ‘Lucheng No.1’ (2n = 24) and the salt glands of *T. chinensis* Lour. ‘Lucheng No.1’ under salt stress.

**Supplementary Fig. S2**. Genome size estimation of *T. chinensis* ‘Lucheng No.1’. (a) The size of *T. chinensis* ‘Lucheng No.1’ genome was estimated by 30-mer frequency analysis, resulting in a depth of 22 for the highest peak. The size of the *T. chinensis* ‘Lucheng No.1’ genome was determined by genome size = number of k-mer /average k-mer depth. (b) The size of *T. chinensis* ‘Lucheng No.1’ was evaluated using flow cytometry by comparing it to the genome size of *Z. mays* ‘B73’ of approximately 2.32 Gb.

**Supplementary Fig. S3**. The Hi-C contact maps of *T. chinensis* genome assembly. The maps were visualized using HiCPlotter.

**Supplementary Fig. S4**. Bar chart showing the quality of genome assembly assessed by BUSCO.

**Supplementary Fig. S5**. Upset plots of the number of functionally annotated genes in the *T. chinensis* genome.

**Supplementary Fig. S6**. Phylogenetic analysis of *T. chinensis* based on 33 one-to-one orthologous genes shared across 32 angiosperm species using the modified phylome approach. The number above each branch represents the bootstrap value (a bootstrap value equal to 100 is not showing). The black dot indicates the calibration point.

**Supplementary Fig. S7**. Venn diagram shows the number of shared and species-specific gene families among *T. chinensis* and other 11 plants.

**Supplementary Fig. S8**. Dot plot displays the comparative analysis of *T. chinensis* and *V. vinifera* genomes. The red and blue squares indicate some main duplication events, and the dots exhibit synteny gene pairs.

**Supplementary Fig. S9**. The 4DTv (fourfold degenerate synonymous sites) distribution of Tamaricaceae species.

**Supplementary Fig. S10**. Ks (synonymous substitution rate) distribution for paralog gene pairs in five Tamaricaceae species, including *T. ramosissima, T. hispida, R. kaschgarica, R. soongarica*, and *R. trigyna*.

**Supplementary Fig. S11**. Ks (synonymous substitution rate) distribution for *T. chinensis* ortholog genes with *V. vinifera*. Based on divergence time (115 Ma ago) and mean peak Ks values (1.754) of orthologous genes of syntenic blocks between *T. chinensis* and *V. vinifera*, the synonymous nucleotide substitutions rate of Tamaricaceae was estimated to be 7.62×10^-9^ substitutions per site per year. According to divergence date = Ks / 2r, the age of *T. chinensis* WGDs was calculated.

**Supplementary Fig. S12**. The syntenic relationships of the whole-genome duplications involved in salt stress sensing and ion homeostasis balance in *T. chinensis*. The red line represents the major gene pairs with the syntenic relationships.

**Supplementary Fig. S13**. Heatmap showing the expression of differentially expressed genes identified in the root (a) and shoot (b) responding to early salt stress.

**Supplementary Fig. S14**. A protein-protein interaction network containing 1711 interactions composed of 534 nodes and 1711 edges was constructed in the root exposed to early salt stress. Nodes colored red and yellow represent the hub genes. Color from red to yellow represents degree ranking.

**Supplementary Fig. S15**. A protein-protein interaction network containing 689 interactions composed of 400 nodes and 689 edges was constructed in the shoot exposed to short-term stress. Nodes colored red and yellow represent the hub genes. Color from red to yellow represents degree ranking.

**Supplementary Fig. S16**. Gene ontology category enrichment analysis of the upregulated and downregulated DEGs of each comparison in the root (a) and shoot (b) during salt stress and recovery.

**Supplementary Fig. S17**. Identify differentially expressed genes responding to late salt stress and recovery. (a, b) Upset plots of the number of upregulated and downregulated DEGs of each comparison identified in the root (a) and shoot (b) during salt stress and recovery. (c, d) Heatmap showing the expression of differentially expressed genes identified in the root (c) and shoot (d) that respond to responding to late salt stress and recovery.

**Supplementary Fig. S18**. Protein-protein interaction network of the DEGs of each dynamic group was constructed in the root (a) and shoot (b) during salt stress and recovery. Nodes colored red and yellow represent the hub genes, and color from red to yellow represents degree ranking.

## Abbreviations

bp: base pair; BUSCO: Benchmarking Universal Single-Copy Orthologs; CBL: calcineurin B-like protein; CDPK: calcium-dependent protein kinase; CLC: chloride channel protein; CNGC: cyclic nucleotide-gated ion channel; DEG: differentially expressed gene; DSD: dispersed duplication; EPG: expanded gene; Gb: gigabase; GLR: glutamate receptor; GO: Gene Ontology; kb: kilobase; KEGG: Kyoto Encyclopedia of Genes and Genomes; LTR-RT: long terminal repeat–retrotransposon; MAPK: mitogen-activated protein kinase; Mbp: megabase pair; MRCA: most recent common ancestor NCBI: The National Center for Biotechnology Information; PD: proximal duplication; PPI: protein–protein interaction; qRT-PCR: quantitative real-time reverse-transcription polymerase chain reaction; ROS: reactive oxygen species; TD: tandem duplication; TRD: transposed duplication; WGD: whole-genome duplication.
