# Supplementary material for: Genomic analyses provide insights into the evolution and salinity adaptation of halophyte Tamarix chinensis
Source: Gigascience. 2023 Jul 26;12:giad053. doi: 10.1093/gigascience/giad053 (PMC10370455; doi:10.1093/gigascience/giad053)
Supplement: giad053_GIGA-D-23-00079_Revision_1 [file giad053_giga-d-23-00079_revision_1.pdf]

GigaScience

Genomic analyses provide insights into the evolution and salinity adaptation of Tamarix chinensis

--Manuscript Draft--

**Manuscript Number:**	GIGA-D-23-00079R1
**Full Title:**	Genomic analyses provide insights into the evolution and salinity adaptation of Tamarix chinensis
**Article Type:**	Research
**Funding Information:**	the Improved Variety Program of Shandong Province of China (2019LZGC009) — Dr. Ke Qiang Yang
**Abstract:**	Background: The woody halophyteTamarix chinensis is a pioneer tree species in the coastal wetland ecosystem of Northern China, exhibiting high resistance to salt stress.However, the genetic information underlying salt tolerance in T. chinensis remains to be seen. Here we present a genomic investigation of T. chinensis to elucidate the underlying mechanism of its high resistance to salinity.<br>Results: Using a combination of PacBio and high-throughput chromosome conformation capture data, a chromosome-level T. chinensis genomewas assembled with a size of 1.32 Gb and scaffold N50 of 110.03 Mb. Genome evolution analyses revealed that T. chinensis significantly expanded families of HAT and LIMYBgenes. Whole-genome and tandem duplications contributed to the expansion of genes associated with the salinity adaptation of T. chinensis. Transcriptome analyses on root and shoot tissues during salt stress and recovery were performed, and several hub genes responding to salt stress and identified. WRKY33/40, MPK3/4, and XBAT31 were critical in responding to salt stress during early exposure, while WRKY40, ZAT10, AHK4, IRX9, and CESA4/8 were involved in responding to salt stress during late stress and recovery. PER7/27/57/73 encoding class III peroxidase and MCM3/4/5/7 encoding DNA replication licensing factor may contribute to stress memory maintenance.<br>Conclusions: The results presented here reveal the genetic mechanisms underlying salt adaptation in T. chinensis, thus providing important genetic resources for evolutionary studies on tamarisk and plant salt tolerance genetic improvement.
**Corresponding Author:**	Jian Ning Liu, Ph.D.<br>Shandong Agricultural University<br>Tai'an, Shandong CHINA
**Corresponding Author Secondary Information:**	
**Corresponding Author's Institution:**	Shandong Agricultural University
**Corresponding Author's Secondary Institution:**	
**First Author:**	Jian Ning Liu
**First Author Secondary Information:**	
**Order of Authors:**	Jian Ning Liu
	Hongcheng Fang
	Qiang Liang
	Yuhui Dong
	Changxi Wang
	Liping Yan
	Xinmei Ma
	Rui Zhou
	Xinya Lang

Powered by Editorial Manager® and ProduXion Manager® from Aries Systems Corporation

	Shasha Gai
	Lichang Wang
	Shengyi Xu
	Ke Qiang Yang
	Dejun Wu
**Order of Authors Secondary Information:**	
**Response to Reviewers:**	Dear Dr. Hans Zauner,<br>Thank you very much for your useful comments and suggestion on our manuscript entitled "Genomic analyses provide insights into the evolution and salinity adaptation of Tamarix chinensis" (GIGA-D-23-00079).<br><br>We have carefully revised the manuscript according to reviewer's comments. The point-by-point responses are below, and the revision parts in the revised manuscript have been marked in red.<br><br>We wish the revised version to be considered for publication in GigaScience.<br><br>Thanks again for your kind help.<br><br>Sincerely yours,<br>Ke Qiang Yang<br><br><br>Response to Reviewer 1 Comments<br><br>Point 1: Have authors compared these duplication events in T. chinensis with a glycophyte counterpart of T. chinensis, in order to get an idea how these duplications have evolved T. chinensis genome adapted to high salinity? Sorry, but I don't have any idea about the 5 species in the clade with which authors have compared it.<br>Response 1: Thanks for your question. We did not compare these duplication events in T. chinensis with a glycophyte counterpart of T. chinensis because we have yet to get a proper glycophyte counterpart of T. chinensis.<br>In this study, we selected 26 species in the NCORE subclade and five other species, including Beta vulgaris, Spinacia oleracea, Vitis vinifera, Solanum lycopersicum, and Oryza sativa, as the outgroup to perform all vs. all homology searches and phylogenetic orthology inference. Then, we selected five other Tamaricaceae halophyte species [1,2] belonging to the NCORE subclade with T. chinensis, including 2 Tamarix species (Tamarix ramosissima and T. hispida) and 3 Reaumuria species (Reaumuria trigyna, R. soongarica, and R. kaschgarica) to compare 4DTv (fourfold degenerate synonymous sites) and Ks (synonymous substitution rate) distributions to confirm a Tamaricaceae-specific WGD event occurred in T. chinensis. Furthermore, as you mentioned in point 3, we have analyzed the expression of WGD-mediated duplicated genes under early salt stress, late stress, and recovery treatments, suggesting several WGD-mediated duplicated genes like WRKY transcription factor coding genes (e.g., WRKY33 and WRKY75) and several genes (e.g., CBL4/SOS3, CBL10, CSC1, NHX2, AKT1, CLC-C, and CHX20) involved in stress sensing and ion homeostasis likely have evolved T. chinensis genome adapted to high salinity.<br><br>References<br>[1] Santos J, Al-Azzawi M, Aronson J, Flowers TJ. eHALOPH a database of salt-tolerant plants: helping put halophytes to work. Plant Cell Physiol. 2016;57(1):e10. doi: 10.1093/pcp/pcv155.<br>[2] Zhao, K., Song, J., Feng, G., Zhao, M., Liu, J. Species, types, distribution, and economic potential of halophytes in China. Plant Soil. 2011;342:495–509. Doi:10.1007/s11104-010-0470-7.<br><br>Point 2: In this connection, authors should also analyses a publicly available RNA-seq data-set(s) of a T. chinesis glycophyte counterpart and compare the temporal expression of hub genes (representing early, late and recovery periods of salt treatment) to see how differently T. chinensis genes behave, which could reveal its signature expression and also if this is the level of expression or time of expression as

Powered by Editorial Manager® and ProduXion Manager® from Aries Systems Corporation

the plants strategy that makes it more tolerant.
Response 2: Thanks for your excellent suggestion. The research on the salt tolerance mechanism of T. chinesis is weak, and publicly available proper RNA-seq data-set(s) of T. chinesis and its glycophyte counterpart are few; therefore, we have yet to find any data-set(s) for comparative transcriptome analyses to see how differently T. chinensis genes behave.

Point 3: Although authors have validated the transcriptomic data by analyzing the expression of 27 random genes, I suggest authors analyses the expression of a few genes from the ones which have expanded in T. chinensis (LIMYB genes or WGD or TD-mediated duplicated genes) and which authors think might be associated with its salt tolerance, to see how their behavior is under salt stress and that if they have a functional significance.
Response 3: Thanks for your constructive suggestions. According to your suggestions, we have analyzed the expression of a few genes from those that have expanded in T. chinensis (LIMYB genes or WGD or TD-mediated duplicated genes) under salt stress, and the relevant description was added in the revised manuscript (Line 384-391, Line 403-418, Line 452-456, Line 481-485):

Line 384-391: "Integration of four differential analysis methods, including DESeq2, edgeR, ROTS, and Limma, was performed to strengthen the identification of DEGs, generating 7118 and 6023 DEGs in root and shoot during early salt exposure, respectively (Fig. 4c; Table S13-15). Of those DEGs, there were 86 EPGs, 1653 WGD genes, 631 TD genes in the root; 77 EPGs, 1433 WGD genes, and 506 TD genes in the shoot. Meanwhile, 3675 and 6272 DEGs were identified in root and shoot during late salt stress and recovery, respectively (Fig. 4c; Table S13, Table S16-17). Among the DEGs, there were 51 EPGs, 852 WGD genes, and 431 TD genes in the root; 1489 EPGs, 1433 WGD genes, and 595 TD genes in the shoot."
Line 403-418: "Of the common root DEGs, 1219 and 1473 were respectively upregulated and downregulated at 0.5, 3, 5, and 8 h salt exposure (Fig. S13a, Table S19). Among those DEGs, 11 EPGs, 329 WGD genes, and 130 TD genes exhibited upregulated expression, and 20 EPGs, 328 WGD genes, and 149 TD genes exhibited downregulated expression at 0.5, 3, 5, and 8 h salt exposure. For example, LIMYB (TC02G0567) gene showed the largest difference with a 169.76 - 256.70-fold increase among the differentially expressed EPGs at 0.5, 3, 5, and 8 h salt exposure. Nine WRKY transcription factor coding genes (e.g., WRKY33 and WRKY75) and several genes (e.g., CBL4/SOS3, NHX2, AKT1, and CHX20) involved in stress sensing and ion homeostasis showed upregulated at the four-time points. In the shoot, 961 and 1720 common DEGs were upregulated and downregulated at 5 and 8 h salt exposure, respectively (Fig. S13b, Table S20). Among the DEGs, 11 EPGs, 214 WGD genes, and 85 TD genes exhibited upregulated expression, and 12 EPGs, 450 WGD genes, and 111 TD genes exhibited downregulated expression at 5 and 8 h salt exposure. For instance, WGD genes associated with stress sensing like CBL10 and CSC1 (a Ca2+-permeable channel coding gene) and ion transport like CLC-C and potassium transporter 10 showed upregulated expressions at the two-time points."
Line 452-456: "An intersection analysis of the upregulated and downregulated DEGs in each comparison identified 1705 DEGs with opposite trends between stress and recovery or maintained at the recovery stage, which included 20 EPGs (e.g., LIMYB and RPPL1), 386 WGD genes (e.g., CSC1, HKT1, TPK1, and CHX20), and 198 TD genes (e.g., 12 class III peroxidase coding genes, including PER26, PER52, PER56, PER57, and PER60) (Fig. S17a, c, Table S23)."
Line 481-485: "Similar to the root, a total of 612 DEGs were identified, showing opposite trends between stages S and R or maintained at stage R, which included 16 EPGs (e.g., HAT and RPPL1), 154 WGD genes (e.g., GLR3.2 and Ca2+/H+ antiporter CAX1/3), and 73 TD genes (e.g., PER72, GSTT1, and GSTU8) (Fig. S17b, d, Table S27)."

Point 4: Line 254: Why did authors choose to use Arabidopsis for protein BLAST but not populus which is the model for trees?
Response 4: Thanks for your question. Compared to Populus, Arabidopsis has the most abundant protein-protein interaction (PPI) information, with most PPIs that are experimentally determined, so we choose to use Arabidopsis for protein BLAST. The relevant description has been added in the revised manuscript (Line 253-256): "Among plant species, Arabidopsis has the most abundant protein-protein interaction (PPI) data

Powered by Editorial Manager® and ProduXion Manager® from Aries Systems Corporation

	available, and most PPIs have been experimentally determined, so the protein sequences from the DEGs were BLAST-searched against the A. thaliana protein database for homolog identification."<br><br>Point 5: Authors report that genes that showed maintained up/downregulation during salt stress and recovery stages could be the 'memory genes' while the genes with opposite expression between salt stress and recovery stages are not. I don't agree with this statement.<br>Although I agree that the former could be the memory genes because of their constant up/downregulation from stress to recovery phase. However, how do authors know that the expression of these genes does not dampen with time, even after 35 days? To ensure they are memory genes, authors should impose a second salt stress after recovery and see how the expression levels of these genes change. Moreover, the genes in the latter category (opposite expression between salt stress and recovery stages) can be 'memory genes' as well. We know that first stress causes epigenetic changes at memory loci and these loci get imprinted during the recovery which upon recurrent stress give rise to a modified response. So, pls revisit this conclusion based on a second salt stress.<br>Response 5: Thanks for your excellent comments. The description of memory genes is inaccurate, and the revised manuscript has corrected the relevant description.<br><br>Point 6: If T. chinensis remains/grows constantly in the coastal wetland ecosystem, then what is the physiological/ecological significance of these potentially salt memory genes for this plant, when the stress is constant and not recurring.? Pls elaborate.<br>Response 6: Thanks for your excellent comment. It is a crucial evolutionary ecology topic that needs to be elaborated on based on extensive provenances of the tree species from different habitats. The genomic and salt stress transcriptomic data were obtained only from the Tamarix chinensis cultivar ‘Lucheng No.1’ in our manuscript, which cannot support this topic. Your suggestion has provided us with a good idea for our future research. Thank you again.<br><br>Point 7: Lastly, to make the title catchier and more discoverable, add halophyte in the title as: Genomic analyses provide insights into the evolution and salinity adaptation of recretohalophyte Tamarix chinensis.<br>Response 7: Thanks for your suggestion. We agree to add "halophyte" in the title: “Genomic analyses provide insights into the evolution and salinity adaptation of halophyte Tamarix chinensis” in the revised manuscript. Tamarix chinensis exhibits multiple pathways responding to salt stress. It was found that root and shoot showed distinct gene expression profiles to salt stress in this manuscript. Meanwhile, more scientific evidence is needed for the mechanism of salt secretion in the scale leaf and shoot of Tamarix chinensis.<br><br>Point 8: Line 37: should be "…salt stress were identified".<br>Response 8: Following your suggestion. We have changed "…salt stress and identified" to "…salt stress were identified" in the revised manuscript (Line 37).<br><br>Point 9: Line 43: it is better to write 'genomic resources' than genetic resources.<br>Response 9: Thanks for your suggestion. In the revised manuscript, we have changed "genetic resources" to "genomic resources" (Line 44).<br><br>Point 10: Line 81-82: "…which the plant has various applications such as environmental governance and landscape value". Pls rewrite this bit.<br>Response 10: Thanks for your comment. We have removed the relevant description in the revised manuscript.<br><br>Point 11: Line 84: should be "In addition, because of its high tolerance to salt…".<br>Response 11: Following your suggestion, we have changed the description "In addition, as its high tolerance to salt…" to "In addition, because of its high tolerance to salt…" in the revised manuscript (Line 82).<br><br>Point 12: Line 89: "Dissecting the whole genome of plants with high salt tolerance" should be "Dissecting the whole genome of plants for different salt tolerance genomic resources…".<br>Response 12: Thanks for your suggestion. We have changed the description

"Dissecting the whole genome of plants with high salt tolerance" to "Dissecting the whole genome of plants for different salt-tolerance genomic resources…" in the revised manuscript (Line 86).

Point 13: Line 94: better to write genomic resources.
Response 13: Following your suggestion. We have changed "genetic resources" to "genomic resources" in the revised manuscript (Line 92).

Point 14: Line 377 and 383: should be "…early salt stress, late salt stress, and recovery…".
Response 14: Thanks for your suggestion. In the revised manuscript, we have changed the description "…early salt stress, and late stress and recovery…" to "…early salt stress, late salt stress, and recovery…" (Line 377 and 383).

Point 15: Line 387-388: add ", respectively" after "and recovery".
Response 15: Following your suggestion, we have added ", respectively" after "and recovery" in the revised manuscript (Line 389).

Point 16: Line 387, 395, 412, 418 and so on: pls don't use "at roots, at shoots". Instead use "in roots or in shoots". Correct all such things in the MS.
Response 16: Thanks for your suggestion. We have changed the description "at roots, at shoots" to "in roots, in shoots" throughout the text in the revised manuscript.

Point 17: Line 400: should be "Of the common root DEGs, 1219 and 1473 were respectively upregulated and downregulated at 0.5, 3, 5, and 8 h of salt exposure (Table...".
Response 17: Following your suggestion, we have changed the description in the revised manuscript (Line 403-404).

Point 18: Line 416: "in response to salt stress exposure to early salt stress" doesn't make any sense.
Response 18: Thanks for your suggestion. We have deleted the description "...involved in response to salt stress exposure to early salt stress" in the revised manuscript.

Response to Reviewer 2 Comments
Point 1: I would have liked to have a summary figure with the expression of the genes according to the salinity conditions. This could have helped in understanding.
Response 1: Thanks for your suggestion. In this study, we have provided Fig. 5e and f to show the hub genes identified in the root (Fig. 5e) and shoot (Fig. 5f) responding to early salt stress, and Fig. 6e and f to show the hub genes identified in the root (Fig. 5e) and shoot (Fig. 5f) that respond to late salt stress and recovery in T. chinensis. Following your suggestion, in order to show the expression of genes according to the salinity conditions more intuitively, we added Fig. S13 and Fig. S17c, d to show the expression of differentially expressed genes identified in the root and shoot responding to early salt stress (Fig. S13) and late salt stress and recovery (Fig. S17c, d).

Point 2: I appreciated the effort to conduct qPCR experiments to verify the DEGs identification. But I'm not sure that the authors can speak about strong correlation coefficient (R^2=0.41).
Response 2: Thanks for your comment. The relative description has been rephrased as "A randomly selected 27 DEGs were verified by qRT-PCR (Table S18), resulting in a significant and positive correlation between transcriptome results and qRT-PCR data, indicating the transcriptome data are reliable (Fig. 4d)." in the revised manuscript (Line 392-394).

Point 3: Did the authors check the presence of telomeric repeats at the extremities of the biggest scaffolds?
Response 3: Thanks for your question. We checked the presence of telomeric repeats at the extremities of 12 pseudochromosomes in the T. chinensis assembly using tidk v. 0.2.31 (https://github.com/tolkit/telomeric-identifier) with the find command and the following parameters: "-w 10000 -c Caryophyllales", showing that pseudochromosome 1, 6, 9, 10, and 12 respectively with only one end matched the known telomeric repeat for Caryophyllales clade. We agree that checking the presence of telomeric repeats at the extremities of chromosomal sequences is a necessary evaluation indicator for the

Powered by Editorial Manager® and ProduXion Manager® from Aries Systems Corporation

	telomere-to-telomere (T2T) genome assembly. Because of the non-T2T genome sequencing and assembly strategy used in our study, the relevant results of this evaluation were not present in this manuscript.<br><br>Point 4: The merqury score is commonly used to estimate the quality of the consensus (https://genomebiology.biomedcentral.com/articles/10.1186/s13059-020-02134-9).<br>Response 4: Thanks for your suggestion. We performed genome assembly quality evaluation using Merqury with k=25 based on Illumina short-reads, and the relative description has been added in the revised manuscript (Line 163-164 and Line 288-291):<br>Line 163-164: "Finally, Merqury v. 1.3 [54] with the parameter: k=25 estimated the assembly quality value (QV)."<br>Line 288-291: "At last, Merqury estimated the assembly base accuracy and completeness, resulting in a high QV of 39.03 (Table 1). The above results suggested that the assembled T. chinensis genome was high quality in genome completeness, baseline accuracy, and contiguity."<br><br>Point 5: Line 156: « All software was executed under the parameters. » which parameters?<br>Response 5: Thanks for your question. All software was executed with default parameters, and the relative description was added in the revised manuscript (Line 154).
**Additional Information:**	
**Question**	**Response**
Are you submitting this manuscript to a special series or article collection?	No
**Experimental design and statistics**<br><br>Full details of the experimental design and statistical methods used should be given in the Methods section, as detailed in our Minimum Standards Reporting Checklist. Information essential to interpreting the data presented should be made available in the figure legends.<br><br>Have you included all the information requested in your manuscript?	Yes
**Resources**<br><br>A description of all resources used, including antibodies, cell lines, animals and software tools, with enough information to allow them to be uniquely identified, should be included in the Methods section. Authors are strongly encouraged to cite Research Resource Identifiers (RRIDs) for antibodies, model organisms and tools, where possible.	Yes

Have you included the information requested as detailed in our Minimum Standards Reporting Checklist?	
**Availability of data and materials**<br><br>All datasets and code on which the conclusions of the paper rely must be either included in your submission or deposited in publicly available repositories (where available and ethically appropriate), referencing such data using a unique identifier in the references and in the "Availability of Data and Materials" section of your manuscript.<br><br>Have you have met the above requirement as detailed in our Minimum Standards Reporting Checklist?	Yes

Powered by Editorial Manager® and ProduXion Manager® from Aries Systems Corporation

Manuscript Click here to access/download;Manuscript;Tamarisk_forpublication.docx

Genomic analyses provide insights into the evolution and salinity adaptation of halophyte *Tamarix chinensis*

Jian Ning Liu[1], Hongcheng Fang[1,2,3], Qiang Liang[1,2,3], Yuhui Dong[1,2,3], Changxi Wang[1], Liping Yan[4], Xinmei Ma[1], Rui Zhou[1], Xinya Lang[1], Shasha Gai[1], Lichang Wang[1], Shengyi Xu[1], Ke Qiang Yang[1,2,3 *], Dejun Wu[4 *]

[1]College of Forestry, Shandong Agricultural University, Taian 271018, China

[2]State Forestry and Grassland Administration Key Laboratory of Silviculture in the Downstream Areas of the Yellow River, Shandong Agricultural University, Taian 271018, China

[3]Shandong Taishan Forest Ecosystem Research Station, Shandong Agricultural University, Taian 271018, China

[4]Shandong Provincial Academy of Forestry, Jinan 250014, China

ORCID iDs: Jian Ning Liu [0000-0002-8932-054X]; Hongcheng Fang [0000-0003-0624-624X]; Qiang Liang; Yuhui Dong; Changxi Wang; Liping Yan; Xinmei Ma; Rui Zhou; Xinya Lang; Shasha Gai; Lichang Wang; Shengyi Xu; Ke Qiang Yang [0000-0003-0022-0620]; Dejun Wu;

[*]Corresponding author:

Ke Qiang Yang E-mail: yangwere@126.com; Dejun Wu E-mail: sdlky412@163.com

Abstract

Background: The woody halophyte *Tamarix chinensis* is a pioneer tree species in the coastal wetland ecosystem of Northern China, exhibiting high resistance to salt stress. However, the genetic information underlying salt tolerance in *T. chinensis* remains to be seen. Here we present a genomic investigation of *T. chinensis* to elucidate the underlying mechanism of its high resistance to salinity.

Results: Using a combination of PacBio and high-throughput chromosome conformation capture data, a chromosome-level *T. chinensis* genome was assembled with a size of 1.32 Gb and scaffold N50 of 110.03 Mb. Genome evolution analyses revealed that *T. chinensis* significantly expanded families of *HAT* and *LIMYB* genes. Whole-genome and tandem duplications contributed to the expansion of genes associated with the salinity adaptation of *T. chinensis*. Transcriptome analyses were performed on root and shoot tissues during salt stress and recovery, and several hub genes responding to salt stress were identified. *WRKY33/40, MPK3/4*, and *XBAT31* were critical in responding to salt stress during early exposure, while *WRKY40, ZAT10, AHK4, IRX9*, and *CESA4/8* were involved in responding to salt stress during late stress and recovery. In addition, *PER7/27/57/73* encoding class III peroxidase and *MCM3/4/5/7* encoding DNA replication licensing factor maintained up/downregulation during salt stress and recovery stages.

Conclusions: The results presented here reveal the genetic mechanisms underlying salt adaptation in *T. chinensis*, thus providing important genomic resources for evolutionary studies on tamarisk and plant salt tolerance genetic improvement.

Keywords: *Tamarix chinensis*, genome assembly, genome evolution, transcriptome, salinity

adaptation

Introduction

It is reported that around 7% of the global land and approximately one-third of global irrigated lands have become salt-affected, and the salinity soils seriously limit plant growth and crop production [1]. Salt stress, one of the most detrimental environmental stressors, mainly causes osmotic stress and ionic toxicity in plants [2, 3]. To cope with adverse effects, plants adapt to various mechanisms, including activating the osmotic stress pathway, regulating ion homeostasis, and mediating hormone signaling, resulting in metabolic and physiological responses [4-6]. After exposure to salt stress, plants first sense signals through multiple receptors or sensors, such as Ca^{2+}-permeable channel glutamate receptor (GLR) [7] and cyclic nucleotide-gated ion channel (CNGC) for Na^+ permeation [8]. Then elevated cellular Ca^{2+} induced reactive oxygen species (ROS) and activation of several signal molecules, including 14-3-3-like proteins, calcineurin B-like proteins (CBLs), calcium-dependent protein kinases (CDPKs), and calcineurin B-like interacting protein kinases (CIPKs) [4]. Subsequently, ROS-activated mitogen-activated protein kinases (MAPKs), in combination with the activated signal molecules, initiated several transcription factors like WRKYs, resulting in the transcription of multiple stress-responsive genes [9-11]. Furthermore, several ion carriers or channels played essential roles in ion homeostasis, such as potassium channel AKT1 [12], stelar K^+ outward rectifying channel (SKOR) [13], calcium-activated outward-rectifying potassium channel 1 (TPK1) [14], sodium transporter HKT1 [15], sodium/hydrogen exchangers (NHXs) [16], cation/H^+ antiporters (CHXs) [17], and chloride channel proteins (CLCs) [18]. Recent evidence demonstrated that salt stress could inhibit the cell cycle by controlling cell cycle regulators [19]. However, our understanding of the mechanisms underlying plant salt resistance is still on the way.

Halophytes, distinct from glycophytes, which represent most salt-sensitive plants, exhibit high salt tolerance and can survive in soils with high salt concentrations (> 200 mM NaCl) [20, 21]. Therefore, it is vital to understand the genomic information and mechanisms underlying their tolerance to salt stress, which may help to exploit and utilize these resources to cope with increasing saline soils. *Tamarix* (Tamaricaceae, Caryophyllales) is an Old-World genus containing approximately 90 species, grown widely in arid and semi-arid areas of Eurasia, Africa, the ancient Mediterranean Sea, and northwestern China [22, 23]. Among *Tamarix* species, the woody halophyte *Tamarix chinensis* Lour (saltcedar or tamarisk; NCBI:txid189791), a deciduous shrub or tree, is a pioneer species of coastal saline wetland ecosystem in Northern China and is a major component of the circumlittoral shelter forest known as the coastguard [24-26]. In addition, because of its high tolerance to salt stress and rapid growth, *T. chinensis* has been considered an ideal model for investigating plant salt tolerance mechanisms [27-29]. However, the scarcity of reference genome sequences in *T. chinensis* largely hampers a better understanding of the underlying mechanisms of its high salinity adaptation.

Dissecting the whole genome of plants for different salt-tolerance genomic resources is a pivotal approach to investigating plant adaptation mechanisms to salt stress. With rapid advances in high-throughput genome sequencing, increasing numbers of salt-tolerant plant genomes have been dissected, and their molecular adaptation to salinity environments has been clarified [30-36]. Here, we present a genomic investigation of *T. chinensis* to elucidate the underlying mechanism of its high resistance to salinity. This study will provide important genomics resources for evolutionary studies on tamarisk and the genetic improvement of plant salt tolerance.

Materials and methods

Plant material

Diploid *T. chinensis* Lour. 'Lucheng No.1' (2n = 24) (Fig. S1) was conserved in the Forestry Experimental Station of Shandong Agricultural University, Taian, China (117.15 E, 36.17 N). Total DNA and RNA were isolated from the healthy, tender shoots according to previously described methods [37]. Approximately 5 g of fresh and tender shoots were fixed with 1% formaldehyde and then used to extract intact nuclei to construct the high-throughput chromosome conformation capture (Hi-C) library as previously described [37, 38].

Genome survey

Flow cytometry analysis evaluated the size of *T. chinensis* 'Lucheng No.1' genome by comparing it to the genome size of *Zea mays* 'B73' (an internal reference, approximately 2.32 Gb) [39]. Fresh and tender shoots collected immediately were subjected to nuclei extraction and DNA staining by a Sysmex CyStain PI Absolute P kit according to the manufacturer's recommended protocols. The nuclear size was determined by a Sysmex CyFlow Cube6 flow cytometer (Sysmex, Lincolnshire, IL, USA) with at least 10,000 nuclei counts analyzed per plant. Flow cytometer output was analyzed using FlowJo v. 10.5.3 (BD Biosciences, San Diego, CA, USA).

Genome size of *T. chinensis* 'Lucheng No.1' was assessed using a k-mer method [40] based on Illumina short-reads. The DNA library with a 300 bp insert size was constructed and sequenced on an Illumina NovaSeq6000 platform (KeGene, Shandong, China) with a PE-150 module, yielding around 44.63 Gb of raw-data bases. After trimming by Trimmomatic (RRID:SCR_011848) v. 0.38 [41], around 44.23 Gb of validated bases were generated. A 30-mer frequency analysis was performed using Jellyfish (RRID:SCR_005491) v. 2.3.0 [40], resulting

in a depth of 22 for the highest peak. *T. chinensis* genome size was determined by genome size = number of k-mer /average k-mer depth.

Genome sequencing

A 40-kb insert size SMRT-bell library was constructed by SMRTbell Express Template Prep Kit 2.0 and sequenced on PacBio Sequel II (Pacific Biosciences) using Chemistry 2.0 for 15 h per SMRT Cell 8M. The process produced more than 213 Gb of subread bases, including more than 7.41 million subreads with an average length of 28.81 kb.

Two Hi-C libraries were prepared according to the *in situ* Hi-C library preparation protocol for plants [42]. Briefly, cross-linked nuclear chromatin was first treated with DpnII restriction enzyme (New England Biolabs). Next, nuclear chromatin was incorporated with biotin-14-dATP for end-repair, ligation, and DNA purification. The recovered ligations were sequenced on the Illumina NovaSeq6000 platform (KeGene, Shandong, China), yielding approximately 132.9 Gb high-quality bases.

The RNA-seq library was prepared by the TruSeq RNA Sample Preparation Kit (Illumina) and sequenced on the Illumina NovaSeq6000 instrument, generating around 9.85 Gb bases for subsequent gene prediction.

Genome assembly

The PacBio subreads were corrected, trimmed, and assembled using Canu (RRID:SCR_015880) v. 2.1 [43] under parameters: correctedErrorRate=0.045 and minReadLength=2000, resulting in a primary assembly of 2.16 Gb size with 5299 contigs exhibiting an N50 size of 4.43 Mb. The preliminary assembly was subjected to Purge_Dups (RRID:SCR_021173) v. 1.0.1 pipeline [44] to remove the duplications and obtain the purged primary sequence. The process yielded a

genome assembly comprising 342 contigs covering 1.32 Gb, represented by a contig N50 length of approximately 11.93 Mbp. The purged primary genome assembly was first polished by GCpp v. 2.0.2 (Pacific Biosciences) using PacBio subreads and subsequently polished by Pilon (RRID:SCR_014731) v. 1.23 [45] using Illumina short reads. The polished genome assembly was subjected to Genome assembly quality assessment, BUSCO (RRID:SCR_015008) v. 5.0.0 [46] with the embryophyta_odb10 dataset to assess assembly completeness, showing 97.1% BUSCOs being captured entirely in the genome sequence.

As previously described tools [37], Hi-C data were used for chromosomal-level assembly. In brief, using Juicer pipeline v. 1.6 [47], Hi-C data were aligned to the polished assembly to produce duplicate free contact maps. Subsequently, Hi-C maps were subjected to 3D-DNA pipeline v. 201013 [48] to construct chromosomal-level genome assembly. The resulting assembly was imported into Juicebox v. 2.13.07 [49] for final assembly manual review and refinement. The Hi-C contact maps for final assembly were visualized using HiCPlotter v. 0.6.6 [50]. All software was executed with default parameters.

Genome assembly quality assessment

Four approaches evaluated genome assembly quality: BUSCO, DNA, RNA sequencing data analysis, and Merqury quality statistics. First, BUSCO evaluated the completeness of the final assembly with the embryophyta_odb10 dataset. Next, Illumina and PacBio data were aligned to the assembly using Bowtie2 v. 2.4.2 [51] and pbmm2 v. 1.4.0 (Pacific Biosciences), and mapping rates were calculated. Then, RNA-seq data were subjected to Trinity v. 2.11.0 [52] to obtain full-length transcripts that were subsequently mapped to the assembly by BLAT v. 35 [53] to calculate genome mapping rates. Finally, Merqury (RRID:SCR_022964) v. 1.3 [54] with the parameter: k = 25 estimated the assembly quality value (QV).

Genome annotation

EDTA (RRID:SCR_022063) v. 2.0.0 [55] and RepeatMasker (RRID:SCR_012954)v. 4.07 [56] were integrated to identify repeat elements. MAKER (RRID:SCR_005309) pipeline v. 3.01.03 [57] was used to predict protein-coding genes. First, the Trinity assembled transcripts were subjected to PASA v. 2.4.1 [58] to generate high-quality transcripts, which were then used to train *ab initio* gene predictors, including SNAP (RRID:SCR_007936) [59], GENEMARK (RRID:SCR_011930) v. 4.68 [60], and AUGUSTUS (RRID:SCR_008417) v. 3.3.3 [61]. Subsequently, coding evidence from *ab initio* gene predictors was integrated using the MAKER pipeline, resulting in a comprehensive set of protein-coding genes. To improve gene annotation, the resulting gene models with an AED score < 0.2 were selected and imported into SNAP, GENEMARK, and AUGUSTUS programs for the second round of data re-training. The homology gene models were predicted using Exonerate v. 2.2.0 [62] by mapping the protein sequences of *Beta vulgaris* [63], *Spinacia oleracea* [64] *Vitis vinifera* [65], *Arabidopsis thaliana* [66], *Solanum lycopersicum* [67], *Populus trichocarpa* [68], and *Oryza sativa* [69] to the assembly. Finally, PASA transcripts, homology gene models, and re-trained gene models were imported into the MAKER program to obtain the final protein-coding genes.

The function of the predicted genes was annotated using InterProScan (RRID:SCR_005829) v. 5.48-83.0 [70] by searching against InterPro database v. 83.0 [71]. In addition, predicted genes were functionally annotated by scanning the non-redundant protein (nr) of NCBI and SwissProt databases using blastp v. 2.10.1 with parameters: E-value < 1e–5, coverage ≥ 50%, and identity ≥ 30%. Kyoto Encyclopedia of Genes and Genomes (KEGG) annotation was performed using KofamScan [72] with default parameters.

Phylogenetic analyses

According to the previous phylogenetic study, Caryophyllales can be divided into five subclades: PHYT, PORT, AMAR, CARY, and NCORE, with *T. chinensis* belonging to the NCORE subclade [73]. Therefore, we selected 26 species in the NCORE subclade and five other species, including *B. vulgaris*, *S. oleracea*, *V. vinifera*, *S. lycopersicum*, and *O. sativa*, as the outgroup to perform all *vs.* all homology searches and orthology inference from coding sequences. The phylogenetic orthology inference was made using a modified phylome approach [74].

Comparative genomics analysis was performed on 12 plant species with genomic data, including eight Caryophyllales species and three outgroups (*V. vinifera*, *A. thaliana*, and *O. sativa*). OrthoFinder (RRID:SCR_017118) v. 2.5.4 [75] made phylogenetic orthology inference. One-to-one orthologous genes were subjected to MAFFT (RRID:SCR_011811) v. 7.471 [76] for multiple sequence alignment. A phylogenetic tree was constructed by RAxML (RRID:SCR_006086) v. 8.2.12 [77] using the GTRCAT module with 200 bootstrap replicates. Species divergence time was inferred using PAML (RRID:SCR_014932) v. 4.9j [78]. For the first phylogenetic tree, four secondary calibration time points were set, including 14 - 56 Ma ago between *Rumex palustris* and *Rheum nobile*, 24 - 53 Ma ago between *B. vulgaris* and *S. oleracea*, 25 - 94 Ma ago within *Persicaria virginiana* branch, and 148 - 173 Ma ago within root node. Three secondary calibration time points were set for the second phylogenetic tree, including 53.4 - 78.9 within the *Hylocereus undatus* branch, 109 - 123.5 Ma ago between *A. thaliana* and *V. vinifera*, and 148 - 173 Ma ago in the root node. The time of divergence between species was retrieved from the TimeTree database [79]. Analysis of gene family expansion and contraction was performed by CAFÉ v. 5.0 [80] with parameters: lambda -s -p 0.05.

Whole-genome duplication events inference

We used synonymous substitution rates (K_s) distribution of paralog gene pairs and inter-species syntenic relationships to identify putative WGD events in the evolutionary history of *T. chinensis*. The K_s distribution of paralog gene pairs was analyzed using WGD v. 1.1 [81]. The K_s distribution was subjected to the BGMM module in WGD for mixed model fitting, resulting in putative WGD peaks. In addition, the DupGen_finder pipeline was used to classify gene duplications [82] with default parameters. The number of substitutions per nonsynonymous site (K_a), K_s, and K_a/K_s scores between each paralog gene pair was calculated by KaKs_Calculator v. 2.0 [83] with the YN model after constructing a codon alignment by PAL2NAL v. 14.0 [84]. For inter-species collinearity analysis between *T. chinensis* and *V. vinifera*, the top 10 hits of each protein from all *vs*. all sequence alignments between two species were imported into MCScanX [85] for collinearity analysis. Collinearity regions were visualized by JCVI v. 1.0.5 (https://zenodo.org/record/31631).

Transcriptome profiling of root and shoot during salt stress and recovery

The annual shoots were collected and cut into cuttings with a length of 20 cm from *T. chinensis* 'Lucheng No.1' on February 10, 2021. After washing with running tap water overnight, the cuttings were placed in three 58 × 33 × 15 cm containers, with 20 cuttings per container, and maintained in a hydroponic medium (half-strength Hoagland solution). As previously described, the cuttings were incubated in a growth chamber [86]. The medium was refreshed every seven days. After two months of culture, healthy 18-20 cm cutting clones with root lengths of 20-35 cm were selected and subjected to NaCl stress. Before NaCl stress was applied, the cutting clones were pre-treated in a medium containing 200 mM NaCl for two hours to avoid salt shock [87]. The cutting clones were grown for seven days on a hydroponic medium with 300 mM

NaCl before transferring to the hydroponic medium without NaCl for 35 d to recover. Seven-time points for sample collection were selected to cover early salt stress (including 300 mM NaCl stressed 0.5, 3, 5, and 8 h) and late salt stress and recovery (including 300 mM NaCl stressed 7 d and 35 d of recovery). Subsequently, 54 samples covering the seven-time points with three biological replicates per condition were harvested.

Total RNA from the roots and shoots was isolated according to previously described methods [37]. The RNA-seq library was prepared by the Illumina TruSeq RNA Sample Preparation Kit and sequenced on the Illumina NovaSeq6000 instrument using a PE-150 module. HiSAT2 (RRID:SCR_015530) v. 2.2.1 was used to map RNA-seq data to the *T. chinensis* genome, and featureCounts v. 2.0.3 [88] was used to quantify gene abundance. Principal coordinates analysis of gene expressions was performed using vegan v. 2.6-4 package in R v. 4.2. Integration of four differential analysis methods, including DEseq2 (RRID:SCR_015687) v. 1.34.0 [89], Limma (RRID:SCR_010943) v. 3.52.2 [90], ROTS v. 1.24.0 [91], and edgeR (RRID:SCR_012802) v. 3.38.4 [92], was performed to determine differentially expressed genes (DEGs), and all DEGs must satisfy $|\log2\ (\text{fold change})| \geq 1$ and false discovery rate ≤ 0.05. An intersection analysis of DEGs among different comparisons was performed by TBtools (RRID:SCR_023018) v. 1.098775 [93]. Dynamic gene expression analysis was performed by TCseq v. 1.22.0 package in R. Analysis of GO and KEGG categories enrichment was carried out by TBtools with whole-genome gene sets as background and a q value ≤ 0.05 as statistically significant.

Among plant species, *Arabidopsis* has the most abundant protein-protein interaction (PPI) data available, and most PPIs have been experimentally determined, so the protein sequences from the DEGs were BLAST-searched against the *A. thaliana* protein database for homolog identification. The best homology hits were retrieved and subjected to construct PPI network using the STRING (https://string-db.org/) database with a confidence score cut-off of 0.4. The

sub-network and hub genes were identified by CytoHubba in Cytoscape (RRID:SCR_003032) v. 3.9.1 [94, 95].

Quantitative real-time reverse-transcription PCR analysis

A randomly selected 27 DEGs were verified by quantitative real-time reverse-transcription PCR (qRT-PCR). PCR assays were performed on a Bio-Rad CFX Connect Real-Time instrument (Hercules, CA, USA) according to the procedure previously described [96]. Each sample was performed in three independent biological replicates. Relative abundance was quantified by normalizing it to the reference gene *TIF* [27].

Results

A chromosome-level *T. chinensis* genome assembly

K-mer frequency and flow cytometry analyses were performed to assess the genome size of *T. chinensis*. K-mer frequency analysis showed that the estimated genome size was about 1.45 Gb (Fig. S2a), close to the flow cytometry results (Fig. S2b). Thus, we evaluated the genome size of *T. chinensis* as 1.45 Gb.

PacBio and Hi-C data were integrated to construct a chromosome-level *T. chinensis* genome. First, by high-throughput sequencing, a total of 213.61 Gb (~147× genome coverage) PacBio long reads and 138.67 Gb (~95× genome coverage) Hi-C data were produced (Table S1). Next, PacBio data were used for genome assembly, resulting in a preliminary body of around 1.32 Gb genome sequence containing 342 contigs with an N50 size of 11.93 Mb (Table 1). Finally, Hi-C data were used to assign the contigs to correct chromosomal positions, showing that more than 99.5% of the preliminary assembly was anchored to 12 pseudochromosomes (Fig. 1a, S3; Table 1). Collectively, these results showed that the final genome assembly is 1.32 Gb

containing 63 super-scaffolds with an N50 value of 110.03 Mb.

Four approaches were used to evaluate genome assembly quality. First, assembly quality was assessed using BUSCO, revealing that 1571 of 1614 (97.4%) BUSCOs were captured entirely in the genome assembly (Fig. S4; Table S2). Next, Illumina and PacBio data were mapped to the genome assembly, revealing a high mapping rate of 99.84% (Illumina) and 94.31% (PacBio) and high coverage of 98.01% (Illumina) and 98.15% (PacBio), respectively. Then, transcripts assembled based on RNA sequencing data were aligned to the assembly, showing that 85,077 of 90,812 (93.68%) transcripts were assigned to the genome assembly. At last, Merqury estimated the assembly base accuracy and completeness, resulting in a high QV of 39.03 (Table 1). The above results suggested that the assembled *T. chinensis* genome was high quality in genome completeness, baseline accuracy, and contiguity.

Genome annotation

Genome annotation includes identifying repetitive elements and protein-coding genes. First, *de novo* and homology-based methods were used for identifying repetitive elements, resulting in around 0.98 Gb (74.24%) of repetitive elements in the genome sequence. Long-terminal repeat-retrotransposons (LTR-RTs), which accounted for 45.52 % of the whole genome sequence, were the most abundant elements (Table S3). Of these LTR-RTs, *Gypsy/DIRS1* and *Ty1/Copia* were the most common families, accounting for 22.13% and 12.06% of the total genome sequence.

By integrating *ab initio*, transcript-based, and homology-based methods, 26,426 high-confident protein-coding genes were identified, which exhibited an average of 5.30 exons and a mean length of 1233.60 bp (Table 1). The quality of gene predictions was assessed using BUSCO, revealing that 1515 of 1614 (93.9%) BUSCOs were captured entirely in predicted gene sets (Table S4). Subsequently, predicted genes were functionally annotated by scanning multiple

databases, resulting in 24,211 (91.62%) protein-coding genes exhibiting known functional annotations (Fig. S5).

Gene family evolution

To explore the evolutionary history of *T. chinensis*, a polygenetic tree based on 33 one-to-one orthologous genes shared across 32 angiosperm species, including 29 Caryophyllales species (5 with genome data and 27 with transcriptome data) and three outgroup species (Table S5), showed that *T. chinensis* and *T. hispida* diverged from the most recent common ancestor (MRCA) of *T. ramosissima c.*6.08 Ma ago, *Tamarix* split from the MRCA of *Reaumuria c.*50.24 Ma ago, and Tamaricaceae diverged from the MRCA of Frankeniaceae *c.* 78.5 Ma ago (Fig. S6).

Comparative genomics analysis was performed on 12 plant species with genome data, including 8 Caryophyllales species and three outgroups (*V. vinifera*, *A. thaliana*, and *O. sativa*) (Table S6). A total of 25,869 orthogroups were identified among *T. chinensis* and other species (Table S7). Of these orthogroups, 7097 orthogroups were shared among all species examined, of which 959 orthogroups contained single-copy genes (Fig. S7). A polygenetic tree was constructed to perform gene family expansion and contraction analysis, and the results showed that 2059 expansions and 10,336 contractions were identified in *T. chinensis* (Fig. 1b). P-values for each gene family were calculated, and 60 significant families ($p < 0.05$) were identified in *T. chinensis* (Table S8), including 56 expansions and four contractions, which had larger expansions than in *F. tataricum* (47), *Simmondsia chinensis* (32), and *H. undatus* (26). Functional annotation of the expansions showed that 23 families had known functional annotations (Fig. 1c; Table S9). Of which, 8 expansion gene families with 271 genes were annotated as *DAYSLEEPER* (*HAT*), which encodes a transposase-like protein playing essential

roles in regulating plant growth and development [97, 98]. The second largest expansion gene families (3 of 23; 141 genes) were annotated as *L10-interacting MYB domain-containing protein* (*LIMYB*), a transcriptional repressor involved in plant antiviral immunity [99]. In addition, two significant expansions (46 genes) were annotated as putative disease resistance, such as the *putative disease resistance RPP13-like protein 1* (*RPPL1*). These results suggested that significantly expanded gene families likely contributed to the high environmental adaptation of *T. chinensis*.

Whole-genome and tandem duplications associated with salinity adaptation in *T. chinensis*

K_S distribution of each pairwise paralog gene and inter-species syntenic analyses were performed to identify putative WGD events in the evolutionary history of *T. chinensis*. Analysis of K_S distributions revealed two distinct peaks in the *T. chinensis* genome (Fig. 2a). Inter-species syntenic analysis showed that *V. vinifera* and *T. chinensis* exhibited a 2:3 pattern for synthetic depth (Fig. 2b-c, S8), suggesting a more recent whole-genome duplication (WGD) event occurred in *T. chinensis*. A fitting curve on K_S distributions was performed. It showed that the WGD peak mainly ranged from 0.35 to 1.21 with a median of 0.61 (Fig. 2d), which was found to be shared by other species within Tamaricaceae (Fig. S9-10), indicating a Tamaricaceae-specific WGD event. Based on the time of divergence and mean peak K_S values of orthologous genes of syntenic blocks between *T. chinensis* and *V. vinifera*, Tamaricaceae synonymous nucleotide substitutions rate of was estimated to be 7.62×10^{-9} substitutions per site per year (Fig. S11), resulting in an estimated time of the WGD event *c.* 39.88 ± 12.95 Ma ago in the middle of Palaeogene (Fig. 1b).

To explore the differences in functions of gene duplications, a total of 19,935 duplications were identified and classified into five types: 4281 WGD genes (21.47%), 1130 tandem duplications

(TD, 5.67%), 1075 proximal duplications (PD, 5.39%), 4595 transposed duplications (TRD, 23.05%), and 8854 dispersed duplications (DSD, 44.41%) (Table S10). The K_a/K_s ratios of the five types of duplications were calculated and revealed that PD and TD exhibited higher K_a/K_s scores than any other type (Fig. 2e), suggesting rapid sequence divergence and strong positive selection in PD and TD duplications. A comparison of the expanded genes (EPGs) and each duplication type showed that WGD and DSD duplications accounted for more than 69% (6155 of 8857) of total EPGs (Fig. 2g), suggesting a critical contributor to gene family expansions (Fig. 2a, f). Analysis of gene ontology (GO) functional enrichment revealed different functions for the five duplications (Fig. 2h). For instance, WGD genes enriched GO terms implicated in the regulation of the biological and cellular process, cellular localization, and signaling, while TDs enriched GO categories involved in response to stress like oxidative stress and reactive oxygen species metabolic process. An essential process in plant response to salt stress is to sense and maintain ion homeostasis. There were 76 WGD duplications found to be involved in salt stress sensing and ion homeostasis (Fig. 3, S12; Table S11). For example, genes encoding GLR3.2, CNGC5/15, AKT1, SKOR, TPK1, HKT1, NHX2, and SOS3 were present in the WGD type. Ten genes encoding 14-3-3-like proteins as molecular switches in plant tolerance to salinity stress [100] were also present in the WGD type. These results suggested that WGD and tandem duplications were primarily related to salinity adaptation in *T. chinensis*.

Transcriptomic responses to salinity stress

A transcriptomic experiment was conducted to better understand the molecular mechanisms underlying high adaptation to salt stress of *T. chinensis*. The cutting clones were hydroponically grown for seven days on a hydroponic medium with 300 mM NaCl before being transferred to a hydroponic medium without NaCl for 35 d to recover (Fig. 4a). Seven-time points spanning

early salt stress, late salt stress, and recovery were selected. A total of 54 RNA sequencing libraries covering the seven-time points with three biological replicates per time point were generated and sequenced, producing more than 1.18 billion paired-end reads (Table S12). On average, each sample generated more than 21 million reads, of which more than 94% mapped to the *T. chinensis* genome assembly. Principal co-ordinates analysis of gene expressions revealed that root and shoot showed distinct gene expression profiles; peculiarly, the early salt stress, late stress, and recovery treatments on root also exhibited distinct gene expression profiles (Fig. 4b). Integration of four differential analysis methods, including DESeq2, edgeR, ROTS, and Limma, was performed to strengthen the identification of DEGs, generating 7118 and 6023 DEGs in root and shoot during early salt exposure, respectively (Fig. 4c; Table S13-15). Of those DEGs, there were 86 EPGs, 1653 WGD genes, 631 TD genes in the root; 77 EPGs, 1433 WGD genes, and 506 TD genes in the shoot. Meanwhile, 3675 and 6272 DEGs were identified in root and shoot during late salt stress and recovery, respectively (Fig. 4c; Table S13, Table S16-17). Among the DEGs, there were 51 EPGs, 852 WGD genes, and 431 TD genes in the root; 1489 EPGs, 1433 WGD genes, and 595 TD genes in the shoot. A randomly selected 27 DEGs were verified by qRT-PCR (Table S18), resulting in a significant and positive correlation between transcriptome results and qRT-PCR data, indicating the transcriptome data are reliable (Fig. 4d).

Identifying hub genes responding to early salt stress

The above-identified DEGs were further dissected to identify putative hub genes that respond to salinity stress during early salt stress. First, intersection analysis of DEGs (7118) at 0.5, 3, 5, and 8 h salt exposure in the root showed that more than 37% (2693 of 7118) of DEGs were shared among the four-time points (Fig. 5a). By analyzing the DEGs (6023) identified in the

shoot, it revealed a distinct two-phase pattern with more than 44% (2682 of 6023) of DEGs shared by the second time points from 5 to 8 h (Fig. 5b), suggesting a delayed response to salt stress in the shoot after salt exposure. Of the common root DEGs, 1219 and 1473 were respectively upregulated and downregulated at 0.5, 3, 5, and 8 h salt exposure (Fig. S13a, Table S19). Among those DEGs, 11 EPGs, 329 WGD genes, and 130 TD genes exhibited upregulated expression, and 20 EPGs, 328 WGD genes, and 149 TD genes exhibited downregulated expression at 0.5, 3, 5, and 8 h salt exposure. For example, *LIMYB* (*TC02G0567*) gene showed the largest difference with a 169.76 - 256.70-fold increase among the differentially expressed EPGs at 0.5, 3, 5, and 8 h salt exposure. Nine WRKY transcription factor coding genes (e.g., *WRKY33* and *WRKY75*) and several genes (e.g., *CBL4/SOS3*, *NHX2*, *AKT1*, and *CHX20*) involved in stress sensing and ion homeostasis showed upregulated at the four-time points. In the shoot, 961 and 1720 common DEGs were upregulated and downregulated at 5 and 8 h salt exposure, respectively (Fig. S13b, Table S20). Among the DEGs, 11 EPGs, 214 WGD genes, and 85 TD genes exhibited upregulated expression, and 12 EPGs, 450 WGD genes, and 111 TD genes exhibited downregulated expression at 5 and 8 h salt exposure. For instance, WGD genes associated with stress sensing like *CBL10* and *CSC1* (a Ca^{2+}-permeable channel coding gene) and ion transport like *CLC-C* and *potassium transporter 10* showed upregulated expressions at the two-time points.

GO category enrichment analyses showed that upregulated and downregulated DEGs exhibited different functions in root and shoot (Fig. 5c). Specifically, upregulated DEGs in both root and shoot enriched GO terms for cellular and biological process regulation, response to chemical, signaling, and cellular communication. Downregulated DEGs in both root and shoot enriched GO categories for microtubule-based process, cell wall organization or biogenesis, and cell cycle. The downregulated DEGs in the root specifically enriched GO terms for response to

oxidative stress and detoxification. Analysis of KEGG functional enrichment showed that upregulated DEGs in the root specifically enriched transcription factors, MAPK signaling pathway, signal transduction, and plant hormone signal transduction (Fig. 5d), whereas shoot specifically enriched term for ubiquitin system. In addition, DEGs downregulated in the root and shoot primarily enriched terms for metabolisms such as carbohydrate metabolism, nitrogen metabolism, and energy metabolism (specifically in the shoot).

Next, shared upregulated DEGs in both root and shoot were focused and used to identify hub genes. In the root, a PPI network with 1711 interactions consisting of 534 nodes and 1711 edges was constructed (Fig. S14, Table S21). Based on the degree of a node, 20 hub genes were identified in the root, with *WRKY33*, *WRKY40*, *MPK3*, *MPK4*, and *RHL41* being the most ranked genes (Fig. 5e). In the shoot, a PPI network was constructed with 689 interactions consisting of 400 nodes and 689 edges (Fig. S15, Table S22). A sub-network containing 20 hub genes was identified, of which *MPK3*, *XBAT31*, *DELTA-OAT*, *GRX480*, and *ALDA7B4* were the most ranked genes (Fig. 5f). These results suggested that the identified hub genes were likely to play an essential role in responding to salt stress during early exposure.

Identifying hub genes that respond to late salt stress and recovery

The effectiveness of the recovery mechanism following severe environmental conditions is crucial for plant survival. To understand the underlying recovery mechanisms of *T. chinensis* after salt stress, we performed a comparative transcriptome analysis on root and shoot tissues of cutting clones that were salt treated for 7 d (S/SC) and recovered with a hydroponic medium for up to 35 d (R/RC) (Fig. 4a).

In the root, a total of 2332 (1468 upregulated and 864 downregulated), 2781 (1537 upregulated and 1244 downregulated), and 800 (648 upregulated and 152 downregulated) DEGs were

identified in S *vs.* SC, R *vs.* S, and R *vs.* RC, respectively (Fig. 4c; Table S13), whereas only one upregulated DEG was identified in RC *vs.* SC. GO terms enrichment analyses on the DEGs of each comparison revealed that most of the salt-induced genes recovered at stage R (Fig. S16a). An intersection analysis of the upregulated and downregulated DEGs in each comparison identified 1705 DEGs with opposite trends between stress and recovery or maintained at the recovery stage, which included 20 EPGs (e.g., *LIMYB* and *RPPL1*), 386 WGD genes (e.g., *CSC1*, *HKT1*, *TPK1*, and *CHX20*), and 198 TD genes (e.g., 12 class III peroxidase coding genes, including *PER26*, *PER52*, *PER56*, *PER57*, and *PER60*) (Fig. S17a, c, Table S23). Gene dynamics analyses on the DEGs identified eight clusters, which were further classified into four groups according to gene expression trends (Fig. 6a). Clusters 1, 4, and 5 were grouped into G1, consisting of 557 DEGs exhibiting downregulation at stage S and then recovered at stage R. Clusters 2, 6, and 8 were grouped into G2, consisting of 856 DEGs exhibiting upregulation at stage S and then recovered at stage R. Cluster 7 (G3) and cluster 3 (G4) comprised 255 and 37 DEGs exhibiting maintained upregulation or downregulation at both stages S and R, respectively. GO category enrichment analysis revealed a distinct difference in gene functions (Fig. 6c): G1 enriched genes involved in defense response and biological and cellular process regulation; G2 enriched genes related to microtubule-based process, cell cycle, cell wall organization or biogenesis, and secondary metabolic process; whereas G3 gathered genes participated in carbohydrate metabolic process, response to oxidative stress, response to stress, and cellular catabolic process. PPI network analyses of the DEGs in each group identified three networks (Fig. S17a; Table S24-26): G1 had a network with 448 interactions composed of 71 nodes and 170 edges; G2 had a network exhibiting 3691 interactions consisting of 135 nodes and 2555 edges; G3 had a network with 169 interactions composed of 30 nodes and 98 edges. Based on the degree of a node, a sub-network consisting of 10 hub genes was identified

in each group, with *WRKY40* and *ZAT10* being the most ranked central genes in G1; *KIN10A* and *CDKB2-2* being the most ranked in G2; while *PER7*, *PER27*, *PER57*, and *PER73* were the most ranked hub genes in G3 (Fig. 6e).

In the shoot, a total of 2831 (983 upregulated and 1843 downregulated), 3486 (1897 upregulated and 1589 downregulated), 1376 (818 upregulated and 558 downregulated), and 3413 (1439 upregulated and 1974 downregulated) DEGs were identified in S *vs.* SC, R *vs.* S, R *vs.* RC, and RC *vs.* SC, respectively (Fig. 4c; Table S13). GO category enrichment analyses of the DEGs of each comparison revealed various terms related to metabolic process, biological and cellular process regulation, and response to stimulus (Fig. S16b). Similar to the root, a total of 612 DEGs were identified, showing opposite trends between stages S and R or maintained at stage R, which included 16 EPGs (e.g., *HAT* and *RPPL1*), 154 WGD genes (e.g., *GLR3.2* and *Ca^{2+}/H^{+} antiporter CAX1/3*), and 73 TD genes (e.g., *PER72*, *GSTT1*, and *GSTU8*) (Fig. S17b, d, Table S27). Gene expression dynamics analyses showed that DEGs were grouped into eight clusters, which were further classified into four major groups (Fig. 6b). Clusters 1, 2, and 7 were grouped into G1 consisting of 288 downregulated DEGs at stage S and then recovered at stage R, while clusters 3, 4, and 6 were grouped into G2 consisting of 212 upregulated DEGs at S stage and then recovered at R stage. Cluster 8 (G3) and cluster 5 (G4) consisted of 78 and 34 DEGs, representing maintained upregulated or downregulated at both stages S and R, respectively. GO category analyses on the DEGs in each group showed that G2 enriched various terms mainly related to the regulation of biological, cellular catabolic, and metabolic processes, whereas G3 enriched genes involved in the chromosome, organelle, and cellular component organization, and macromolecule metabolic process (Fig. 6d). PPI network analyses for each group DEGs identified three networks (Fig. S18b; Table S28-30). Among the networks, G1 had 117 interactions comprising 46 nodes and 48 edges; G2 had 160 interactions with 28 nodes and

100 edges; and G3 exhibited 168 interactions with 28 nodes and 165 edges. Subsequently, a sub-network consisting of 10 central genes was identified for each group according to the degree of a node (Fig. 6f). Among the sub-networks, *CYP86A1* and *AHK4* were the most ranked hub genes in G1, *IRX9*, *CESA4*, and *CESA8* were the most ranked central genes in G2; for G3, *MCM3*, *MCM4*, *MCM5*, *MCM7*, and *RNR1* were the most ranked hub genes. These results suggested that the central genes identified in root and shoot probably played an essential role in responding to salt stress during late salt stress and recovery.

Discussion

This work described a chromosome-level genome for *T. chinensis,* a pioneer tree species of the coastal wetland ecosystem in Northern China. We found that the families of *HAT* and *LIMYB* genes were significantly expanded in the *T. chinensis* genome, of which *HAT* is essential for plant growth and development [97, 98], and *LIMYB* as a transcriptional repressor functioned in plant antiviral immunity [99], likely suggestive of the critical roles in high environmental adaptation. We dated a WGD event in Tamaricaceae lineage *c.* 39.88 ± 12.95 Ma in the middle of Palaeogene. It is suggested that the WGD event was shared between *Tamarix* and *Reaumuria*, as previously suggested by dense phylogenomic sampling across Caryophyllales [101]. WGD and TD duplications are critical drivers in plant adaptive evolution to enhance high tolerance to environmental stress [36, 102-106]. We found that WGD and TD in *T. chinensis* contributed gene duplications involved in salt stress sensing, ion homeostasis, response to stress like oxidative stress, and reactive oxygen species metabolic process, suggestive of significant contributors in high salinity adaptation of *T. chinensis.*

During early salt stress, we found that more than 37% of DEGs were shared among the four-time points in the root. In contrast, the shoot exhibited a distinct two-phase pattern, with more

than 44% of DEGs shared only by time points from 5 to 8 h, suggesting a delayed response to salt stress in the shoot rather than root after salt exposure. *WRKY* transcription factors are crucial regulators of a plant responding to salinity stress [107, 108]. For example, *WRKY33* is a vital transcriptional regulator involved in multiple regulatory networks to promote plant salt tolerance [109-112]. In *Pyrus betulaefolia*, *WRKY40* positively regulates a V-type-H^+-ATPase gene to promote salt tolerance and organic acid accumulation [113]. In *Fortunella crassifolia*, *WRKY40* positively regulates *Salt Overly Sensitive 2* (*SOS2*) and *Δ-1-pyrroline-5-carboxylate synthetase 1* (*P5CS1*) homologs to enhance salt tolerance [114]. We found that *WRKY33* and *WRKY40* transcription factors were the most ranked hub genes in the root during early salt stress, indicating their essential roles in enhancing *T. chinensis* tolerance. MAPK cascade is an essential pathway that regulates plants' responses to multiple environmental stresses [115]. For example, *MPK3*, a positive regulator, regulates the lipid transfer protein AZI1 to improve stress resistance in *Arabidopsis* [116, 117]. *MPK3/6* is a negative regulator degrading several *Arabidopsis* response regulators to enhance salt tolerance [118]. The OsMKK1-OsMPK4 signaling pathway regulates salt resistance in rice [119]. This study identified *MPK3* and *MPK4* as the most ranked hub genes during early salt stress, suggesting the critical roles in *T. chinensis* against salt stress. We also found several pivotal hub genes in plants responding to various environmental stresses. For instance, *XBAT31*, one of the most ranked central genes in the shoot, is an E3 ligase that responds to warm temperatures by mediating ELF3 (a thermosensor) degradation in *Arabidopsis [120]*. *DELTA-OAT*, another central gene in the shoot, encodes an ornithine-delta-aminotransferase essential for resistance to non-host disease in *Arabidopsis* [121]. These results suggest that the identified hub genes played an essential role in responding to salt stress during early exposure and may be critical gene resources used for salt-tolerant plant genetic improvement.

This study identified several hub genes related to plant recovery after salt stress. We found that *WRKY40*, *ZAT10*, *KIN10A*, and *CDKB2-2* were the most ranked hub genes associated with stress recovery in the root, of which the first two genes were downregulated at the stress stage, while the last two were upregulated at the stress stage. *WRKY40*, as a key regulator of salt-responsive genes, is shared between early salt stress and late stress recovery but has opposite expression patterns, suggesting a dual regulatory role in plant response to salt stress [122]. *ZAT10*, a zinc-finger transcription factor, exhibited dual roles in promoting plant salt tolerance [123] and cadmium uptake and detoxification [124]. In the shoot, *CYP86A1* and *AHK4* hub genes were downregulated, while *IRX9*, *CESA4*, and *CESA8* were upregulated at the stress stage. Among these genes, *AHK4* encoding a histidine kinase is a cytokinin receptor sensing environmental signals that function as negative regulators in response to osmotic stress [125, 126]. The hub genes *IRX9*, *CESA4*, and *CESA8* were associated with plant secondary cell wall formation [127-129], suggesting functional adaptation of secondary wall genes under abiotic stress [130]. We found that *PER7*, *PER27*, *PER57*, and *PER73* were the most ranked hub genes in root, which encode class III peroxidases that functioned as an antioxidant for biotic or abiotic stress resistance in plants [131], suggesting their essential roles in adapting to salt stress. While in the shoot, *MCM3*, *MCM4*, *MCM5*, and *MCM7* were the most ranked central genes, which are the components of the minichromosome maintenance complex, played crucial roles in DNA replication initiation and cell division [132], suggesting that cell division was likely associated with stress response [133].

In summary, this study first described the nearly complete reference genome of halophyte *T. chinensis*. Gene families related to plant growth and development have significantly expanded in *T. chinensis*. Whole-genome and tandem duplications contributed to the expansion of genes involved in salinity adaptation in *T. chinensis*. Several hub genes were identified as responding

to salt stress in *T. chinensis*, but more validation experiments were needed. Therefore, this study will be a valuable genetic resource for investigating the evolutionary adaptation of tamarisk and the genetic improvement of plant salt tolerance.

Data Availability

The genome sequencing data, including PacBio long reads, Illumina short reads, and Hi-C data, are available via NCBI with BioProject accession PRJNA855314. The RNA sequencing data are deposited in the NCBI under accession PRJNA855335. This Whole Genome Shotgun project has been deposited at DDBJ/ENA/GenBank under the accession JANKMZ000000000. The version described in this paper is version JANKMZ010000000.

Additional supporting data, also including BUSCO and Merqury results, are available via the *GigaScience* database GigaDB [134].

Ethics Approval and Consent to Participate

No ethical approval/permission is required to obtain the materials and perform the research in this study.

Competing Interests

The authors declare that they have no competing interests.

Fundings

This work was supported by the Improved Variety Program of Shandong Province of China (2019LZGC009).

Author Contributions

K.Q.Y., and D.J.W. conceived the study; J.N.L., H.C.F., Q.L., Y.H.D., L.P.Y., X.Y.L., S.S.G., S.Y.X., and L.C.W. analyzed the data; J.N.L., C.X.W., X.M.M, and R.Z., collected materials; J.N.L. wrote the original draft manuscript; J.N.L., K.Q.Y., and D.J.W. reviewed and edited the

manuscript. All authors read and approved the final manuscript.

References

1. Chele KH, Tinte MM, Piater LA, Dubery IA and Tugizimana F. Soil salinity, a serious environmental issue and plant responses: A metabolomics perspective. Metabolites. 2021; 11(11):724. doi:10.3390/metabo11110724.
2. Zhu JK. Abiotic stress signaling and responses in plants. Cell. 2016;167(2):313-324. doi:10.1016/j.cell.2016.08.029.
3. Mahajan S and Tuteja N. Cold, salinity and drought stresses: an overview. Arch Biochem Biophys. 2005;444(2):139-158. doi:10.1016/j.abb.2005.10.018.
4. Van Zelm E, Zhang Y and Testerink C. Salt tolerance mechanisms of plants. Annu Rev Plant Biol. 2020;71:403-433. doi:10.1146/annurev-arplant-050718-100005.
5. Deinlein U, Stephan AB, Horie T, Luo W, Xu G and Schroeder JI. Plant salt-tolerance mechanisms. Trends Plant Sci. 2014;19(6):371-379. doi:10.1016/j.tplants.2014.02.001.
6. Zhao S, Zhang Q, Liu M, Zhou H, Ma C and Wang P. Regulation of plant responses to salt stress. Int J Mol Sci. 2021;22(9):4609. doi:10.3390/ijms22094609.
7. Verret F, Wheeler G, Taylor AR, Farnham G and Brownlee C. Calcium channels in photosynthetic eukaryotes: implications for evolution of calcium-based signalling. New Phytol. 2010;187(1):23-43. doi:10.1111/j.1469-8137.2010.03271.x.
8. Jin Y, Jing W, Zhang Q and Zhang W. Cyclic nucleotide gated channel 10 negatively regulates salt tolerance by mediating Na^+ transport in *Arabidopsis*. J Plant Res. 2015;128(1):211-220. doi:10.1007/s10265-014-0679-2.
9. Zhang H, Zhu J, Gong Z and Zhu JK. Abiotic stress responses in plants. Nat Rev Genet. 2022;23(2):104-119. doi:10.1038/s41576-021-00413-0.

10. Jiang J, Ma S, Ye N, Jiang M, Cao J and Zhang J. WRKY transcription factors in plant responses to stresses. J Integr Plant Biol. 2017;59(2):86-101. doi:10.1111/jipb.12513.

11. Ishihama N and Yoshioka H. Post-translational regulation of WRKY transcription factors in plant immunity. Curr Opin Plant Biol. 2012;15(4):431-437. doi:10.1016/j.pbi.2012.02.003.

12. Yang Y and Guo Y. Elucidating the molecular mechanisms mediating plant salt-stress responses. New Phytol. 2018;217(2):523-539. doi:https://doi.org/10.1111/nph.14920.

13. Gaymard F, Pilot G, Lacombe B, Bouchez D, Bruneau D, Boucherez J, et al. Identification and disruption of a plant shaker-like outward channel involved in K^+ release into the xylem sap. Cell. 1998;94(5):647-655. doi:10.1016/s0092-8674(00)81606-2.

14. Bihler H, Eing C, Hebeisen S, Roller A, Czempinski K and Bertl A. TPK1 is a vacuolar ion channel different from the slow-vacuolar cation channel. Plant Physiol. 2005;139(1):417-424. doi:10.1104/pp.105.065599.

15. Platten JD, Cotsaftis O, Berthomieu P, Bohnert H, Davenport RJ, Fairbairn DJ, et al. Nomenclature for HKT transporters, key determinants of plant salinity tolerance. Trends Plant Sci. 2006;11(8):372-374. doi:10.1016/j.tplants.2006.06.001.

16. Apse MP, Sottosanto JB and Blumwald E. Vacuolar cation/H^+ exchange, ion homeostasis, and leaf development are altered in a T-DNA insertional mutant of AtNHX1, the *Arabidopsis* vacuolar Na^+/H^+ antiporter. Plant J. 2003;36(2):229-239. doi:10.1046/j.1365-313x.2003.01871.x.

17. Bassil E, Zhang S, Gong H, Tajima H and Blumwald E. Cation specificity of vacuolar NHX-Type Cation/H^+ antiporters. Plant Physiol. 2019;179(2):616-629. doi:10.1104/pp.18.01103.

18. Subba A, Tomar S, Pareek A and Singla-Pareek SL. The chloride channels: Silently

serving the plants. Physiol Plant. 2021;171(4):688-702. doi:10.1111/ppl.13240.
19. Qi F and Zhang F. Cell cycle regulation in the plant response to stress. Front Plant Sci. 2019;10:1765. doi:10.3389/fpls.2019.01765.
20. Rahman MM, Mostofa MG, Keya SS, Siddiqui MN, Ansary MMU, Das AK, et al. Adaptive mechanisms of halophytes and their potential in improving salinity tolerance in plants. Int J Mol Sci. 2021;22(19):10733. doi:10.3390/ijms221910733.
21. Yuan F, Guo J, Shabala S and Wang B. Reproductive physiology of halophytes: Current standing. Front Plant Sci. 2018;9:1954. doi:10.3389/fpls.2018.01954.
22. Zhang J-W, D'Rozario A, Duan S-M, Wang X-Y, Liang X-Q and Pan B-R. Epidermal characters of *Tamarix* L. (Tamaricaceae) from Northwest China and their taxonomic and palaeogeographic implications. J Palaeogeog. 2018;7(2):179-196. doi:10.1016/j.jop.2018.01.003.
23. Villar JL, Alonso MÁ, Juan A, Gaskin JF and Crespo MB. Out of the Middle East: New phylogenetic insights in the genus *Tamarix* (Tamaricaceae). J Syst Evol. 2019;57(5):488-507. doi:10.1111/jse.12478.
24. Duan Q, Zhu Z, Wang B and Chen M. Recent progress on the salt tolerance mechanisms and application of tamarisk. Int J Mol Sci. 2022;23(6):3325. doi:10.3390/ijms23063325.
25. Feng X, Liu X, Zhang X and Li JS. Growth dynamic of *Tamarix chinensis* plantations in high salinity coastal land and its ecological effect. In: Gul B, Böer B, Khan MA, Clüsener-Godt M and Hameed A, editors. Sabkha Ecosystems: Volume VI: Asia/Pacific. Cham: Springer International Publishing; 2019. p. 113-24.
26. Tang J, Ye S, Chen X, Yang H, Sun X, Wang F, et al. Coastal blue carbon: Concept, study method, and the application to ecological restoration. Sci China Earth Sci. 2018;61(6):637-646. doi:10.1007/s11430-017-9181-x.

27. Yang H, Xia J, Cui Q, Liu J, Wei S, Feng L, et al. Effects of different *Tamarix chinensis*-grass patterns on the soil quality of coastal saline soil in the Yellow River Delta, China. Sci Total Environ. 2021;772:145501. doi:10.1016/j.scitotenv.2021.145501.

28. Wang J, Ye Y, Xu M, Feng L and Xu LA. Roles of the *SPL* gene family and miR156 in the salt stress responses of tamarisk (*Tamarix chinensis*). BMC Plant Biol. 2019;19(1):370. doi:10.1186/s12870-019-1977-6.

29. Ding F, Yang J-C, Yuan F and Wang B-S. Progress in mechanism of salt excretion in recretohalopytes. Front Biol. 2010;5(2):164-170. doi:10.1007/s11515-010-0032-7.

30. Ma T, Wang J, Zhou G, Yue Z, Hu Q, Chen Y, et al. Genomic insights into salt adaptation in a desert poplar. Nat Commun. 2013;4:2797. doi:10.1038/ncomms3797.

31. Feng X, Li G, Xu S, Wu W, Chen Q, Shao S, et al. Genomic insights into molecular adaptation to intertidal environments in the mangrove *Aegiceras corniculatum*. New Phytol. 2021;231(6):2346-2358. doi:10.1111/nph.17551.

32. Ma D, Ding Q, Guo Z, Xu C, Liang P, Zhao Z, et al. The genome of a mangrove plant, *Avicennia marina*, provides insights into adaptation to coastal intertidal habitats. Planta. 2022;256(1):6. doi:10.1007/s00425-022-03916-0.

33. Natarajan P, Murugesan AK, Govindan G, Gopalakrishnan A, Kumar R, Duraisamy P, et al. A reference-grade genome identifies salt-tolerance genes from the salt-secreting mangrove species *Avicennia marina*. Commun Biol. 2021;4(1):851. doi:10.1038/s42003-021-02384-8.

34. Yang Y, Bocs S, Fan H, Armero A, Baudouin L, Xu P, et al. Coconut genome assembly enables evolutionary analysis of palms and highlights signaling pathways involved in salt tolerance. Commun Biol. 2021;4(1):105. doi:10.1038/s42003-020-01593-x.

35. Yuan F, Wang X, Zhao B, Xu X, Shi M, Leng B, et al. The genome of the recretohalophyte

Limonium bicolor provides insights into salt gland development and salinity adaptation during terrestrial evolution. Mol Plant. 2022;15(6):1024-1044. doi:10.1016/j.molp.2022.04.011.

36. Ren G, Jiang Y, Li A, Yin M, Li M, Mu W, et al. The genome sequence provides insights into salt tolerance of *Achnatherum splendens* (Gramineae), a constructive species of alkaline grassland. Plant Biotechnol J. 2022;20(1):116-128. doi:10.1111/pbi.13699.
37. Liang Q, Li H, Li S, Yuan F, Sun J, Duan Q, et al. The genome assembly and annotation of yellowhorn (*Xanthoceras sorbifolium* Bunge). Gigascience. 2019;8(6):giz071. doi:10.1093/gigascience/giz071.
38. Sikorskaite S, Rajamäki ML, Baniulis D, Stanys V and Valkonen JP. Protocol: Optimised methodology for isolation of nuclei from leaves of species in the Solanaceae and Rosaceae families. Plant Methods. 2013;9:31. doi:10.1186/1746-4811-9-31.
39. Schnable PS, Ware D, Fulton RS, Stein JC, Wei F, Pasternak S, et al. The B73 maize genome: complexity, diversity, and dynamics. Science. 2009;326(5956):1112-1115. doi:10.1126/science.1178534.
40. Marçais G and Kingsford C. A fast, lock-free approach for efficient parallel counting of occurrences of k-mers. Bioinformatics. 2011;27(6):764-770. doi:10.1093/bioinformatics/btr011.
41. Bolger AM, Lohse M and Usadel B. Trimmomatic: a flexible trimmer for Illumina sequence data. Bioinformatics. 2014;30(15):2114-2120. doi:10.1093/bioinformatics/btu170.
42. Liu C. In situ Hi-C library preparation for plants to study their three-dimensional chromatin interactions on a genome-wide scale. Methods Mol Biol. 2017;1629:155-166. doi:10.1007/978-1-4939-7125-1_11.

43. Koren S, Walenz BP, Berlin K, Miller JR, Bergman NH and Phillippy AM. Canu: scalable and accurate long-read assembly via adaptive k-mer weighting and repeat separation. Genome Res. 2017;27(5):722-736. doi:10.1101/gr.215087.116.

44. Guan D, McCarthy SA, Wood J, Howe K, Wang Y and Durbin R. Identifying and removing haplotypic duplication in primary genome assemblies. Bioinformatics. 2020;36(9):2896-2898. doi:10.1093/bioinformatics/btaa025.

45. Walker BJ, Abeel T, Shea T, Priest M, Abouelliel A, Sakthikumar S, et al. Pilon: an integrated tool for comprehensive microbial variant detection and genome assembly improvement. PLoS One. 2014;9(11):e112963. doi:10.1371/journal.pone.0112963.

46. Seppey M, Manni M and Zdobnov EM. BUSCO: Assessing genome assembly and annotation completeness. Methods Mol Biol. 2019;1962:227-245. doi:10.1007/978-1-4939-9173-0_14.

47. Durand NC, Shamim MS, Machol I, Rao SS, Huntley MH, Lander ES, et al. Juicer provides a one-click system for analyzing loop-resolution Hi-C experiments. Cell Syst. 2016;3(1):95-98. doi:10.1016/j.cels.2016.07.002.

48. Dudchenko O, Batra SS, Omer AD, Nyquist SK, Hoeger M, Durand NC, et al. De novo assembly of the *Aedes aegypti* genome using Hi-C yields chromosome-length scaffolds. Science. 2017;356(6333):92-95. doi:10.1126/science.aal3327.

49. Durand NC, Robinson JT, Shamim MS, Machol I, Mesirov JP, Lander ES, et al. Juicebox provides a visualization system for Hi-C contact maps with unlimited zoom. Cell Syst. 2016;3(1):99-101. doi:10.1016/j.cels.2015.07.012.

50. Akdemir KC and Chin L. HiCPlotter integrates genomic data with interaction matrices. Genome Biol. 2015;16(1):198. doi:10.1186/s13059-015-0767-1.

51. Langmead B and Salzberg SL. Fast gapped-read alignment with Bowtie 2. Nat Methods.

2012;9(4):357-359. doi:10.1038/nmeth.1923.

52. Grabherr MG, Haas BJ, Yassour M, Levin JZ, Thompson DA, Amit I, et al. Full-length transcriptome assembly from RNA-Seq data without a reference genome. Nat Biotechnol. 2011;29(7):644-652. doi:10.1038/nbt.1883.
53. Kent WJ. BLAT--the BLAST-like alignment tool. Genome Res. 2002;12(4):656-664. doi:10.1101/gr.229202.
54. Rhie A, Walenz BP, Koren S and Phillippy AM. Merqury: Reference-free quality, completeness, and phasing assessment for genome assemblies. Genome Biol. 2020;21(1):245. doi:10.1186/s13059-020-02134-9.
55. Ou S, Su W, Liao Y, Chougule K, Agda JRA, Hellinga AJ, et al. Benchmarking transposable element annotation methods for creation of a streamlined, comprehensive pipeline. Genome Biol. 2019;20(1):275. doi:10.1186/s13059-019-1905-y.
56. Tarailo-Graovac M and Chen N. Using RepeatMasker to identify repetitive elements in genomic sequences. Curr Protoc Bioinformatics. 2009;Chapter 4:4.10.1-4.10.14. doi:10.1002/0471250953.bi0410s25.
57. Cantarel BL, Korf I, Robb SM, Parra G, Ross E, Moore B, et al. MAKER: an easy-to-use annotation pipeline designed for emerging model organism genomes. Genome Res. 2008;18(1):188-196. doi:10.1101/gr.6743907.
58. Haas BJ, Papanicolaou A, Yassour M, Grabherr M, Blood PD, Bowden J, et al. De novo transcript sequence reconstruction from RNA-seq using the Trinity platform for reference generation and analysis. Nat Protoc. 2013;8(8):1494-1512. doi:10.1038/nprot.2013.084.
59. Korf I. Gene finding in novel genomes. BMC Bioinformatics. 2004;5:59. doi:10.1186/1471-2105-5-59.
60. Besemer J, Lomsadze A and Borodovsky M. GeneMarkS: a self-training method for

prediction of gene starts in microbial genomes. Implications for finding sequence motifs in regulatory regions. Nucleic Acids Res. 2001;29(12):2607-2618. doi:10.1093/nar/29.12.2607.

61. Stanke M, Schöffmann O, Morgenstern B and Waack S. Gene prediction in eukaryotes with a generalized hidden Markov model that uses hints from external sources. BMC Bioinformatics. 2006;7:62. doi:10.1186/1471-2105-7-62.

62. Slater GS and Birney E. Automated generation of heuristics for biological sequence comparison. BMC Bioinformatics. 2005;6:31. doi:10.1186/1471-2105-6-31.

63. Dohm JC, Minoche AE, Holtgräwe D, Capella-Gutiérrez S, Zakrzewski F, Tafer H, et al. The genome of the recently domesticated crop plant sugar beet (*Beta vulgaris*). Nature. 2014;505(7484):546-549. doi:10.1038/nature12817.

64. Xu C, Jiao C, Sun H, Cai X, Wang X, Ge C, et al. Draft genome of spinach and transcriptome diversity of 120 Spinacia accessions. Nat Commun. 2017;8:15275. doi:10.1038/ncomms15275.

65. Jaillon O, Aury JM, Noel B, Policriti A, Clepet C, Casagrande A, et al. The grapevine genome sequence suggests ancestral hexaploidization in major angiosperm phyla. Nature. 2007;449(7161):463-467. doi:10.1038/nature06148.

66. Lamesch P, Berardini TZ, Li D, Swarbreck D, Wilks C, Sasidharan R, et al. The Arabidopsis Information Resource (TAIR): Improved gene annotation and new tools. Nucleic Acids Res. 2012;40(Database issue):D1202-D1210. doi:10.1093/nar/gkr1090.

67. Tomato Genome Consortium. The tomato genome sequence provides insights into fleshy fruit evolution. Nature. 2012;485(7400):635-641. doi:10.1038/nature11119.

68. Tuskan GA, DiFazio S, Jansson S, Bohlmann J, Grigoriev I, Hellsten U, et al. The genome of black cottonwood, *Populus trichocarpa* (Torr. & Gray). Science.

2006;313(5793):1596. doi:10.1126/science.1128691.

69. Ouyang S, Zhu W, Hamilton J, Lin H, Campbell M, Childs K, et al. The TIGR rice genome annotation resource: Improvements and new features. Nucleic Acids Res. 2007;35(Database issue):D883-D887. doi:10.1093/nar/gkl976.

70. Jones P, Binns D, Chang HY, Fraser M, Li W, McAnulla C, et al. InterProScan 5: Genome-scale protein function classification. Bioinformatics. 2014;30(9):1236-1240. doi:10.1093/bioinformatics/btu031.

71. Hunter S, Apweiler R, Attwood TK, Bairoch A, Bateman A, Binns D, et al. InterPro: The integrative protein signature database. Nucleic Acids Res. 2009;37(Database issue):D211-D215. doi:10.1093/nar/gkn785.

72. Aramaki T, Blanc-Mathieu R, Endo H, Ohkubo K, Kanehisa M, Goto S, et al. KofamKOALA: KEGG Ortholog assignment based on profile HMM and adaptive score threshold. Bioinformatics. 2020;36(7):2251-2252. doi:10.1093/bioinformatics/btz859.

73. Yang Y, Moore MJ, Brockington SF, Soltis DE, Wong GK, Carpenter EJ, et al. Dissecting molecular evolution in the highly diverse plant clade Caryophyllales using transcriptome sequencing. Mol Biol Evol. 2015;32(8):2001-2014. doi:10.1093/molbev/msv081.

74. Yang Y and Smith SA. Orthology inference in nonmodel organisms using transcriptomes and low-coverage genomes: Improving accuracy and matrix occupancy for phylogenomics. Mol Biol Evol. 2014;31(11):3081-3092. doi:10.1093/molbev/msu245.

75. Emms DM and Kelly S. OrthoFinder: Phylogenetic orthology inference for comparative genomics. Genome Biol. 2019;20(1):238. doi:10.1186/s13059-019-1832-y.

76. Katoh K and Standley DM. MAFFT multiple sequence alignment software version 7: Improvements in performance and usability. Mol Biol Evol. 2013;30(4):772-780. doi:10.1093/molbev/mst010.

77. Stamatakis A. RAxML version 8: A tool for phylogenetic analysis and post-analysis of large phylogenies. Bioinformatics. 2014;30(9):1312-1313. doi:10.1093/bioinformatics/btu033.

78. Yang Z. PAML 4: Phylogenetic analysis by maximum likelihood. Mol Biol Evol. 2007;24(8):1586-1591. doi:10.1093/molbev/msm088.

79. Kumar S, Stecher G, Suleski M and Hedges SB. TimeTree: A resource for timelines, timetrees, and divergence times. Mol Biol Evol. 2017;34(7):1812-1819. doi:10.1093/molbev/msx116.

80. Mendes FK, Vanderpool D, Fulton B and Hahn MW. CAFE 5 models variation in evolutionary rates among gene families. Bioinformatics. 2020;36(22-23):5516-5518. doi:10.1093/bioinformatics/btaa1022.

81. Zwaenepoel A and Van de Peer Y. wgd-simple command line tools for the analysis of ancient whole-genome duplications. Bioinformatics. 2019;35(12):2153-2155. doi:10.1093/bioinformatics/bty915.

82. Qiao X, Li Q, Yin H, Qi K, Li L, Wang R, et al. Gene duplication and evolution in recurring polyploidization-diploidization cycles in plants. Genome Biol. 2019;20(1):38. doi:10.1186/s13059-019-1650-2.

83. Zhang Z, Li J, Zhao XQ, Wang J, Wong GK and Yu J. KaKs_Calculator: Calculating Ka and Ks through model selection and model averaging. Genomics Proteomics Bioinformatics. 2006;4(4):259-263. doi:10.1016/s1672-0229(07)60007-2.

84. Suyama M, Torrents D and Bork P. PAL2NAL: Robust conversion of protein sequence alignments into the corresponding codon alignments. Nucleic Acids Res. 2006;34(Web Server issue):W609-W612. doi:10.1093/nar/gkl315.

85. Wang Y, Tang H, Debarry JD, Tan X, Li J, Wang X, et al. MCScanX: A toolkit for detection

and evolutionary analysis of gene synteny and collinearity. Nucleic Acids Res. 2012;40(7):e49. doi:10.1093/nar/gkr1293.

86. Liu JN, Ma X, Yan L, Liang Q, Fang H, Wang C, et al. MicroRNA and degradome profiling uncover defense response of *Fraxinus velutina* Torr. to salt stress. Front Plant Sci. 2022;13:847853. doi:10.3389/fpls.2022.847853.
87. Shavrukov Y. Salt stress or salt shock: which genes are we studying? J Exp Bot. 2013;64(1):119-127. doi:10.1093/jxb/ers316.
88. Liao Y, Smyth GK and Shi W. featureCounts: An efficient general purpose program for assigning sequence reads to genomic features. Bioinformatics. 2014;30(7):923-930. doi:10.1093/bioinformatics/btt656.
89. Love MI, Huber W and Anders S. Moderated estimation of fold change and dispersion for RNA-seq data with DESeq2. Genome Biol. 2014;15(12):550. doi:10.1186/s13059-014-0550-8.
90. Ritchie ME, Phipson B, Wu D, Hu Y, Law CW, Shi W, et al. limma powers differential expression analyses for RNA-sequencing and microarray studies. Nucleic Acids Res. 2015;43(7):e47. doi:10.1093/nar/gkv007.
91. Suomi T, Seyednasrollah F, Jaakkola MK, Faux T and Elo LL. ROTS: An R package for reproducibility-optimized statistical testing. PLoS Comput Biol. 2017;13(5):e1005562. doi:10.1371/journal.pcbi.1005562.
92. Robinson MD, McCarthy DJ and Smyth GK. edgeR: A Bioconductor package for differential expression analysis of digital gene expression data. Bioinformatics. 2009;26(1):139-140. doi:10.1093/bioinformatics/btp616.
93. Chen C, Chen H, Zhang Y, Thomas HR, Frank MH, He Y, et al. TBtools: An integrative toolkit developed for interactive analyses of big biological data. Mol Plant.

2020;13(8):1194-1202. doi:10.1016/j.molp.2020.06.009.

94. Chin CH, Chen SH, Wu HH, Ho CW, Ko MT and Lin CY. cytoHubba: Identifying hub objects and sub-networks from complex interactome. BMC Syst Biol. 2014;8 Suppl 4(Suppl 4):S11. doi:10.1186/1752-0509-8-s4-s11.

95. Shannon P, Markiel A, Ozier O, Baliga NS, Wang JT, Ramage D, et al. Cytoscape: A software environment for integrated models of biomolecular interaction networks. Genome Res. 2003;13(11):2498-2504. doi:10.1101/gr.1239303.

96. Fang H, Liu X, Dong Y, Feng S, Zhou R, Wang C, et al. Transcriptome and proteome analysis of walnut (*Juglans regia* L.) fruit in response to infection by *Colletotrichum gloeosporioides*. BMC Plant Biol. 2021;21(1):249. doi:10.1186/s12870-021-03042-1.

97. Bundock P and Hooykaas P. An *Arabidopsis* hAT-like transposase is essential for plant development. Nature. 2005;436(7048):282-284. doi:10.1038/nature03667.

98. Knip M, de Pater S and Hooykaas PJ. The SLEEPER genes: A transposase-derived angiosperm-specific gene family. BMC Plant Biol. 2012;12:192. doi:10.1186/1471-2229-12-192.

99. Zorzatto C, Machado JPB, Lopes KVG, Nascimento KJT, Pereira WA, Brustolini OJB, et al. NIK1-mediated translation suppression functions as a plant antiviral immunity mechanism. Nature. 2015;520(7549):679-682. doi:10.1038/nature14171.

100. Yang Z, Wang C, Xue Y, Liu X, Chen S, Song C, et al. Calcium-activated 14-3-3 proteins as a molecular switch in salt stress tolerance. Nat Commun. 2019;10(1):1199. doi:10.1038/s41467-019-09181-2.

101. Yang Y, Moore MJ, Brockington SF, Mikenas J, Olivieri J, Walker JF, et al. Improved transcriptome sampling pinpoints 26 ancient and more recent polyploidy events in Caryophyllales, including two allopolyploidy events. New Phytol. 2018;217(2):855-870.

doi:10.1111/nph.14812.

102. Zhang L, Wu S, Chang X, Wang X, Zhao Y, Xia Y, et al. The ancient wave of polyploidization events in flowering plants and their facilitated adaptation to environmental stress. Plant Cell Environ. 2020;43(12):2847-2856. doi:10.1111/pce.13898.

103. Wang JP, Yu JG, Li J, Sun PC, Wang L, Yuan JQ, et al. Two likely auto-tetraploidization events shaped kiwifruit genome and contributed to establishment of the Actinidiaceae family. iScience. 2018;7:230-240. doi:10.1016/j.isci.2018.08.003.

104. Hanada K, Zou C, Lehti-Shiu MD, Shinozaki K and Shiu SH. Importance of lineage-specific expansion of plant tandem duplicates in the adaptive response to environmental stimuli. Plant Physiol. 2008;148(2):993-1003. doi:10.1104/pp.108.122457.

105. Hu X, Hao J, Pan L, Xu T, Ren L, Chen Y, et al. Genome-wide analysis of tandem duplicated genes and their expression under salt stress in seashore paspalum. Front Plant Sci. 2022;13:971999. doi:10.3389/fpls.2022.971999.

106. Huang Y-l, Zhang L-k, Zhang K, Chen S-m, Hu J-b and Cheng F. The impact of tandem duplication on gene evolution in Solanaceae species. J Integr Agric. 2022;21(4):1004-1014. doi:10.1016/S2095-3119(21)63698-5.

107. Price L, Han Y, Angessa T and Li C. Molecular pathways of WRKY genes in regulating plant salinity tolerance. Int J Mol Sci. 2022;23(18):10947. doi:10.3390/ijms231810947.

108. Golldack D, Lüking I and Yang O. Plant tolerance to drought and salinity: stress regulating transcription factors and their functional significance in the cellular transcriptional network. Plant Cell Rep. 2011;30(8):1383-1391. doi:10.1007/s00299-011-1068-0.

109. Jiang Y and Deyholos MK. Functional characterization of *Arabidopsis* NaCl-inducible WRKY25 and WRKY33 transcription factors in abiotic stresses. Plant Mol Biol. 2009;69(1-2):91-105. doi:10.1007/s11103-008-9408-3.

110. Krishnamurthy P, Vishal B, Ho WJ, Lok FCJ, Lee FSM and Kumar PP. Regulation of a cytochrome P450 gene CYP94B1 by WRKY33 transcription factor controls apoplastic barrier formation in roots to confer salt tolerance. Plant Physiol. 2020;184(4):2199-2215. doi:10.1104/pp.20.01054.

111. Wang H, Zheng Y, Xiao D, Li Y, Liu T and Hou X. BcWRKY33A enhances resistance to *Botrytis cinerea* via activating *BcMYB51-3* in non-heading Chinese cabbage. Int J Mol Sci. 2022;23(15):8222. doi:10.3390/ijms23158222.

112. Wang H, Li Z, Ren H, Zhang C, Xiao D, Li Y, et al. Regulatory interaction of BcWRKY33A and BcHSFA4A promotes salt tolerance in non-heading Chinese cabbage [*Brassica campestris* (syn. *Brassica rapa*) ssp. *chinensis*]. Hortic Res. 2022;9:uhac113. doi:10.1093/hr/uhac113.

113. Lin L, Yuan K, Huang Y, Dong H, Qiao Q, Xing C, et al. A WRKY transcription factor PbWRKY40 from *Pyrus betulaefolia* functions positively in salt tolerance and modulating organic acid accumulation by regulating *PbVHA-B1* expression. Environ Exp Bot. 2022;196:104782. doi:10.1016/j.envexpbot.2022.104782.

114. Dai W, Wang M, Gong X and Liu JH. The transcription factor FcWRKY40 of *Fortunella crassifolia* functions positively in salt tolerance through modulation of ion homeostasis and proline biosynthesis by directly regulating *SOS2* and *P5CS1* homologs. New Phytol. 2018;219(3):972-989. doi:10.1111/nph.15240.

115. Zhang M and Zhang S. Mitogen-activated protein kinase cascades in plant signaling. J Integr Plant Biol. 2022;64(2):301-341. doi:10.1111/jipb.13215.

116. Pitzschke A, Datta S and Persak H. Salt stress in *Arabidopsis*: Lipid transfer protein AZI1 and its control by mitogen-activated protein kinase MPK3. Mol Plant. 2014;7(4):722-738. doi:10.1093/mp/sst157.

117. Pitzschke A, Datta S and Persak H. Mitogen-activated protein kinase-regulated AZI1 - an attractive candidate for genetic engineering. Plant Signal Behav. 2014;9(2):e27764. doi:10.4161/psb.27764.

118. Yan Z, Wang J, Wang F, Xie C, Lv B, Yu Z, et al. MPK3/6-induced degradation of ARR1/10/12 promotes salt tolerance in *Arabidopsis*. EMBO Rep. 2021;22(10):e52457. doi:10.15252/embr.202152457.

119. Wang F, Jing W and Zhang W. The mitogen-activated protein kinase cascade MKK1-MPK4 mediates salt signaling in rice. Plant Sci. 2014;227:181-9. doi:10.1016/j.plantsci.2014.08.007.

120. Zhang LL, Shao YJ, Ding L, Wang MJ, Davis SJ and Liu JX. XBAT31 regulates thermoresponsive hypocotyl growth through mediating degradation of the thermosensor ELF3 in *Arabidopsis*. Sci Adv. 2021;7(19):eabf4427. doi:10.1126/sciadv.abf4427.

121. Senthil-Kumar M and Mysore KS. Ornithine-delta-aminotransferase and proline dehydrogenase genes play a role in non-host disease resistance by regulating pyrroline-5-carboxylate metabolism-induced hypersensitive response. Plant Cell Environ. 2012;35(7):1329-1343. doi:10.1111/j.1365-3040.2012.02492.x.

122. Chen H, Lai Z, Shi J, Xiao Y, Chen Z and Xu X. Roles of arabidopsis WRKY18, WRKY40 and WRKY60 transcription factors in plant responses to abscisic acid and abiotic stress. BMC Plant Biol. 2010;10:281. doi:10.1186/1471-2229-10-281.

123. Mittler R, Kim Y, Song L, Coutu J, Coutu A, Ciftci-Yilmaz S, et al. Gain- and loss-of-function mutations in Zat10 enhance the tolerance of plants to abiotic stress. FEBS Lett. 2006;580(28-29):6537-6542. doi:10.1016/j.febslet.2006.11.002.

124. Dang F, Li Y, Wang Y, Lin J, Du S and Liao X. *ZAT10* plays dual roles in cadmium uptake and detoxification in *Arabidopsis*. Front Plant Sci. 2022;13:994100.

doi:10.3389/fpls.2022.994100.

125. Tran LS, Urao T, Qin F, Maruyama K, Kakimoto T, Shinozaki K, et al. Functional analysis of AHK1/ATHK1 and cytokinin receptor histidine kinases in response to abscisic acid, drought, and salt stress in *Arabidopsis*. Proc Natl Acad Sci U S A. 2007;104(51):20623-20628. doi:10.1073/pnas.0706547105.

126. Yamada H, Suzuki T, Terada K, Takei K, Ishikawa K, Miwa K, et al. The *Arabidopsis* AHK4 histidine kinase is a cytokinin-binding receptor that transduces cytokinin signals across the membrane. Plant Cell Physiol. 2001;42(9):1017-1023. doi:10.1093/pcp/pce127.

127. Taylor NG, Howells RM, Huttly AK, Vickers K and Turner SR. Interactions among three distinct CesA proteins essential for cellulose synthesis. Proc Natl Acad Sci U S A. 2003;100(3):1450-1455. doi:10.1073/pnas.0337628100.

128. Lee C, O'Neill MA, Tsumuraya Y, Darvill AG and Ye ZH. The irregular xylem9 mutant is deficient in xylan xylosyltransferase activity. Plant Cell Physiol. 2007;48(11):1624-1634. doi:10.1093/pcp/pcm135.

129. Peña MJ, Zhong R, Zhou GK, Richardson EA, O'Neill MA, Darvill AG, et al. *Arabidopsis* irregular xylem8 and irregular xylem9: implications for the complexity of glucuronoxylan biosynthesis. Plant Cell. 2007;19(2):549-563. doi:10.1105/tpc.106.049320.

130. Taylor-Teeples M, Lin L, de Lucas M, Turco G, Toal TW, Gaudinier A, et al. An *Arabidopsis* gene regulatory network for secondary cell wall synthesis. Nature. 2015;517(7536):571-575. doi:10.1038/nature14099.

131. Kidwai M, Ahmad IZ and Chakrabarty D. Class III peroxidase: An indispensable enzyme for biotic/abiotic stress tolerance and a potent candidate for crop improvement. Plant Cell Rep. 2020;39(11):1381-1393. doi:10.1007/s00299-020-02588-y.

132. Tuteja N, Tran NQ, Dang HQ and Tuteja R. Plant MCM proteins: Role in DNA replication

and beyond. Plant Mol Biol. 2011;77(6):537-545. doi:10.1007/s11103-011-9836-3.

133. Francis NJ and Kingston RE. Mechanisms of transcriptional memory. Nat Rev Mol Cell Biol. 2001;2(6):409-421. doi:10.1038/35073039.

134 Liu JN; Fang H; Liang Q; Dong Y; Wang C; Yan L et al. Supporting data for "Genomic analyses provide insights into the evolution and salinity adaptation of halophyte Tamarix chinensis". GigaScience Database. 2023. http://doi.org/10.5524/102417

Tables & Figures

Table 1 Features of *Tamarix chinensis* genome assembly

Type	Parameter	Value
Assembly	Genome size (Gb)	1.324
	Chromosome-scale scaffolds (Gb)	1.317
	Total num. of scaffolds	63
	Total num. of chromosomes	12
	Scaffold N50 (Mb)	110.03
	Scaffold L50	6
	Total num. of contigs	342
	Contig N50 (Mb)	11.93
	Contig L50	45
	GC content of the genome (%)	36.7
	Complete BUSCOs	97.4%
	Quality value (QV)	39.03
Annotation	Repeat sequences (Gb)	0.979 (73.94%)
	Total num. of protein-coding genes	26,426
	Complete BUSCOs	93.9%

Average length of genes (bp)	1233.60
Average exons per gene	5.30
Annotated in Swiss-Port	15,422
Annotated in NCBI NR	22,348
Annotated in COG	21,796
Annotated in InterPro	23,468
Annotated in GO	14,064
Annotated in KEGG	15,393

Figure Legends

Figure 1 Genome evolution of *Tamarix chinensis*. **(a)** Genomic features of *T. chinensis*. a, Circular representation of the pseudo-chromosomes. b, GC content. c, LTR/Gypsy distribution. d, LTR/Copia distribution. e, repeat elements distribution. f, protein-coding gene frequency. g, distribution of non-coding RNAs. h, distribution of log 2 of gene expression levels. i, intra-genome collinear blocks. All distributions are displayed in a window size of 1 Mb. **(b)** Phylogenetic analysis of *T. chinensis* based on 959 one-to-one orthologous genes shared across 12 plant species, including 8 Caryophyllales species and three outgroups (*Vitis vinifera*, *Arabidopsis thaliana*, and *Oryza sativa*) by RAxML using GTRCAT module with 200 bootstrap replicates. The pie chart represents the number of gene family expansions and contractions. The black dot indicates the calibration point. The star and hexagon indicate whole-genome

triplication (WGT) and whole-genome duplication (WGD) events. The node label displays 95% highest probability density (HPD) of divergence ages. MRCA, most recent common ancestor. All the branches represent bootstrap values equal to 100, which are not shown in the figure. **(c)** Comparison of the number of genes among each significantly expanded gene families between *T. chinensis* and other examined plants.

Figure 2 Whole-genome duplication event and gene duplications. **(a)** The synonymous substitution rates (K_s) distribution for paralog gene pairs of *T. chinensis* and other plant species, including 8 Caryophyllales species and two outgroups (*Vitis vinifera* and *Arabidopsis thaliana*). **(b)** The inter-species synthetic depths between *V. vinifera* and *T. chinensis*. **(c)** Macrosynteny between *V. vinifer* and *T. chinensis* karyotypes. The sky-blue line represents the three copies of *V. vinifer* syntenic blocks per *T. chinensis*. The red line indicates the two copies of *T. chinensis* syntenic blocks per *V. vinifer*. **(d)** K_s distribution for paralog gene pairs from *T. chinensis* using WGD software. The K_s distribution was subjected to the BGMM module in WGD for mixed model fitting, resulting in the hypothesized WGD peaks. Afterward, the average and variance of each WGD peak were estimated, and the paralog gene pairs of each WGD peak with 95% probability were extracted. The blue dash curve represents the WGD peak with K_s ranging from 0.35 to 1.21 (mean 0.61). **(e)** The K_a/K_s ratios of the five types of duplications. DSD, dispersed duplications. PD, proximal duplications. TD, tandem duplications. TRD, transposed duplications. **(f)** The K_s distribution of the five types of duplications. **(g)** Venn diagram shows the number of shared and specific gene duplications between the expanded genes (EPGs) and five types of duplications. WGDps, the WGD duplications underwent positive selection. TDps, the tandem duplications underwent positive selection. PDps, proximal duplications underwent positive selection. TRDps, the transposed duplications underwent positive selection. DSDps,

the dispersed duplications underwent positive selection. **(h)** Gene ontology (GO) category enrichment analyses on the shared EPGs of five types of gene duplications.

Figure 3 The syntenic relationships of the whole-genome duplications involved in salt stress sensing and ion homeostasis in *Tamarix chinensis*. The red line represents the major gene pairs with the syntenic relationships. AKT1, potassium channel AKT1. CLC, chloride channel protein CLC. CNGC, cyclic nucleotide- and calmodulin-regulated ion channel. GLR, glutamate receptor (ligand-gated ion channel). GRF, 14-3-3-like protein (General regulatory factor). HKT, sodium transporter HKT. KEA, K^+ efflux antiporter. NHX, sodium/hydrogen exchanger. SKOR, stelar K^+ outward rectifying channel. TPK, two-pore potassium channel (calcium-activated outward-rectifying potassium channel).

Figure 4 Experimental set-up and transcriptome analysis. (**a**) Overview of RNA-seq experimental design. Before imposing NaCl stress, the cutting clones were pre-treated in a medium containing 200 mM NaCl for two hours to avoid salt shock. The cutting clones were grown for seven days on a hydroponic medium with 300 mM NaCl before transferring to the hydroponic medium without NaCl for 35 d to recovery. Seven-time points for sample collection were selected to cover early salt stress (including 300 mM NaCl stressed 0.5, 3, 5, and 8 h) and late salt stress and recovery (including 300 mM NaCl stressed 7 d and 35 d of recovery). Subsequently, 54 samples covering the seven-time points with three biological replicates per condition were harvested. (**b**) Principal coordinates analysis of gene expressions revealed that root and shoot showed distinct gene expression profiles; peculiarly, the early salt stress, and late stress and recovery treatments on roots, also exhibited distinct gene expression profiles. (**c**) Statistics of the differentially expressed genes were generated from four differential analysis

methods, including DESeq2, edgeR, ROTS, and Limma. (**d**) The Pearson correlation coefficient between the qRT-PCR and RNA-seq results. The analysis was conducted using Graphpad Prism 9.

Figure 5 Identification of hub genes responding to early salt stress. **(a, b)** Upset plots of the number of differentially expressed genes (DEGs) identified in the root (**a**) and shoot (**b**) during early salt stress. (**c**) Gene ontology (GO) category enrichment analyses on the shared upregulated or downregulated DEGs among the time-points salt exposure in the root (0.5, 3, 5, and 8 h salt exposure) and shoot (5 and 8 h salt exposure). (**d**) Kyoto Encyclopedia of Genes and Genomes (KEGG) enrichment analyses were performed on the shared upregulated or downregulated DEGs among the time points in the root and shoot during early salt exposure. **(e, f)** The hub-ranked genes were identified in the root **(e)** and shoot **(f)**. Nodes colored from red to yellow represent degree ranking.

Figure 6 Identification of hub genes that respond to late salt stress and recovery. (a) Gene dynamics analysis of 1705 differentially expressed genes (DEGs) with opposite trends between stress and recovery or maintained at the recovery stage in the root. **(b)** Gene dynamics analysis of 612 DEGs with opposite trends between stress and recovery or maintained at the recovery stage in the shoot. **(c, d)** Gene ontology (GO) category enrichment analyses on the DEGs in the four major groups in the root **(c)** and shoot **(d)**. **(e, f)** The hub-ranked genes were identified in the root **(e)** and shoot **(f)** groups. Nodes colored from red to yellow represent degree ranking.

Figure
Click here to access/download;Figure;Figure 1.pdf

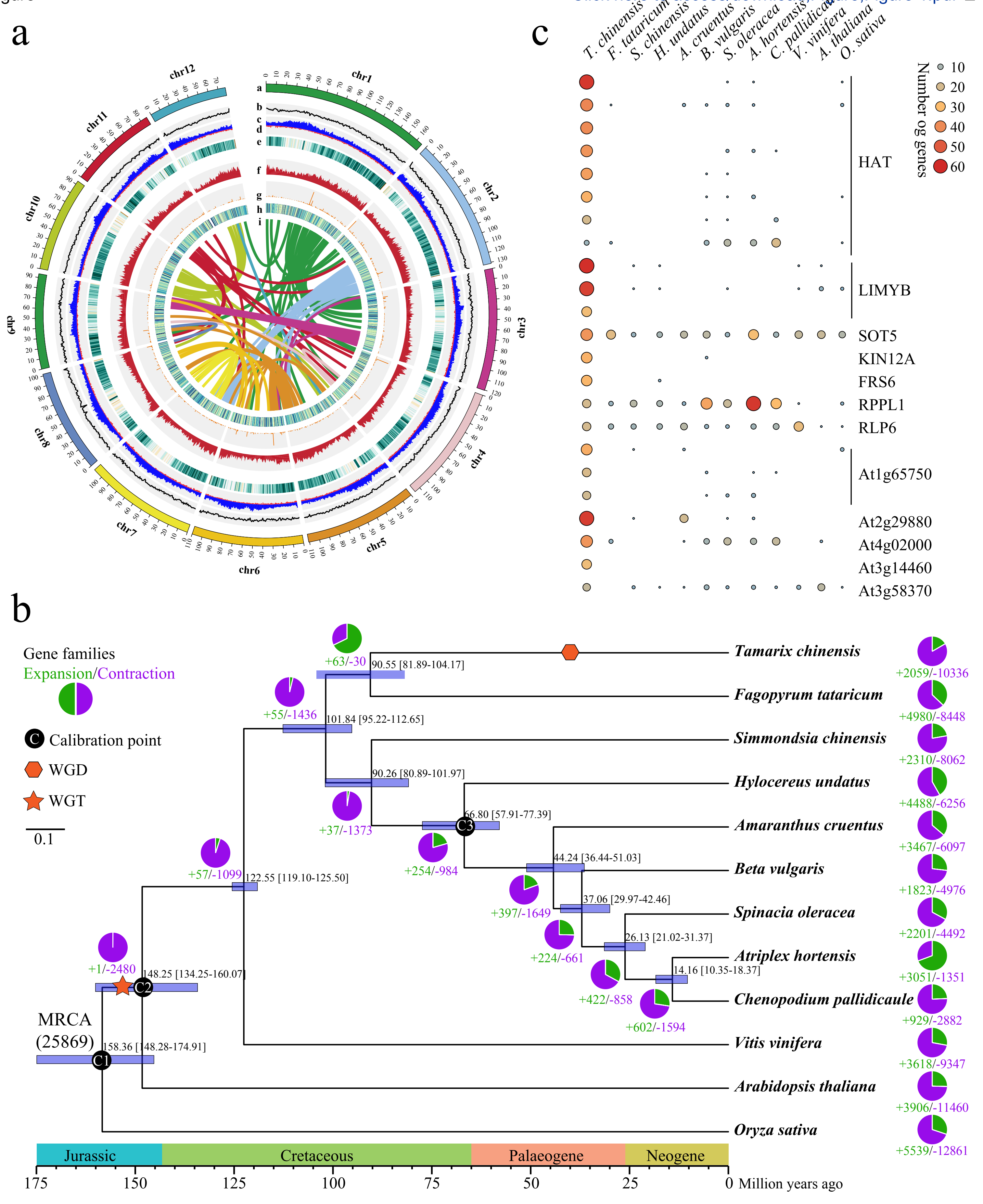

Figure
Click here to access/download;Figure;Figure 2.pdf

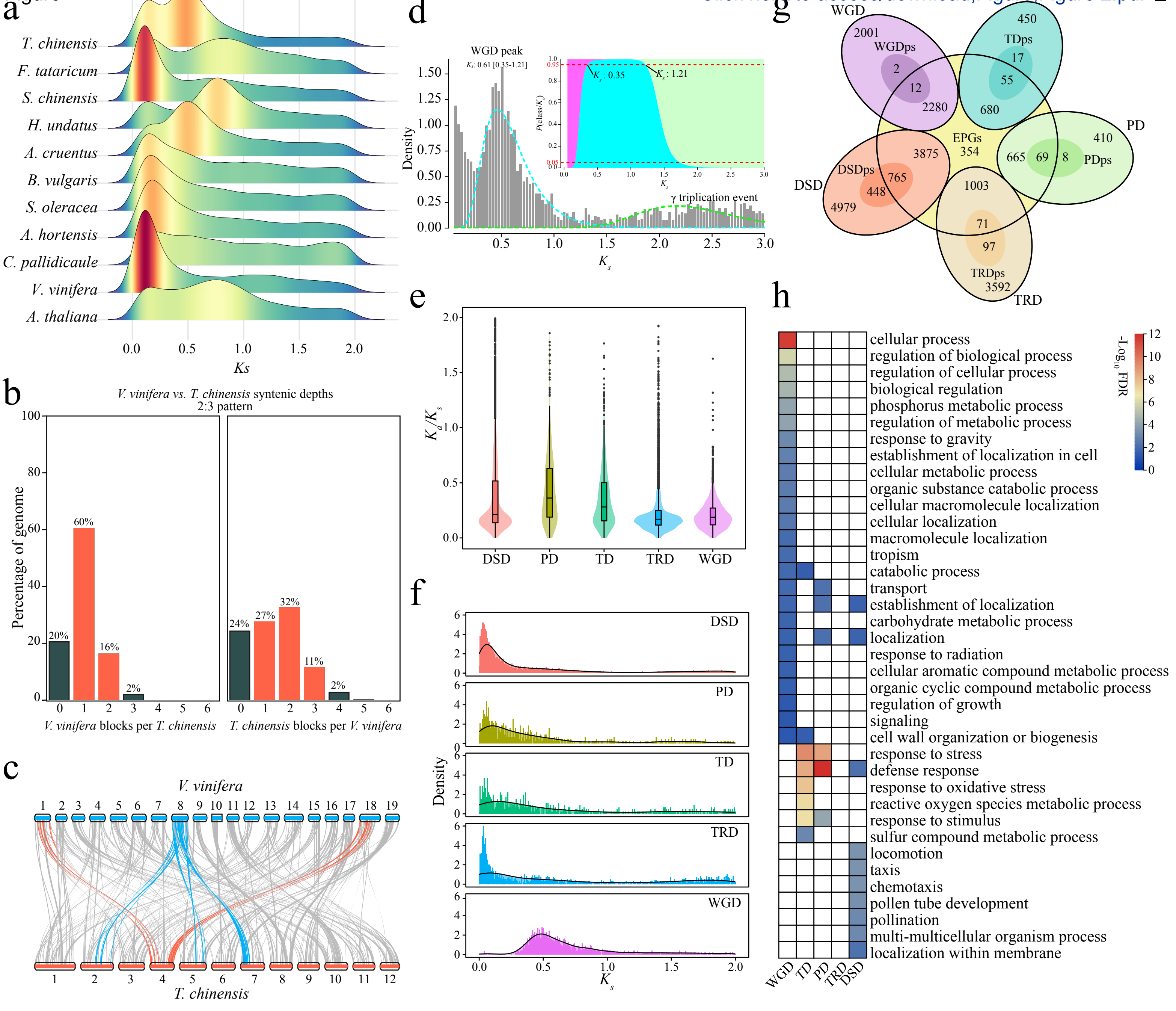

Figure
Click here to access/download;Figure;Figure 3.pdf

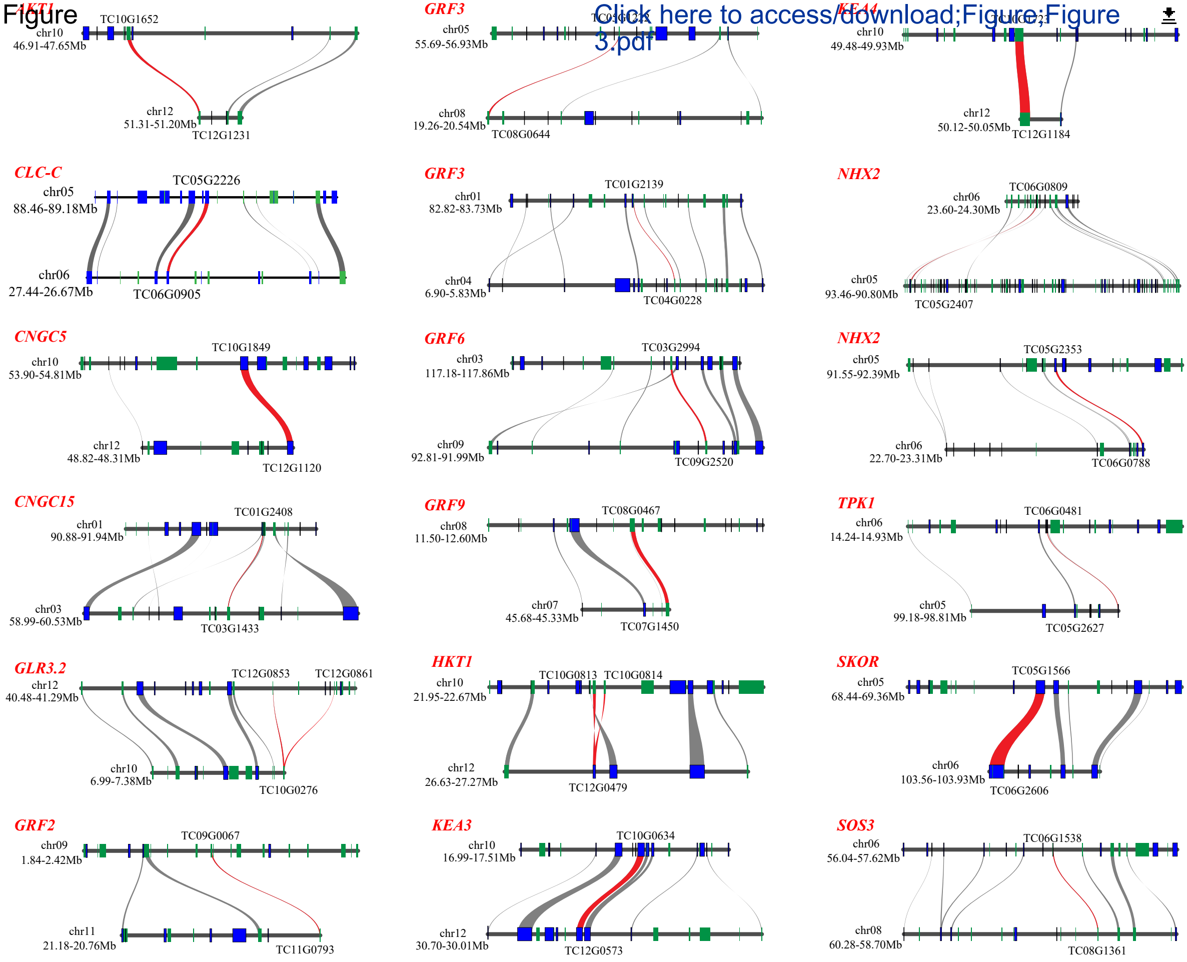

Figure

Click here to access/download;Figure;Figure 4.pdf

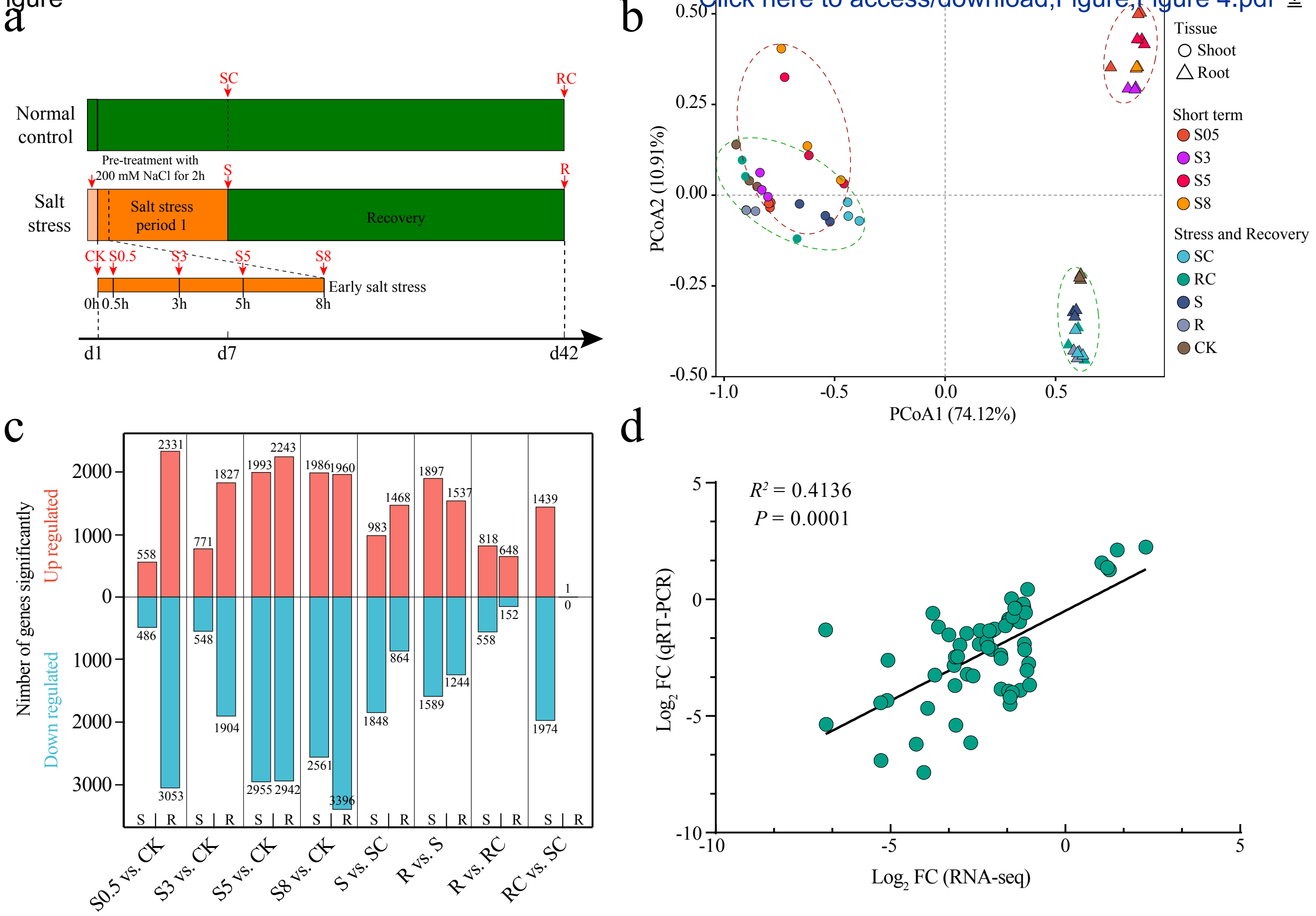

Figure

Click here to access/download;Figure;Figure 5.pdf

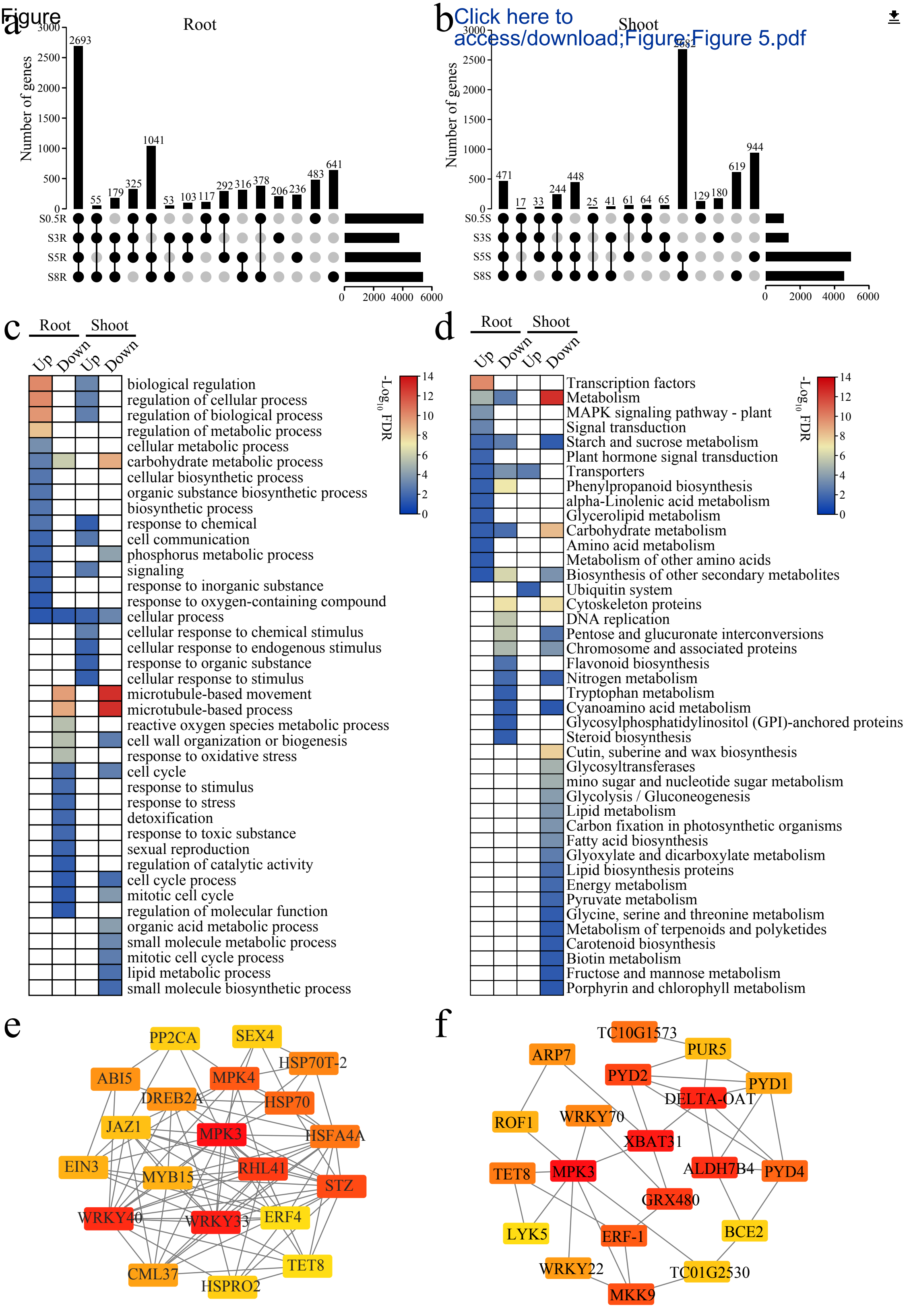

Figure Click here to access/download;Figure;Figure 6.pdf

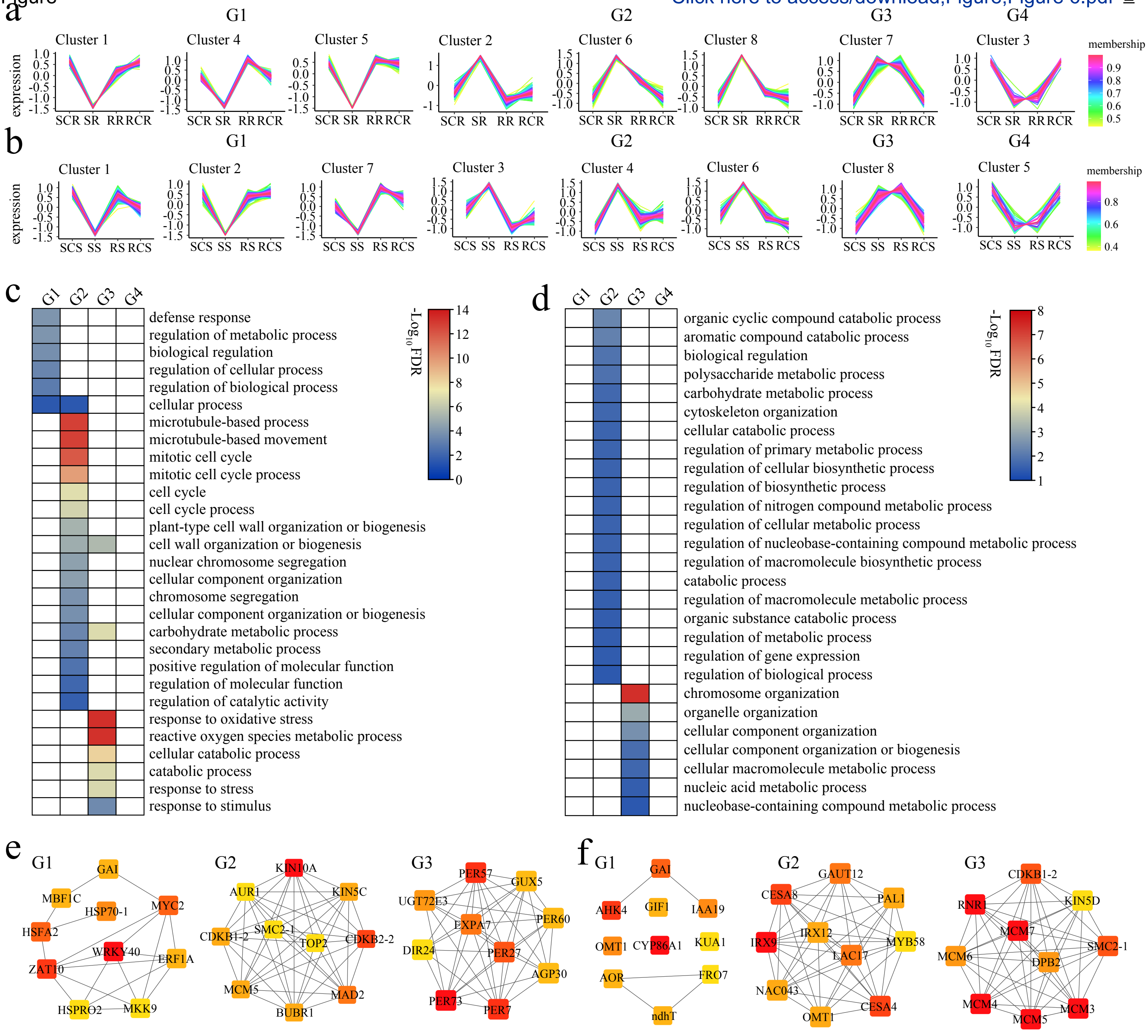

Click here to access/download
Supplementary Material
2023-6-9 Supplemental Information.docx

Click here to access/download
Supplementary Material
Supplementary_tables.xlsx
