# Supplementary material for: Genomic analyses provide insights into the evolution and salinity adaptation of halophyte Tamarix chinensis
Source: Gigascience. 2023 Jul 26;12:giad053. doi: 10.1093/gigascience/giad053 (PMC10370455; doi:10.1093/gigascience/giad053)
Supplement: giad053_Supplemental_Files [file giad053_supplemental_files.zip › 2023-6-9 Supplemental Information.docx]

**Supplemental Information**

**Genomic analyses provide insights into the evolution and** **salinity adaptation of halophyte *Tamarix chinensis***

Jian Ning Liu^1^, Hongcheng Fang^1,2,3^, Qiang Liang^1,2,3^, Yuhui Dong^1,2,3^, Changxi Wang^1^, Liping Yan^4^, Xinmei Ma^1^, Rui Zhou^1^, Xinya Lang^1^, Shasha Gai^1^, Lichang Wang^1^, Shengyi Xu^1^, Ke Qiang Yang^1,2,3,^ *, Dejun Wu^4,^ *

^1^ College of Forestry, Shandong Agricultural University, Taian 271018, China

^2^ State Forestry and Grassland Administration Key Laboratory of Silviculture in the Downstream Areas of the Yellow River, Shandong Agricultural University, Taian 271018, China

^3^ Shandong Taishan Forest Ecosystem Research Station, Shandong Agricultural University, Taian 271018, China

^4^ Shandong Provincial Academy of Forestry, Jinan 250014, China

*Corresponding author: Ke Qiang Yang: yangwere@126.com and Dejun Wu: sdlky412x@163.com

**Supplemental tables**

**Table S1** The statistics of PacBio long reads and Hi-C data

**Table S2** The quality of genome assembly evaluated by BUSCO

**Table S3** Identification of repetitive elements in the genome assembly

**Table S4** The quality of gene predictions evaluated by BUSCO

**Table S5** Detailed data used for phylogenetic analysis

**Table S6** The species used for comparative genomic analysis

**Table S7** Statistics of orthogroups per species

**Table S8** Statistics of gene family expansions and contractions among 12 examined plant species

**Table S9** Function annotation of the significant gene family expansions

**Table S10** Statistics of gene duplications

**Table S11** The WGD duplications involved in salt stress sensing and ion homeostasis balance

**Table S12** Statistics of RNA sequencing data

**Table S13** Statistics of differentially expressed genes identified by four differential analysis methods, including DESeq2, edgeR, ROTS, and Limma

**Table S14** The differentially expressed genes identified in the root during early salt stress

**Table S15** The differentially expressed genes identified in the shoot during early salt stress

**Table S16** The differentially expressed genes identified in the root during late salt stress and recovery

**Table S17** The differentially expressed genes identified in the shoot during late salt stress and recovery

**Table S18** The PCR primers used in the present study

**Table S19** The differentially expressed genes identified in the root shared among 0.5, 3, 5, and 8 h salt exposure

**Table S20** The differentially expressed genes identified in the shoot shared by 5 and 8 h salt exposure

**Table S21** Protein-protein interactions regarding the shared upregulated differentially expressed genes identified in the root

**Table S22** Protein-protein interactions regarding the shared upregulated differentially expressed genes identified in the shoot

**Table S23** The differentially expressed genes identified with opposite trends between stress and recovery or maintained at the recovery stage in the root during late salt stress and recovery

**Table S24** Protein-protein interactions of the G1 genes identified in the root

**Table S25** Protein-protein interactions of the G2 genes identified in the root

**Table S26** Protein-protein interactions of the G3 genes identified in the root

**Table S27** The differentially expressed genes identified with opposite trends between stress and recovery or maintained at the recovery stage in the shoot during late salt stress and recovery

**Table S28** Protein-protein interactions of the G1 genes identified in the shoot

**Table S29** Protein-protein interactions of the G2 genes identified in the shoot

**Table S30** Protein-protein interactions of the G3 genes identified in the shoot

**Supplemental figures**

**
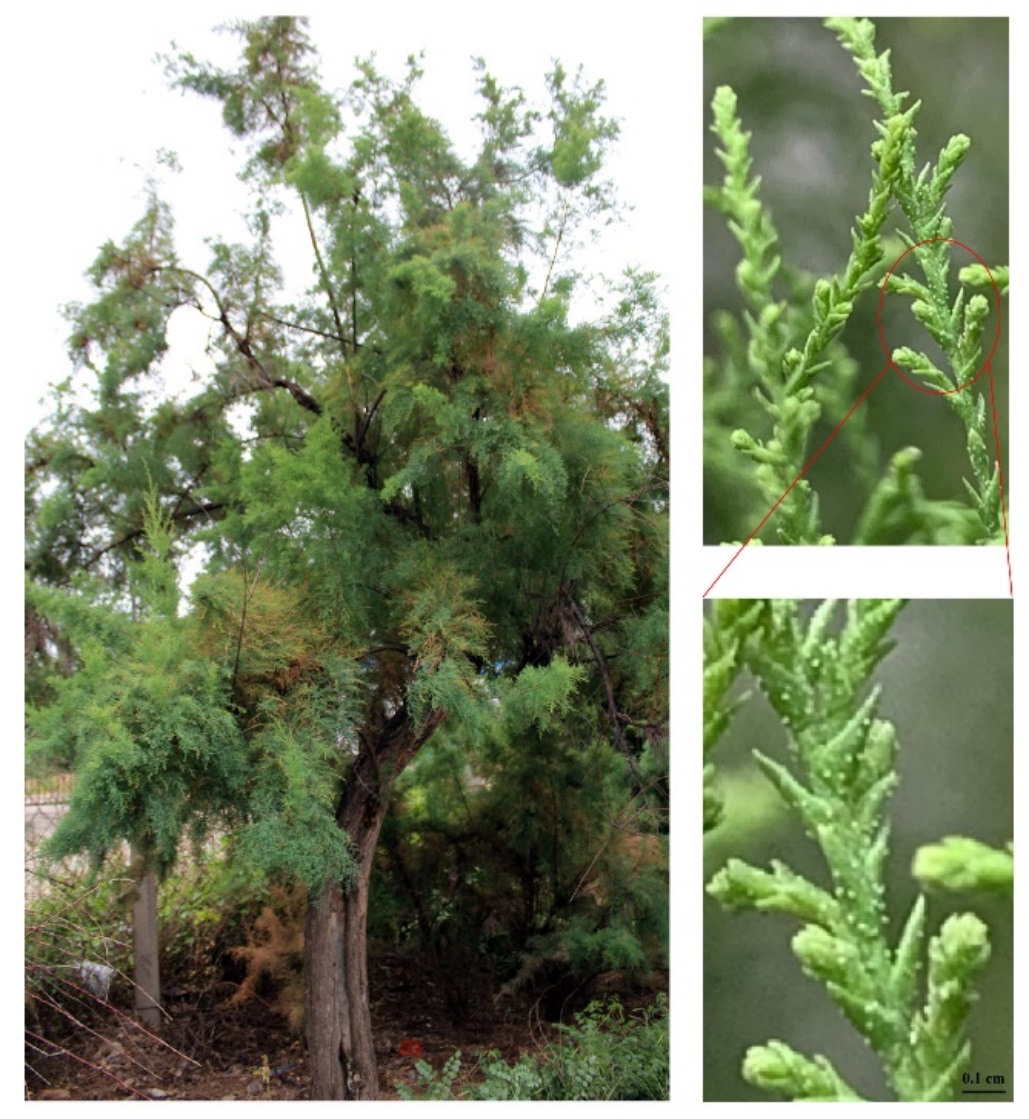
**

**Figure S1** The photo of the diploid *Tamarix chinensis* Lour. ‘Lucheng No.1’ (2n = 24) and the salt glands of *T. chinensis* Lour. ‘Lucheng No.1’ under salt stress.

**
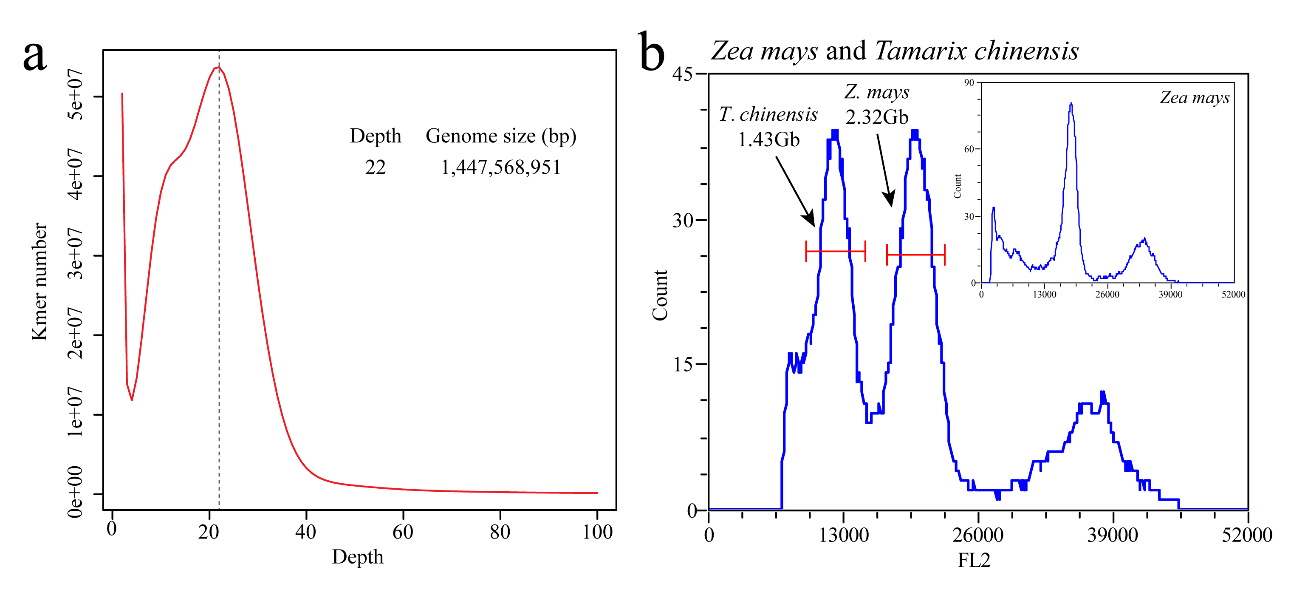
**

**Figure S2** Genome size estimation of *Tamarix chinensis* ‘Lucheng No.1’. **(a)** The size of *T. chinensis* ‘Lucheng No.1’ genome was estimated by 30-mer frequency analysis, resulting in a depth of 22 for the highest peak. The size of the *T. chinensis* ‘Lucheng No.1’ genome was determined by genome size = number of k-mer /average k-mer depth. **(b)** The size of *T. chinensis* ‘Lucheng No.1’ was evaluated using flow cytometry by comparing it to the genome size of *Zea may* ‘B73’ of approximately 2.32 Gb.


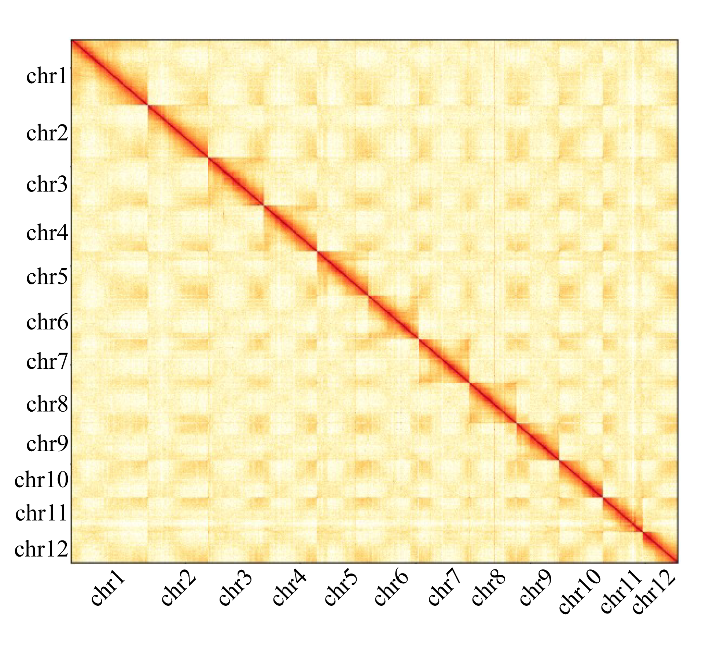


**Figure S3** The Hi-C contact maps of *Tamarix chinensis* genome assembly. The maps were visualized using HiCPlotter.

**
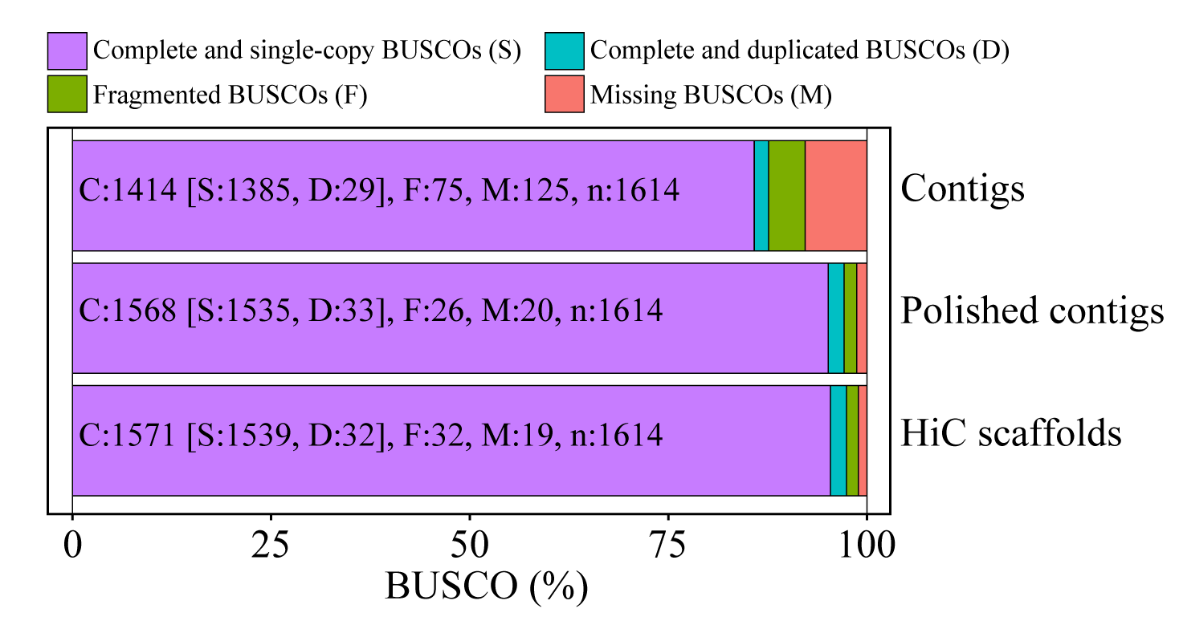
**

**Figure S4** Bar chart showing the quality of genome assembly assessed by BUSCO.


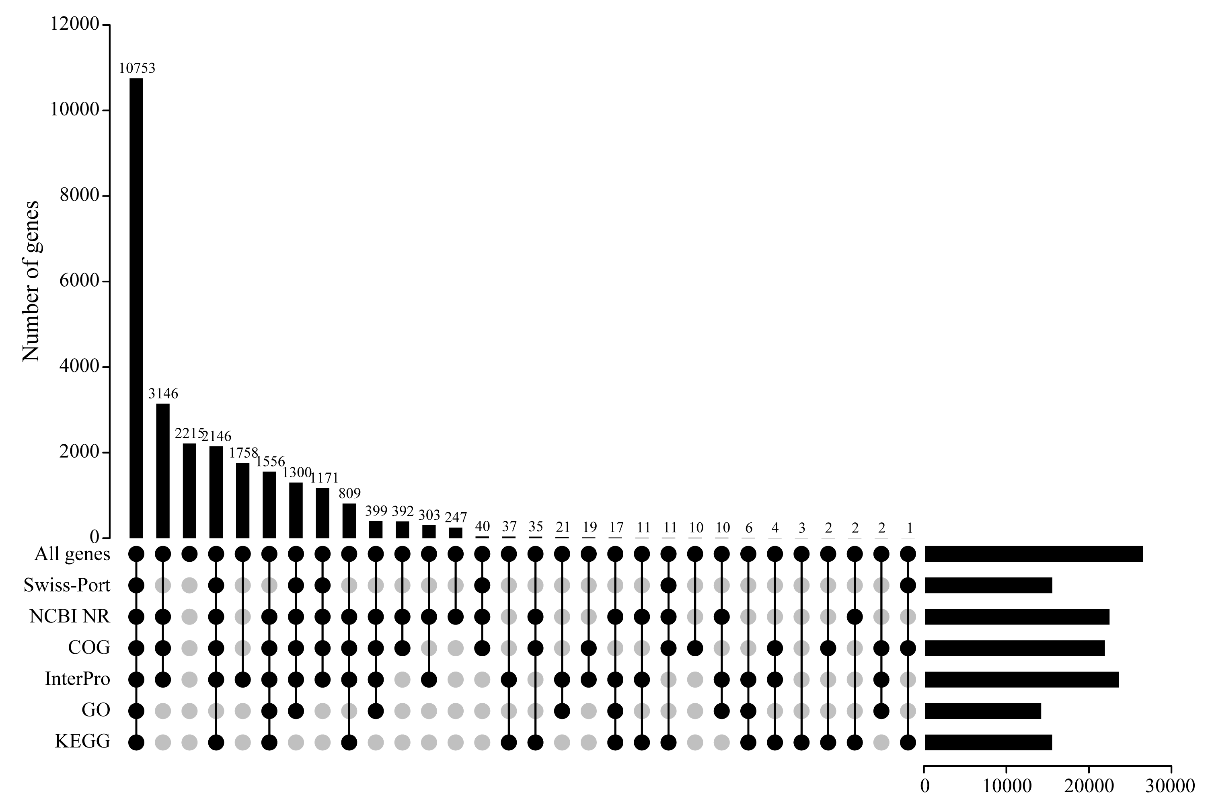


**Figure S****5** Upset plots of the number of functionally annotated genes in the *Tamarix chinensis* genome.


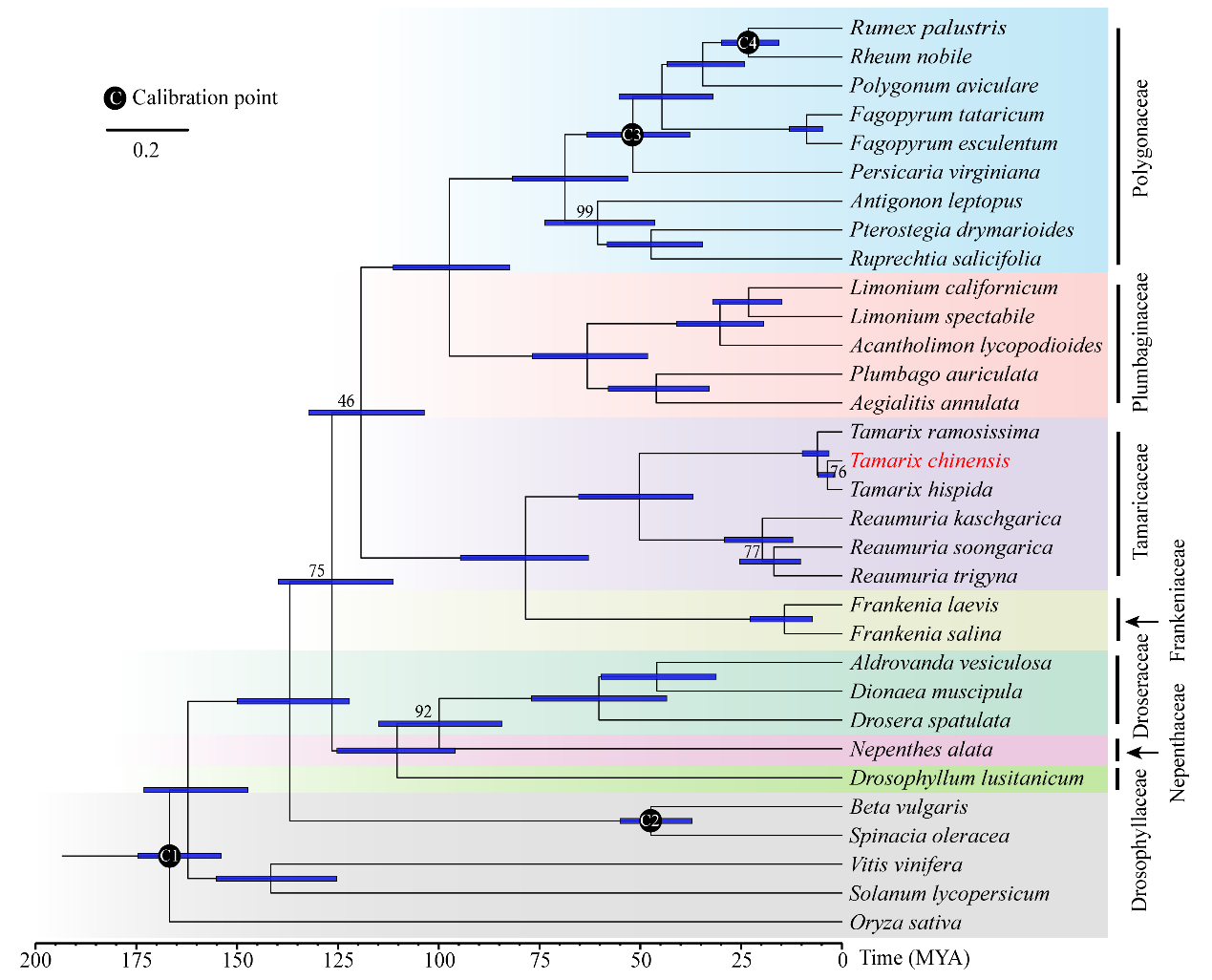


**Figure S6** Phylogenetic analysis of *Tamarix chinensis* based on 33 one-to-one orthologous genes shared across 32 angiosperm species using the modified phylome approach. The number above each branch represents the bootstrap value (a bootstrap value equal to 100 is not showing). The black dot indicates the calibration point.


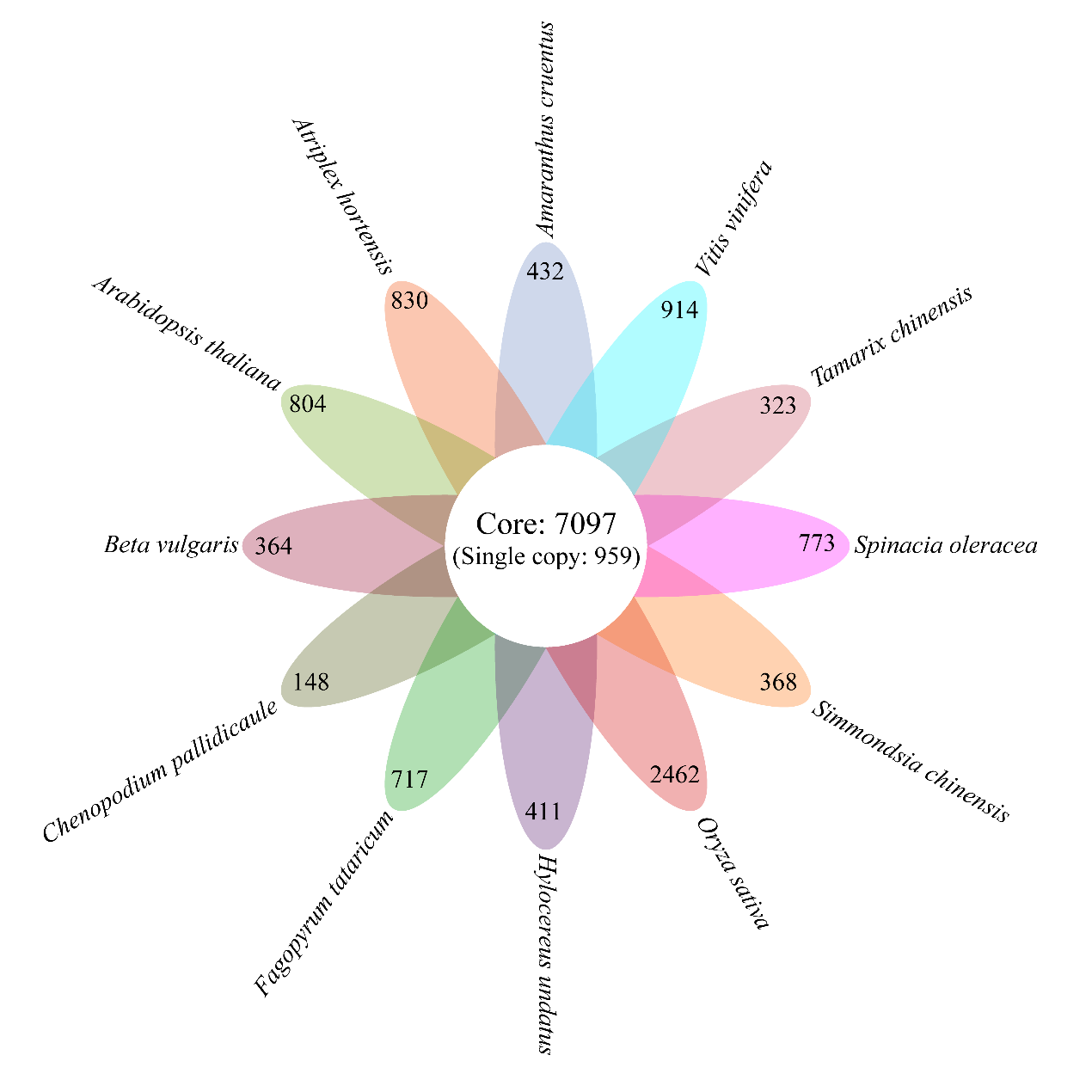


**Figure S7** Venn diagram shows the number of shared and species-specific gene families among *Tamarix chinensis* and other 11 plants.


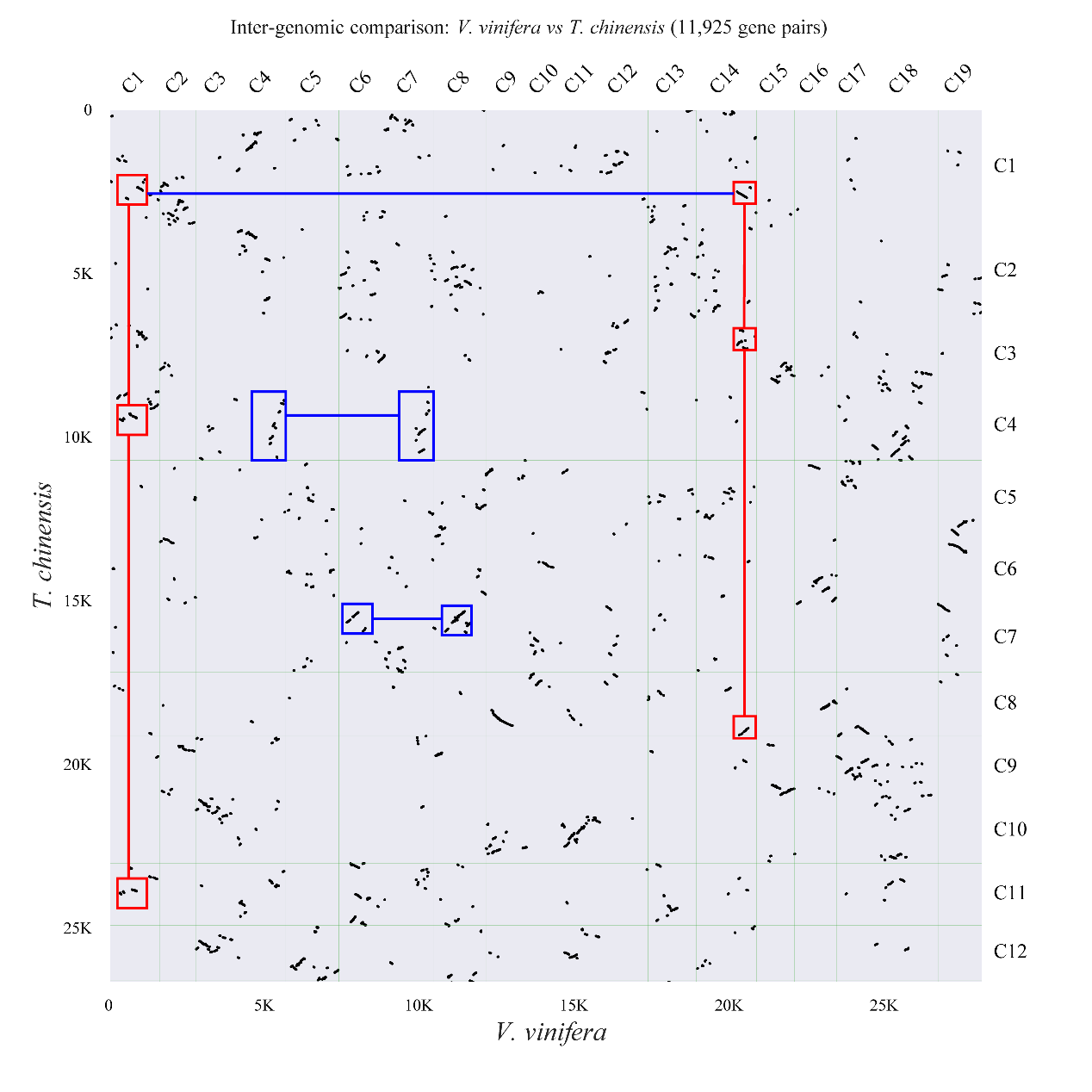


**Figure S8** Dot plot displays the comparative analysis of *Tamarix chinensis* and *Vitis vinifera* genomes. The red and blue squares indicate some main duplication events, and the dots exhibit synteny gene pairs.


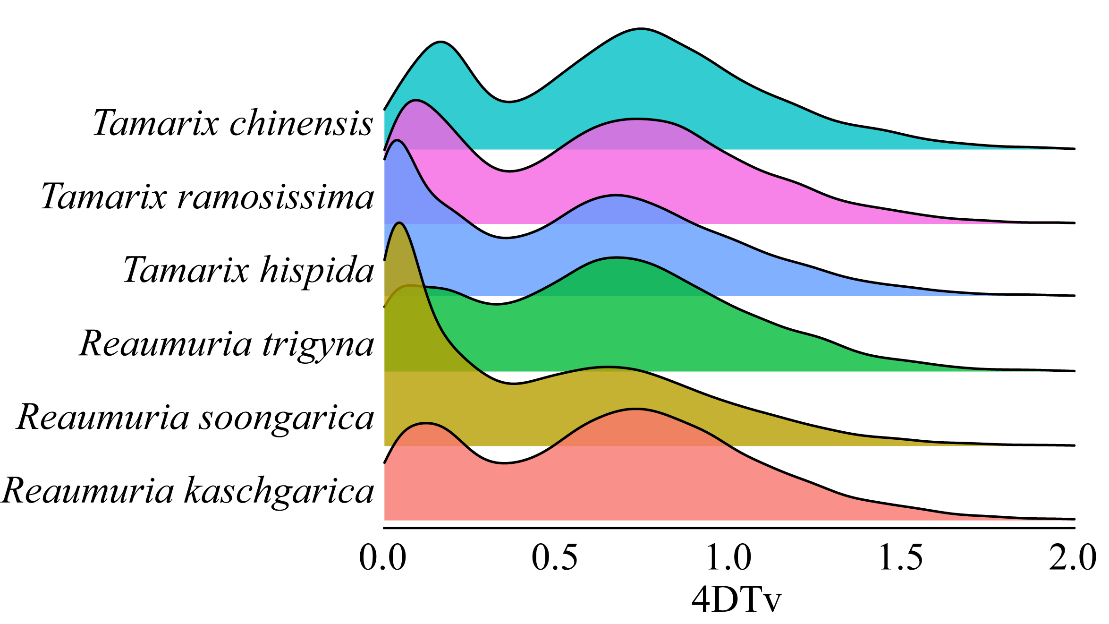


**Figure S9** The 4DTv (fourfold degenerate synonymous sites) distribution of Tamaricaceae species.


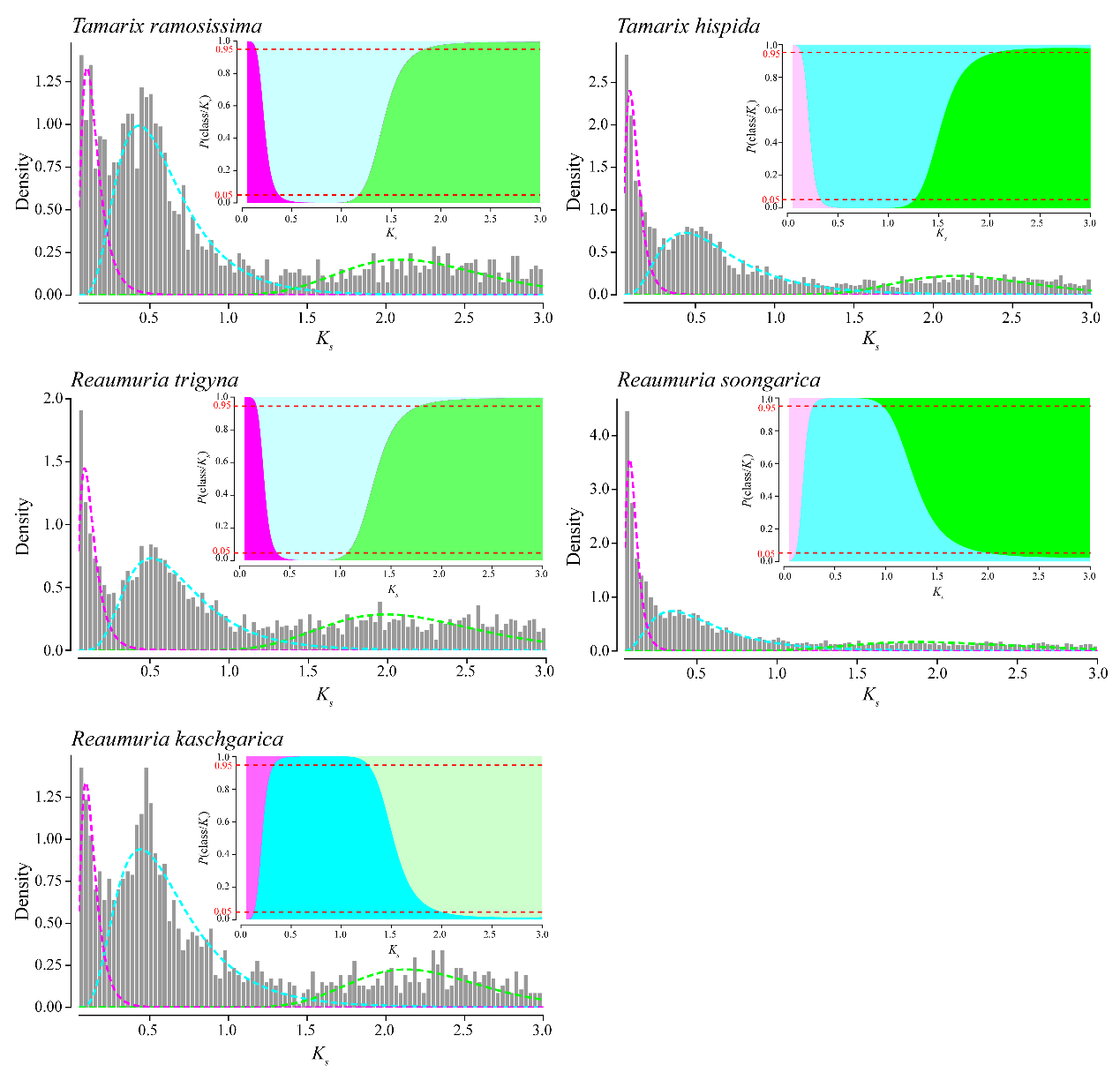


**Figure S10** *K_s_* (synonymous substitution rate) distribution for paralog gene pairs in five Tamaricaceae species, including *Tamarix ramosissima*, *T. hispida*, *Reaumuria kaschgarica*, *R. soongarica*, and *R. trigyna*.


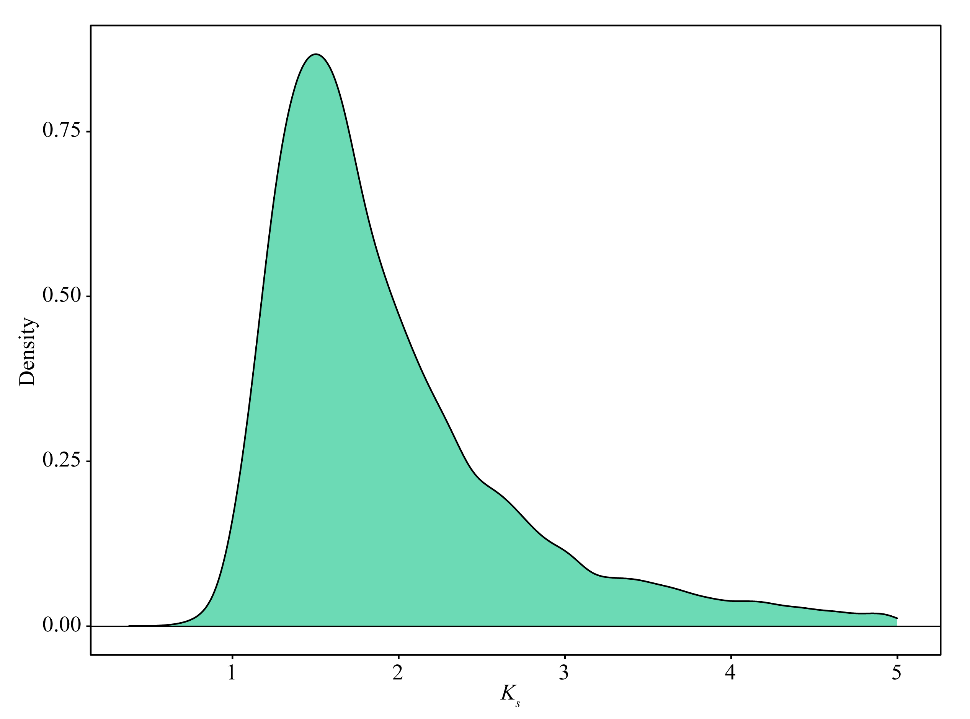


**Figure S11** *K_s_* (synonymous substitution rate) distribution for *Tamarix chinensis* ortholog genes with *Vitis vinifer.* Based on divergence time (115 Ma ago) and mean peak *K_s_* values (1.754) of orthologous genes of syntenic blocks between *T. chinensis* and *V. vinifera*, the synonymous nucleotide substitutions rate of Tamaricaceae was estimated to be 7.62×10^-9^ substitutions per site per year. According to divergence date = *K_s_* / 2r, the age of *T. chinensis* WGDs was calculated.


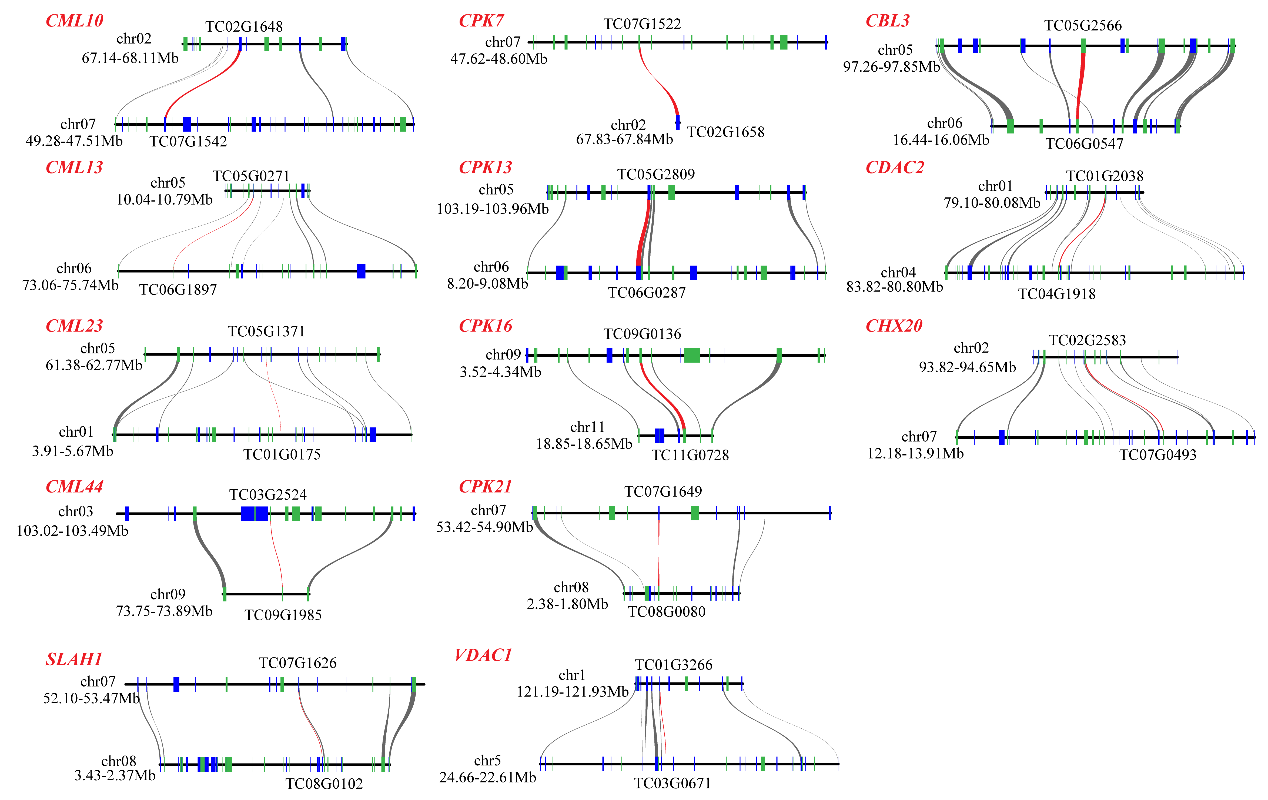


**Figure S12** The syntenic relationships of the whole-genome duplications involved in salt stress sensing and ion homeostasis balance in *Tamarix chinensis*. The red line represents the major gene pairs with the syntenic relationships.


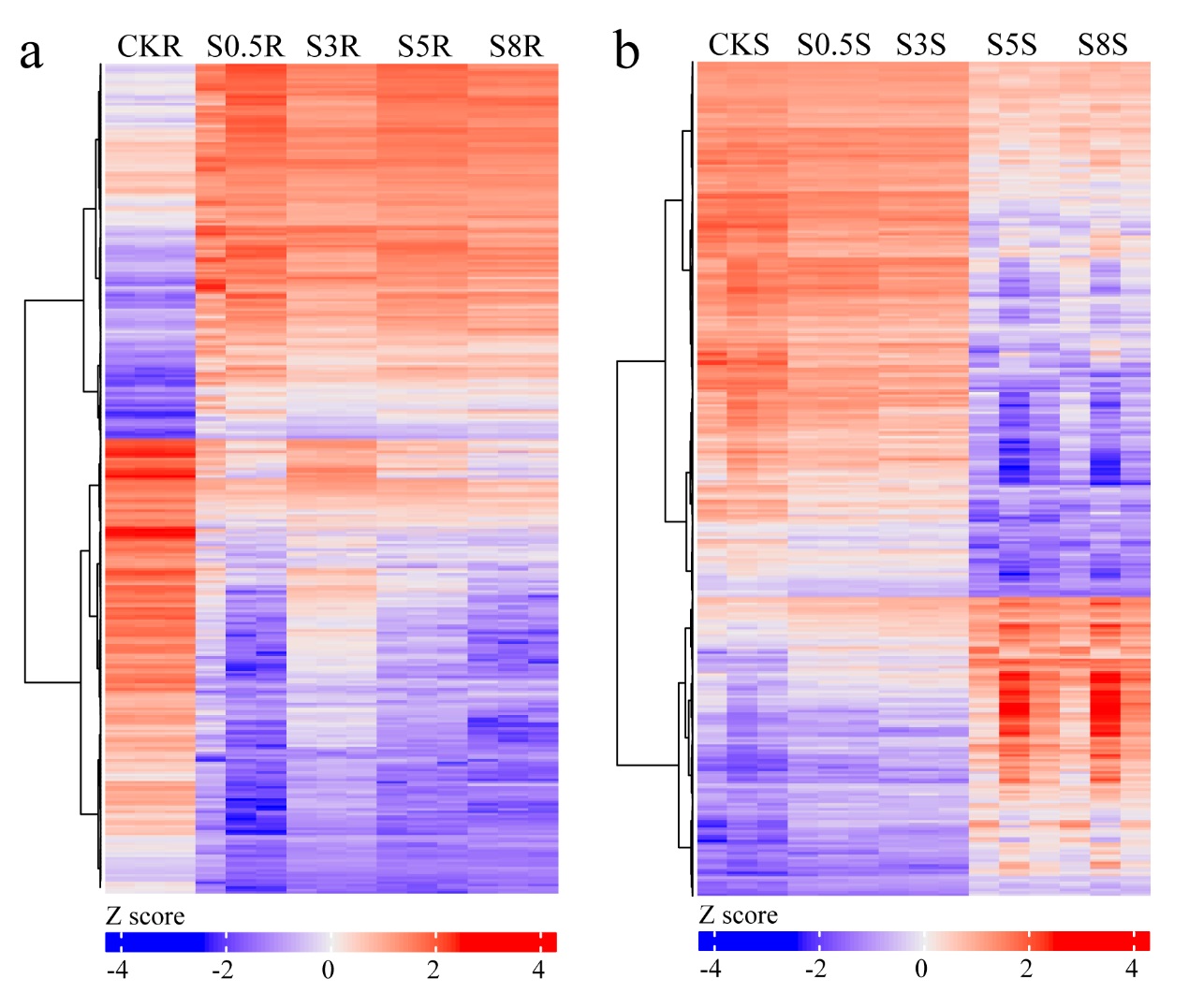


**Figure S13** Heatmap showing the expression of differentially expressed genes identified in the root **(a)** and shoot **(b)** responding to early salt stress.


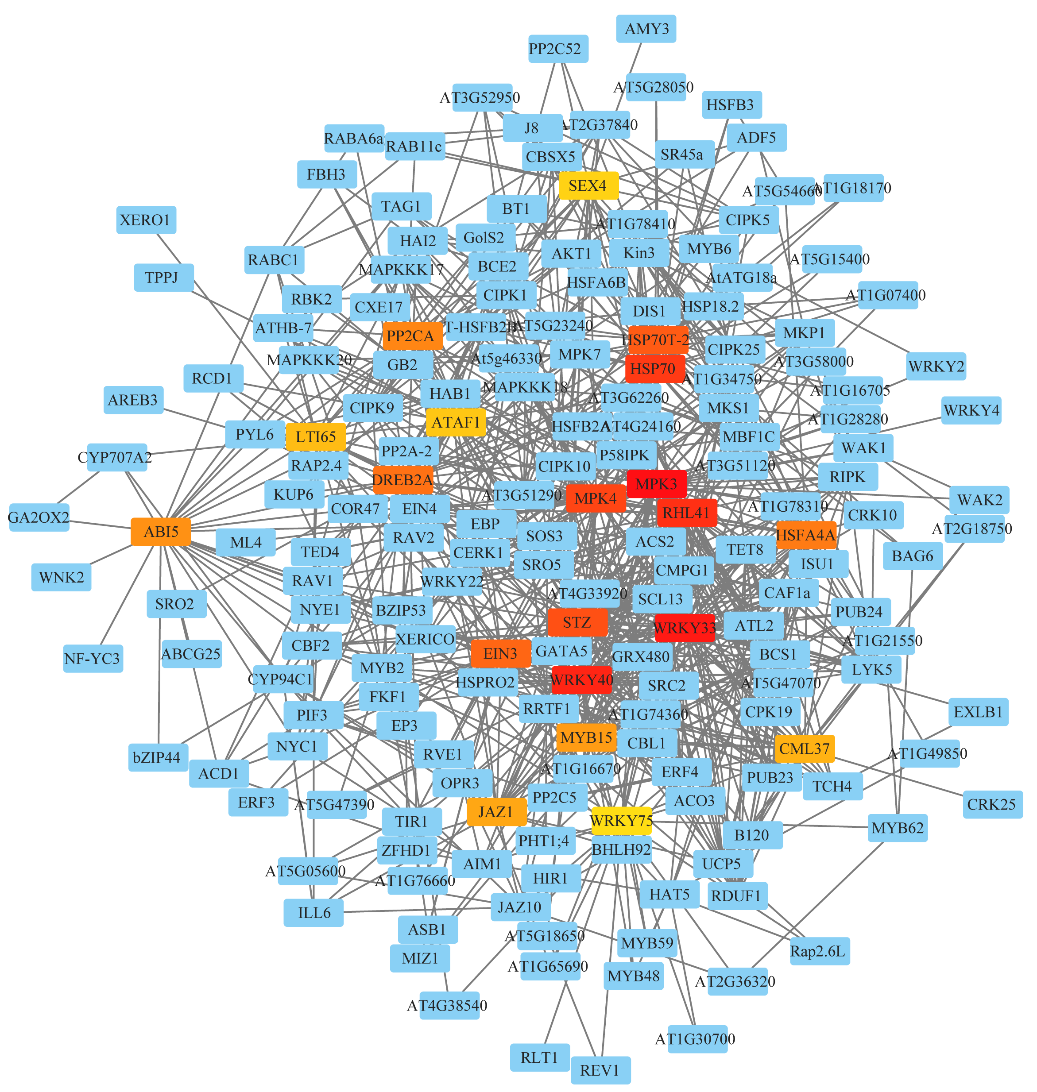


**Figure S14** A protein-protein interaction network containing 1711 interactions composed of 534 nodes and 1711 edges was constructed in the root exposed to early salt stress. Nodes colored red and yellow represent the hub genes. Color from red to yellow represents degree ranking.


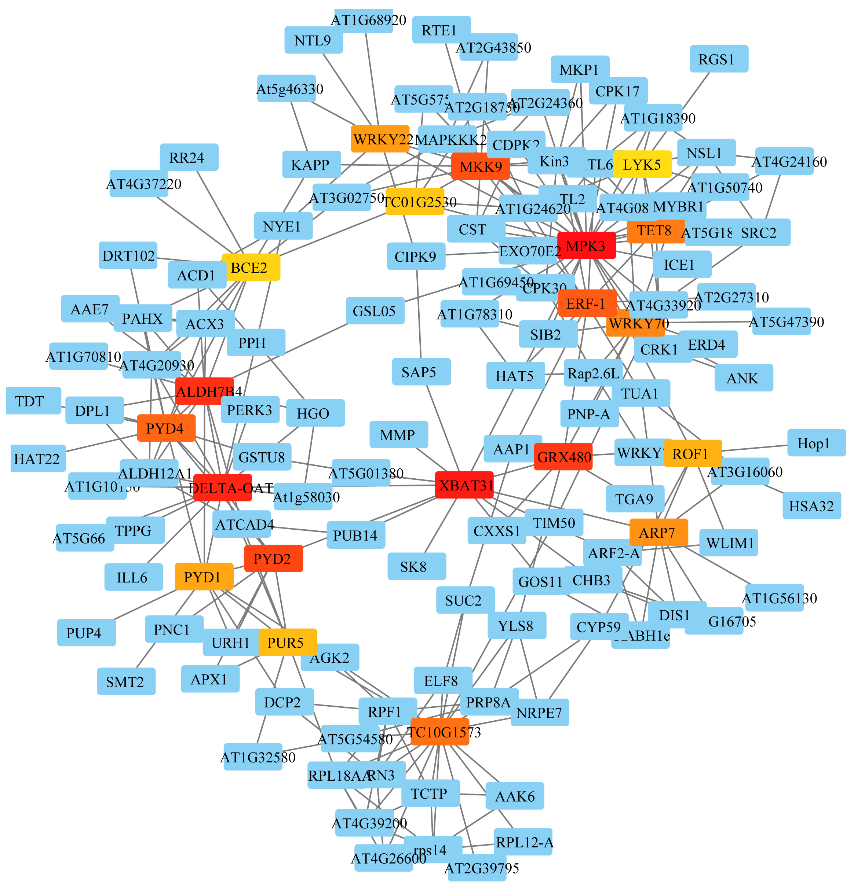


**Figure S15** A protein-protein interaction network containing 689 interactions composed of 400 nodes and 689 edges was constructed in the shoot exposed to short-term stress. Nodes colored red and yellow represent the hub genes. Color from red to yellow represents degree ranking.


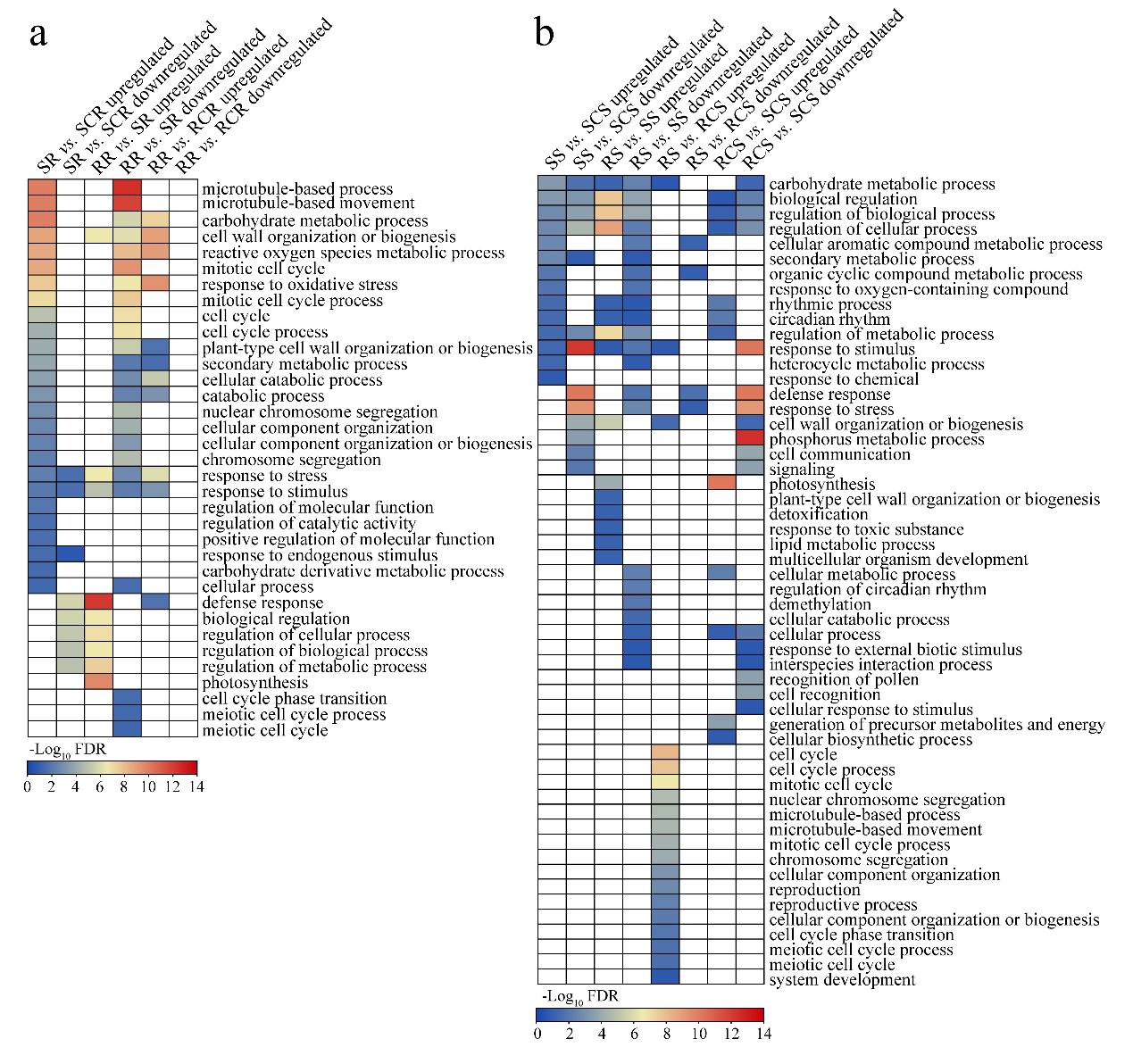


**Figure S16** Gene ontology category enrichment analysis of the upregulated and downregulated DEGs of each comparison in the root (**a**) and shoot (**b**) during salt stress and recovery.


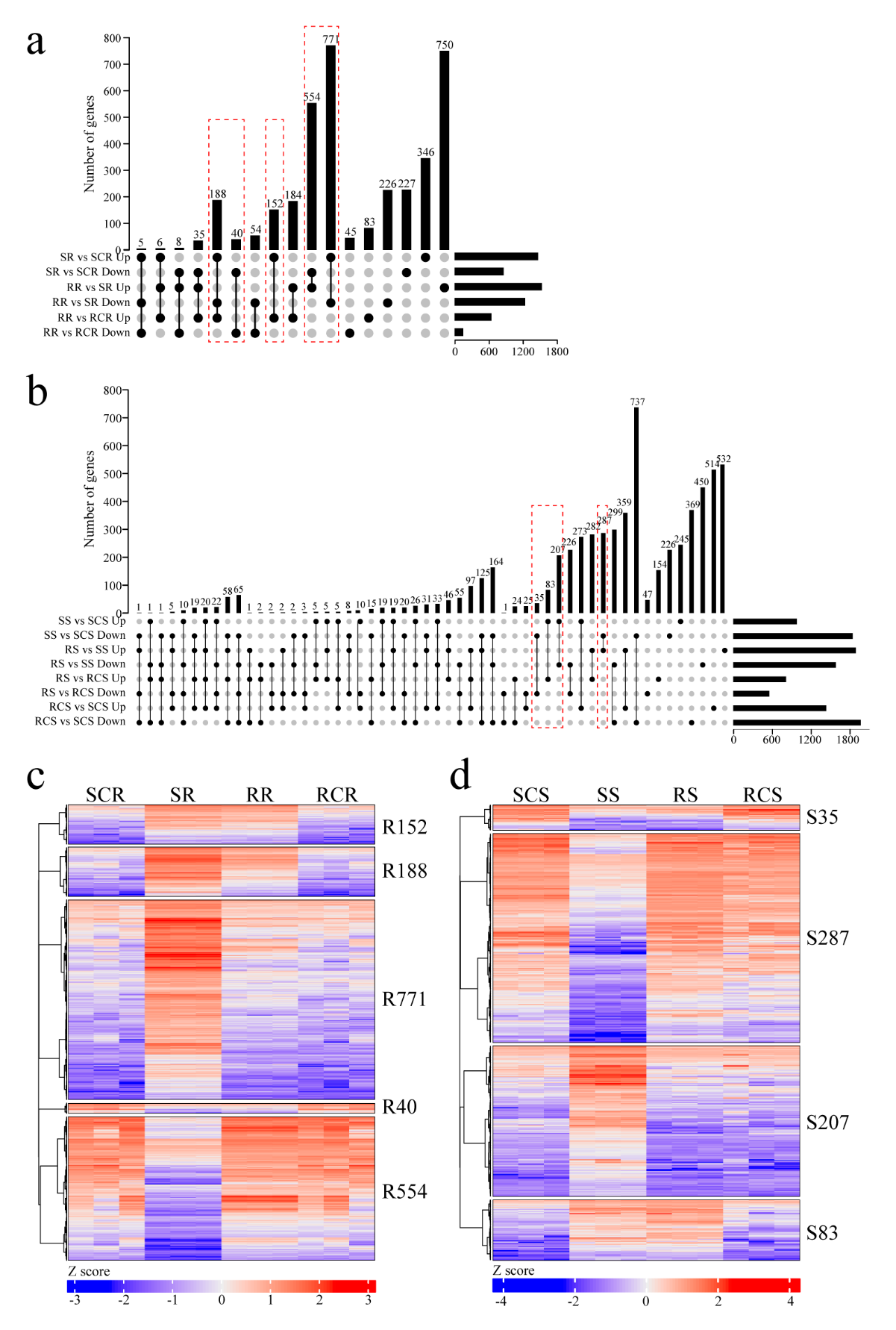


**Figure S17** Identify differentially expressed genes responding to late salt stress and recovery. **(a, b)** Upset plots of the number of upregulated and downregulated DEGs of each comparison identified in the root **(a)** and shoot **(b)** during salt stress and recovery. **(c, d)** Heatmap showing the expression of differentially expressed genes identified in the root **(c)** and shoot **(d)** that respond to responding to late salt stress and recovery.


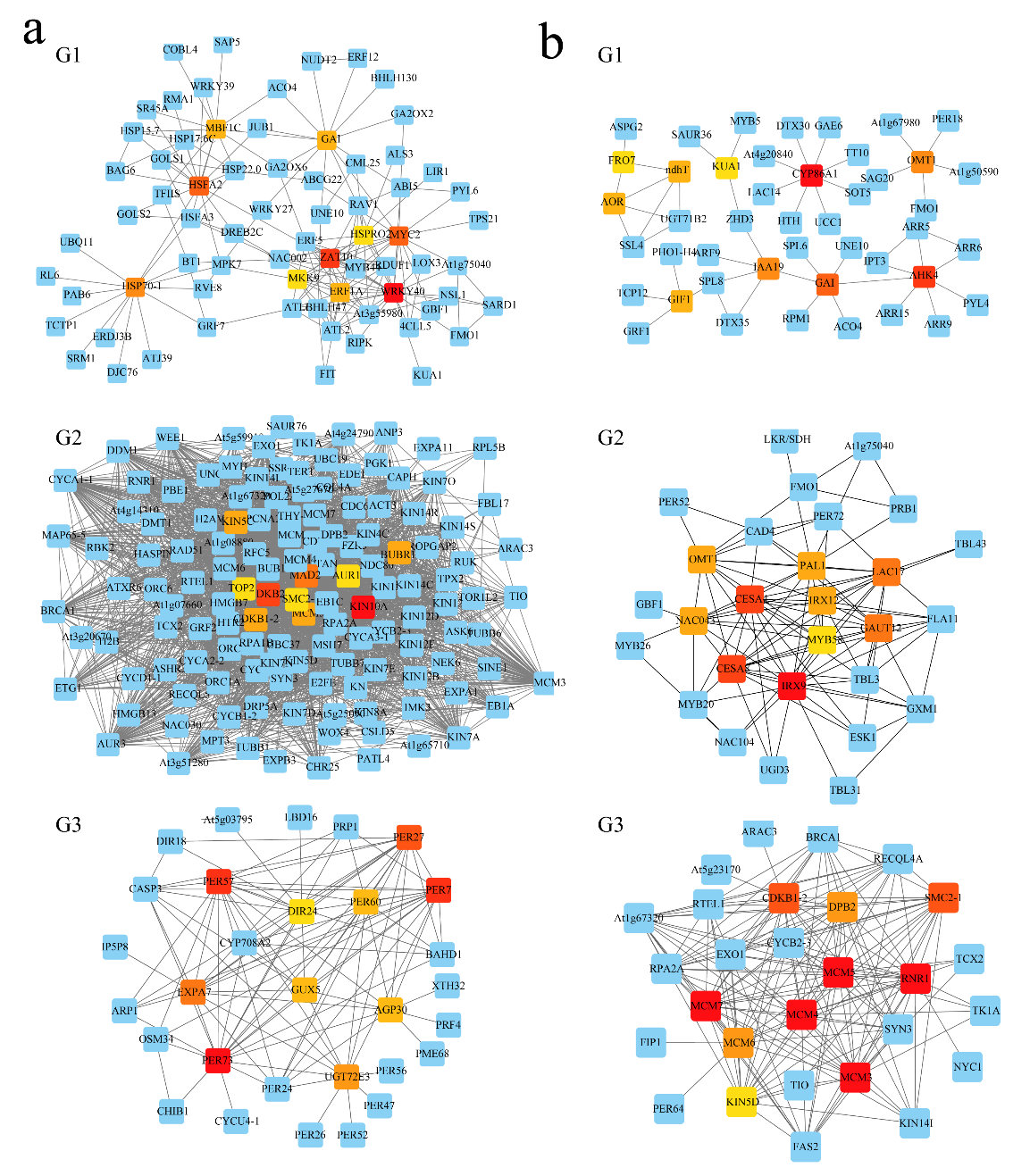


**Figure S18** Protein-protein interaction network of the DEGs of each dynamic group was constructed in the root (**a**) and shoot (**b**) during salt stress and recovery. Nodes colored red and yellow represent the hub genes, and color from red to yellow represents degree ranking.
