# Supplementary material for: Genomic analyses provide insights into the evolution and salinity adaptation of halophyte Tamarix chinensis
Source: Gigascience. 2023 Jul 26;12:giad053. doi: 10.1093/gigascience/giad053 (PMC10370455; doi:10.1093/gigascience/giad053)
Supplement: giad053_Response_to_Reviewer_Comments_Original_Submission [file giad053_response_to_reviewer_comments_original_submission.pdf]

# Author's Response To Reviewer Comments

Close

Dear Dr. Hans Zauner,
Thank you very much for your useful comments and suggestion on our manuscript entitled "Genomic analyses provide insights into the evolution and salinity adaptation of Tamarix chinensis" (GIGA-D-23-00079).

We have carefully revised the manuscript according to reviewer's comments. The point-by-point responses are below, and the revision parts in the revised manuscript have been marked in red.

We wish the revised version to be considered for publication in GigaScience.

Thanks again for your kind help.

Sincerely yours,
Ke Qiang Yang

Response to Reviewer 1 Comments

Point 1: Have authors compared these duplication events in T. chinensis with a glycophyte counterpart of T. chinensis, in order to get an idea how these duplications have evolved T. chinensis genome adapted to high salinity? Sorry, but I don't have any idea about the 5 species in the clade with which authors have compared it.
Response 1: Thanks for your question. We did not compare these duplication events in T. chinensis with a glycophyte counterpart of T. chinensis because we have yet to get a proper glycophyte counterpart of T. chinensis.
In this study, we selected 26 species in the NCORE subclade and five other species, including Beta vulgaris, Spinacia oleracea, Vitis vinifera, Solanum lycopersicum, and Oryza sativa, as the outgroup to perform all vs. all homology searches and phylogenetic orthology inference. Then, we selected five other Tamaricaceae halophyte species [1,2] belonging to the NCORE subclade with T. chinensis, including 2 Tamarix species (Tamarix ramosissima and T. hispida) and 3 Reaumuria species (Reaumuria trigyna, R. soongarica, and R. kaschgarica) to compare 4DTv (fourfold degenerate synonymous sites) and Ks (synonymous substitution rate) distributions to confirm a Tamaricaceae-specific WGD event occurred in T. chinensis. Furthermore, as you mentioned in point 3, we have analyzed the expression of WGD-mediated duplicated genes under early salt stress, late stress, and recovery treatments, suggesting several WGD-mediated duplicated genes like WRKY transcription factor coding genes (e.g., WRKY33 and WRKY75) and several genes (e.g., CBL4/SOS3, CBL10, CSC1, NHX2, AKT1, CLC-C, and CHX20) involved in stress sensing and ion homeostasis likely have evolved T. chinensis genome adapted to high salinity.

References
[1] Santos J, Al-Azzawi M, Aronson J, Flowers TJ. eHALOPH a database of salt-tolerant plants: helping put halophytes to work. Plant Cell Physiol. 2016;57(1):e10. doi: 10.1093/pcp/pcv155.
[2] Zhao, K., Song, J., Feng, G., Zhao, M., Liu, J. Species, types, distribution, and economic potential of halophytes in China. Plant Soil. 2011;342:495–509. Doi:10.1007/s11104-010-0470-7.

Point 2: In this connection, authors should also analyses a publicly available RNA-seq data-set(s) of a T. chinesis glycophyte counterpart and compare the temporal expression of hub genes (representing early, late and recovery periods of salt treatment) to see how differently T. chinensis genes behave, which could reveal its signature expression and also if this is the level of expression or time of expression as the plants strategy that makes it more tolerant.

Response 2: Thanks for your excellent suggestion. The research on the salt tolerance mechanism of T. chinesis is weak, and publicly available proper RNA-seq data-set(s) of T. chinesis and its glycophyte counterpart are few; therefore, we have yet to find any data-set(s) for comparative transcriptome analyses to see how differently T. chinensis genes behave.

Point 3: Although authors have validated the transcriptomic data by analyzing the expression of 27 random genes, I suggest authors analyses the expression of a few genes from the ones which have expanded in T. chinensis (LIMYB genes or WGD or TD-mediated duplicated genes) and which authors think might be associated with its salt tolerance, to see how their behavior is under salt stress and that if they have a functional significance.
Response 3: Thanks for your constructive suggestions. According to your suggestions, we have analyzed the expression of a few genes from those that have expanded in T. chinensis (LIMYB genes or WGD or TD-mediated duplicated genes) under salt stress, and the relevant description was added in the revised manuscript (Line 384-391, Line 403-418, Line 452-456, Line 481-485):

Line 384-391: "Integration of four differential analysis methods, including DESeq2, edgeR, ROTS, and Limma, was performed to strengthen the identification of DEGs, generating 7118 and 6023 DEGs in root and shoot during early salt exposure, respectively (Fig. 4c; Table S13-15). Of those DEGs, there were 86 EPGs, 1653 WGD genes, 631 TD genes in the root; 77 EPGs, 1433 WGD genes, and 506 TD genes in the shoot. Meanwhile, 3675 and 6272 DEGs were identified in root and shoot during late salt stress and recovery, respectively (Fig. 4c; Table S13, Table S16-17). Among the DEGs, there were 51 EPGs, 852 WGD genes, and 431 TD genes in the root; 1489 EPGs, 1433 WGD genes, and 595 TD genes in the shoot."
Line 403-418: "Of the common root DEGs, 1219 and 1473 were respectively upregulated and downregulated at 0.5, 3, 5, and 8 h salt exposure (Fig. S13a, Table S19). Among those DEGs, 11 EPGs, 329 WGD genes, and 130 TD genes exhibited upregulated expression, and 20 EPGs, 328 WGD genes, and 149 TD genes exhibited downregulated expression at 0.5, 3, 5, and 8 h salt exposure. For example, LIMYB (TC02G0567) gene showed the largest difference with a 169.76 - 256.70-fold increase among the differentially expressed EPGs at 0.5, 3, 5, and 8 h salt exposure. Nine WRKY transcription factor coding genes (e.g., WRKY33 and WRKY75) and several genes (e.g., CBL4/SOS3, NHX2, AKT1, and CHX20) involved in stress sensing and ion homeostasis showed upregulated at the four-time points. In the shoot, 961 and 1720 common DEGs were upregulated and downregulated at 5 and 8 h salt exposure, respectively (Fig. S13b, Table S20). Among the DEGs, 11 EPGs, 214 WGD genes, and 85 TD genes exhibited upregulated expression, and 12 EPGs, 450 WGD genes, and 111 TD genes exhibited downregulated expression at 5 and 8 h salt exposure. For instance, WGD genes associated with stress sensing like CBL10 and CSC1 (a Ca2+-permeable channel coding gene) and ion transport like CLC-C and potassium transporter 10 showed upregulated expressions at the two-time points."
Line 452-456: "An intersection analysis of the upregulated and downregulated DEGs in each comparison identified 1705 DEGs with opposite trends between stress and recovery or maintained at the recovery stage, which included 20 EPGs (e.g., LIMYB and RPPL1), 386 WGD genes (e.g., CSC1, HKT1, TPK1, and CHX20), and 198 TD genes (e.g., 12 class III peroxidase coding genes, including PER26, PER52, PER56, PER57, and PER60) (Fig. S17a, c, Table S23)."
Line 481-485: "Similar to the root, a total of 612 DEGs were identified, showing opposite trends between stages S and R or maintained at stage R, which included 16 EPGs (e.g., HAT and RPPL1), 154 WGD genes (e.g., GLR3.2 and Ca2+/H+ antiporter CAX1/3), and 73 TD genes (e.g., PER72, GSTT1, and GSTU8) (Fig. S17b, d, Table S27)."

Point 4: Line 254: Why did authors choose to use Arabidopsis for protein BLAST but not populus which is the model for trees?
Response 4: Thanks for your question. Compared to Populus, Arabidopsis has the most abundant protein-protein interaction (PPI) information, with most PPIs that are experimentally determined, so we choose to use Arabidopsis for protein BLAST. The relevant description has been added in the revised manuscript (Line 253-256): "Among plant species, Arabidopsis has the most abundant protein-protein interaction (PPI) data available, and most PPIs have been experimentally determined, so the protein sequences from the DEGs were BLAST-searched against the A. thaliana protein database for homolog identification."

Point 5: Authors report that genes that showed maintained up/downregulation during salt stress and recovery stages could be the 'memory genes' while the genes with opposite expression between salt stress

and recovery stages are not. I don't agree with this statement.
Although I agree that the former could be the memory genes because of their constant up/downregulation from stress to recovery phase. However, how do authors know that the expression of these genes does not dampen with time, even after 35 days? To ensure they are memory genes, authors should impose a second salt stress after recovery and see how the expression levels of these genes change. Moreover, the genes in the latter category (opposite expression between salt stress and recovery stages) can be 'memory genes' as well. We know that first stress causes epigenetic changes at memory loci and these loci get imprinted during the recovery which upon recurrent stress give rise to a modified response. So, pls revisit this conclusion based on a second salt stress.
Response 5: Thanks for your excellent comments. The description of memory genes is inaccurate, and the revised manuscript has corrected the relevant description.

Point 6: If T. chinensis remains/grows constantly in the coastal wetland ecosystem, then what is the physiological/ecological significance of these potentially salt memory genes for this plant, when the stress is constant and not recurring.? Pls elaborate.
Response 6: Thanks for your excellent comment. It is a crucial evolutionary ecology topic that needs to be elaborated on based on extensive provenances of the tree species from different habitats. The genomic and salt stress transcriptomic data were obtained only from the Tamarix chinensis cultivar ‘Lucheng No.1’ in our manuscript, which cannot support this topic. Your suggestion has provided us with a good idea for our future research. Thank you again.

Point 7: Lastly, to make the title catchier and more discoverable, add halophyte in the title as: Genomic analyses provide insights into the evolution and salinity adaptation of recretohalophyte Tamarix chinensis.
Response 7: Thanks for your suggestion. We agree to add "halophyte" in the title: “Genomic analyses provide insights into the evolution and salinity adaptation of halophyte Tamarix chinensis” in the revised manuscript. Tamarix chinensis exhibits multiple pathways responding to salt stress. It was found that root and shoot showed distinct gene expression profiles to salt stress in this manuscript. Meanwhile, more scientific evidence is needed for the mechanism of salt secretion in the scale leaf and shoot of Tamarix chinensis.

Point 8: Line 37: should be "…salt stress were identified".
Response 8: Following your suggestion. We have changed "…salt stress and identified" to "…salt stress were identified" in the revised manuscript (Line 37).

Point 9: Line 43: it is better to write 'genomic resources' than genetic resources.
Response 9: Thanks for your suggestion. In the revised manuscript, we have changed "genetic resources" to "genomic resources" (Line 44).

Point 10: Line 81-82: "…which the plant has various applications such as environmental governance and landscape value". Pls rewrite this bit.
Response 10: Thanks for your comment. We have removed the relevant description in the revised manuscript.

Point 11: Line 84: should be "In addition, because of its high tolerance to salt…".
Response 11: Following your suggestion, we have changed the description "In addition, as its high tolerance to salt…" to "In addition, because of its high tolerance to salt…" in the revised manuscript (Line 82).

Point 12: Line 89: "Dissecting the whole genome of plants with high salt tolerance" should be "Dissecting the whole genome of plants for different salt tolerance genomic resources…".
Response 12: Thanks for your suggestion. We have changed the description "Dissecting the whole genome of plants with high salt tolerance" to "Dissecting the whole genome of plants for different salt-tolerance genomic resources…" in the revised manuscript (Line 86).

Point 13: Line 94: better to write genomic resources.
Response 13: Following your suggestion. We have changed "genetic resources" to "genomic resources" in the revised manuscript (Line 92).

Point 14: Line 377 and 383: should be "…early salt stress, late salt stress, and recovery…".
Response 14: Thanks for your suggestion. In the revised manuscript, we have changed the description "…early salt stress, and late stress and recovery…" to "…early salt stress, late salt stress, and recovery…" (Line 377 and 383).

Point 15: Line 387-388: add ", respectively" after "and recovery".
Response 15: Following your suggestion, we have added ", respectively" after "and recovery" in the revised manuscript (Line 389).

Point 16: Line 387, 395, 412, 418 and so on: pls don't use "at roots, at shoots". Instead use "in roots or in shoots". Correct all such things in the MS.
Response 16: Thanks for your suggestion. We have changed the description "at roots, at shoots" to "in roots, in shoots" throughout the text in the revised manuscript.

Point 17: Line 400: should be "Of the common root DEGs, 1219 and 1473 were respectively upregulated and downregulated at 0.5, 3, 5, and 8 h of salt exposure (Table...".
Response 17: Following your suggestion, we have changed the description in the revised manuscript (Line 403-404).

Point 18: Line 416: "in response to salt stress exposure to early salt stress" doesn't make any sense.
Response 18: Thanks for your suggestion. We have deleted the description "...involved in response to salt stress exposure to early salt stress" in the revised manuscript.

Response to Reviewer 2 Comments
Point 1: I would have liked to have a summary figure with the expression of the genes according to the salinity conditions. This could have helped in understanding.
Response 1: Thanks for your suggestion. In this study, we have provided Fig. 5e and f to show the hub genes identified in the root (Fig. 5e) and shoot (Fig. 5f) responding to early salt stress, and Fig. 6e and f to show the hub genes identified in the root (Fig. 5e) and shoot (Fig. 5f) that respond to late salt stress and recovery in T. chinensis. Following your suggestion, in order to show the expression of genes according to the salinity conditions more intuitively, we added Fig. S13 and Fig. S17c, d to show the expression of differentially expressed genes identified in the root and shoot responding to early salt stress (Fig. S13) and late salt stress and recovery (Fig. S17c, d).

Point 2: I appreciated the effort to conduct qPCR experiments to verify the DEGs identification. But I'm not sure that the authors can speak about strong correlation coefficient ($R^2$=0.41).
Response 2: Thanks for your comment. The relative description has been rephrased as "A randomly selected 27 DEGs were verified by qRT-PCR (Table S18), resulting in a significant and positive correlation between transcriptome results and qRT-PCR data, indicating the transcriptome data are reliable (Fig. 4d)." in the revised manuscript (Line 392-394).

Point 3: Did the authors check the presence of telomeric repeats at the extremities of the biggest scaffolds?
Response 3: Thanks for your question. We checked the presence of telomeric repeats at the extremities of 12 pseudochromosomes in the T. chinensis assembly using tidk v. 0.2.31 (https://github.com/tolkit/telomeric-identifier) with the find command and the following parameters: "-w 10000 -c Caryophyllales", showing that pseudochromosome 1, 6, 9, 10, and 12 respectively with only one end matched the known telomeric repeat for Caryophyllales clade. We agree that checking the presence of telomeric repeats at the extremities of chromosomal sequences is a necessary evaluation indicator for the telomere-to-telomere (T2T) genome assembly. Because of the non-T2T genome sequencing and assembly strategy used in our study, the relevant results of this evaluation were not present in this manuscript.

Point 4: The merqury score is commonly used to estimate the quality of the consensus (https://genomebiology.biomedcentral.com/articles/10.1186/s13059-020-02134-9).
Response 4: Thanks for your suggestion. We performed genome assembly quality evaluation using Merqury with k = 25 based on Illumina short-reads, and the relative description has been added in the revised

manuscript (Line 163-164 and Line 288-291):
Line 163-164: "Finally, Merqury v. 1.3 [54] with the parameter: k = 25 estimated the assembly quality value (QV)."
Line 288-291: "At last, Merqury estimated the assembly base accuracy and completeness, resulting in a high QV of 39.03 (Table 1). The above results suggested that the assembled T. chinensis genome was high quality in genome completeness, baseline accuracy, and contiguity."

Point 5: Line 156: « All software was executed under the parameters. » which parameters?
Response 5: Thanks for your question. All software was executed with default parameters, and the relative description was added in the revised manuscript (Line 154).

Close
