# Supplementary material for: Genomic analyses provide insights into the evolution and salinity adaptation of halophyte Tamarix chinensis
Source: Gigascience. 2023 Jul 26;12:giad053. doi: 10.1093/gigascience/giad053 (PMC10370455; doi:10.1093/gigascience/giad053)
Supplement: giad053_Reviewer_1_Report_Original_Submission — Showkat Ganie -- 4/10/2023 Reviewed [file giad053_reviewer_1_report_original_submission.pdf]

**Reviewer Report**

**Title: Genomic analyses provide insights into the evolution and salinity adaptation of Tamarix chinensis**

**Version: Original Submission** **Date:** 4/10/2023

**Reviewer name: Showkat Ganie**

**Reviewer Comments to Author:**

In this MS, Liu and colleagues performed a genomic investigation of T. chinensis to elucidate the potential mechanisms of its high salt tolerance. They have found that families of HAT and LIMYB genes are significantly expanded in this plant, mediated by whole-genome and tandem duplications and report that this expansion of genes is associated with the salinity adaptation of T.chinensis. Further, through transcriptome analysis, they found several hub genes responding to early, late and recovery phases of salt stress in root and shoot tissues.

The findings will be a useful addition to the literature on the halophytic nature of T. chinensis. From the agricultural practice perspective, this research, although observational in nature, might have some implications regarding the salt tolerance breeding. For this reason, the work reported in the MS certainly fits well the scope of the journal.

On the other hand, this work although lays the foundation for future studies of the underlying salt tolerance mechanisms in T. chinenesis, there are certain things which need to be addressed. If authors could gather the following information, it would be more insightful.

1. Have authors compared these duplication events in T. chinensis with a glycophyte counterpart of T. chinensis, in order to get an idea how these duplications have evolved T.chinensis genome adapted to high salinity? Sorry, but I don't have any idea about the 5 species in the clade with which authors have compared it.
2. In this connection, authors should also analyse a publicly available RNA-seq data-set(s) of a T-chinesis glycophyte counterpart and compare the temporal expression of hub genes (representing early, late and recovery periods of salt treatment) to see how differently T-chinensis genes behave, which could reveal its signature expression and also if this is the level of expression or time of expression as the plants strategy that makes it more tolerant.
3. Although authors have validated the transcriptomic data by analysing the expression of 27 random genes, I suggest authors analyse the expression of a few genes from the ones which have expanded in T. chinensis (LIMYB genes or WGD or TD-mediated duplicated genes) and which authors think might be associated with its salt tolerance, to see how their behavior is under salt stress and that if they have a functional significance.
4. Line 254: Why did authors choose to use Arabidopsis for protein BLAST but not populus which is the model for trees?
5. Authors report that genes that showed maintained up/downregulation during salt stress and recovery stages could be the 'memory genes' while the genes with opposite expression between salt stress and recovery stages are not. I don't agree with this statement.

Although I agree that the former could be the memory genes because of their constant up/downregulation from stress to recovery phase. However, how do authors know that the expression of these genes does not dampen with time, even after 35 days? To ensure they are memory genes, authors should impose a second salt stress after recovery and see how the expression levels of these genes change. Moreover, the genes in the latter category (opposite expression between salt stress and recovery stages) can be 'memory genes' as well. We know that first stress causes epigenetic changes at memory loci and these loci get imprinted during the recovery which upon recurrent stress give rise to a modified response. So, pls revisit this conclusion based on a second salt stress.
6. If T. chinensis remains/grows constantly in the coastal wetland ecosystem, then what is the physiological/ecological significance of these potentially salt memory genes for this plant, when the stress is constant and not recurring.? Pls elaborate.
7. Lastly, to make the title catchier and more discoverable, add halophyte in the title as: Genomic analyses provide insights into the evolution and salinity adaptation of recretohalophyte Tamarix chinensis
Some minor suggestions:
Pls correct typos and grammar at several places.
For example, Line 37: should be "…salt stress were identified".
Line 43: it is better to write 'genomic resources' than genetic resources.
Line 81-82: "…which the plant has various applications such as environmental governance and landscape value". Pls rewrite this bit.
Line 84: should be "In addition, because of its high tolerance to salt…"
Line 89: "Dissecting the whole genome of plants with high salt tolerance" should be "Dissecting the whole genome of plants for different salt tolerance genomic resources…"
Line 94: better to write genomic resources.
Line 377 and 383: should be "…early salt stress, late salt stress, and recovery…"
Line 387-388: add ", respectively" after "and recovery".
Line 387, 395, 412, 418 and so on: pls don't use "at roots, at shoots". Instead use "in roots or in shoots". Correct all such things in the MS.
Line 400: should be "Of the common root DEGs, 1219 and 1473 were respectively upregulated and downregulated at 0.5, 3, 5, and 8 h of salt exposure (Table..."
LINE 416: "in response to salt stress exposure to early salt stress" doesn't make any sense.

**Methods**

Are the methods appropriate to the aims of the study, are they well described, and are necessary controls included? Choose an item.

**Conclusions**

Are the conclusions adequately supported by the data shown? Choose an item.

**Reporting Standards**

Does the manuscript adhere to the journal's guidelines on minimum standards of reporting? Choose an item.

Choose an item.

**Statistics**

Are you able to assess all statistics in the manuscript, including the appropriateness of statistical tests used? Choose an item.

**Quality of Written English**

Please indicate the quality of language in the manuscript: Choose an item.

**Declaration of Competing Interests**

Please complete a declaration of competing interests, considering the following questions:

- Have you in the past five years received reimbursements, fees, funding, or salary from an organisation that may in any way gain or lose financially from the publication of this manuscript, either now or in the future?
- Do you hold any stocks or shares in an organisation that may in any way gain or lose financially from the publication of this manuscript, either now or in the future?
- Do you hold or are you currently applying for any patents relating to the content of the manuscript?
- Have you received reimbursements, fees, funding, or salary from an organization that holds or has applied for patents relating to the content of the manuscript?
- Do you have any other financial competing interests?
- Do you have any non-financial competing interests in relation to this paper?

If you can answer no to all of the above, write 'I declare that I have no competing interests' below. If your reply is yes to any, please give details below.

I declare that I have no competing interests

I agree to the open peer review policy of the journal. I understand that my name will be included on my report to the authors and, if the manuscript is accepted for publication, my named report including any attachments I upload will be posted on the website along with the authors' responses. I agree for my report to be made available under an Open Access Creative Commons CC-BY license (http://creativecommons.org/licenses/by/4.0/). I understand that any comments which I do not wish to be included in my named report can be included as confidential comments to the editors, which will not be published.

Choose an item.

To further support our reviewers, we have joined with Publons, where you can gain additional credit to further highlight your hard work (see: https://publons.com/journal/530/gigascience). On publication of this paper, your review will be automatically added to Publons, you can then choose whether or not to claim your Publons credit. I understand this statement.

Yes Choose an item.
